# Synthesis and Antiproliferative Activity of Novel Imipridone–Ferrocene Hybrids with Triazole and Alkyne Linkers

**DOI:** 10.3390/ph15040468

**Published:** 2022-04-12

**Authors:** Tamás Czuczi, József Murányi, Péter Bárány, István Móra, Adina Borbély, Miklós Csala, Antal Csámpai

**Affiliations:** 1 Department of Organic Chemistry, Eötvös Loránd University (ELTE), Budapest Pázmány P. Sétány 1/A, H-1117 Budapest, Hungary; czuczi.tamas@gmail.com (T.C.); peterbarany@ceasar.elte.hu (P.B.); 2 MTA-SE Pathobiochemistry Research Group, Tűzoltó u. 37-47, H-1094 Budapest, Hungary; jozsefmuranyi84@gmail.com (J.M.); istvan.mora1313@gmail.com (I.M.); csala.miklos@med.semmelweis-univ.hu (M.C.); 3 MTA-ELTE Lendület Ion Mobility Mass Spectrometry Research Group, Department of Analytical Chemistry, Eötvös Loránd University (ELTE), Budapest Pázmány P. Sétány 1/A, H-1117 Budapest, Hungary; adina.borbely@ttk.elte.hu; 4 Department of Molecular Biology, Semmelweis University, Tűzoltó u. 37-47, H-1094 Budapest, Hungary

**Keywords:** ferrocene, ONC201, resistance, pancreatic cancer, PANC-1, cancer therapy, drug design

## Abstract

Imipridones, including ONC201, ONC206 and ONC212 (which are emblematic members of this class of compounds developed by Oncoceutics) constitute a novel class of anticancer agents, with promising results in clinical trials. With the aim of increasing the ROS (reactive oxygen species) responsivity of the synthesized molecules, a set of novel ferrocene–imipridone hybrids were designed and synthesized. Our strategy was motivated by the documented interplay between the imipridone-triggered activation of TRAIL (the tumor necrosis factor-related apoptosis-inducing ligand) and mitochondrial ClpP (Caseinolytic protease P) and the ROS-mediated effect of ferrocene-containing compounds. In order to obtain novel hybrids with multitarget characters, the ferrocene moiety was tethered to the imipridone scaffold through ethynylene and 1,2,3-triazolyl linkers by using Sonogashira coupling of Cu(I)- and Ru(II)-catalyzed azide–alkyne cycloadditions. The biological activities of the new hybrids were examined by using in vitro cell viability assays on four malignant cell lines (PANC-1, A2058, EBC-1 and Fadu), along with colony formation assays on the most resistant PANC-1 cell line. Several hybrids caused a significantly greater drop in the cell viability compared to ONC201, and two of them completely overcame the resistance, with IC_50_ values comparable to those produced by ONC201. The two most potent hybrids, but not ONC201, induced apoptosis/necrosis in PANC-1 and A2058 cells after 24 h of treatment.

## 1. Introduction

**Figure 1:**
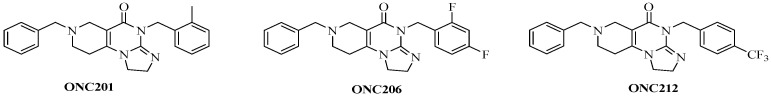
Reference imipridones with numbering of the substituted skeletal nitrogen atoms.

ONC201, the first-in-class member of imipridones, which are a novel family of anticancer agents, is currently in Phase I/II clinical trials for solid and hematologic cancers of the CNS (central nervous system). Other imipridones, such as ONC206 and ONC212, were also identified as highly potent anticancer agents (Figure 1) that displayed enhanced in vitro and in vivo efficacy compared to ONC201 [1].

Despite the favorable results with ONC201 in clinical trials against a wide range of advanced malignancies, and the promising anticancer properties of other imipridones, the mechanisms of action of these compounds are not yet fully established. A recent review by Bonner et al. [2] highlights that, along with multiple pathways, such as TRAIL signaling, dopamine receptor D2 (DRD2) antagonism, G protein-coupled receptor 132 (GPCR-GPR132) agonism and mitochondrial metabolism, more precisely, the mitochondrial caseinolytic protease P (ClpP) may be the major target of imipridones.

While, normally, ClpP is regulated by caseinolytic mitochondrial matrix peptidase chaperone subunit X (ClpX), the activation of ClpP by imipridones causes an unregulated increase in the degradation of mitochondrial proteins, which leads to the structural and functional damage of the mitochondria and, thus, to the suppression of oxidative phosphorylation. The degradation of mitochondrial proteins is accompanied by, among others, a lower oxygen consumption rate, decreased levels of ATP and mitochondrial DNA, and an increased amount of mitochondrial reactive oxygen species (ROS). These effects activate the integrated stress response (ISR), and eventually arrest the cell cycle and induce the process of apoptosis [2].

However, it should be highlighted that, despite the promising antiproliferative and proapoptotic effects of imipridones, the resistance of cancer cells to the treatment seems to be a persistent problem. In this regard, several studies [3,4,5,6,7,8,9,10,11] have identified imipridones as effective cytotoxic agents in micro- or nanomolar concentrations; however, during the course of the cell viability screenings, approximately 10–50% of the cells survived the treatment, even at higher imipridone concentrations and after prolonged exposure times.

As mentioned above, imipridones cause the structural damage of mitochondria and elevated levels of mitochondrial ROS [2]. ROS are generated naturally in mitochondrial oxidative phosphorylation, as well as in peroxisomes and the endoplasmic reticulum, but their amount is tightly regulated by intracellular antioxidant programs [12]. Low levels of mitochondrial ROS have important roles in signaling and in the regulation of the protein function, and they are required for normal cell activity [13,14,15,16]. Elevated ROS production has been detected in various cancers, including in melanoma, glioma, pancreatic, prostate, breast, ovarian, colon and bladder cancer [17], and it has been shown to play several roles, which include the activation of protumorigenic signaling, the enhancement of cell survival and proliferation and the driving of DNA damage, and the furthering of genetic instability. Because of the increased ROS production, tumor cells express elevated levels of antioxidant proteins to detoxify the excessive ROS, thereby establishing a redox balance while maintaining protumorigenic signaling and resistance to apoptosis and, thus, to chemotherapy [13]. Nevertheless, if ROS levels exceed the optimum concentration in healthy or neoplastic cells, it can exhaust and ‘overpower’ the antioxidant programs, lead to oxidative stress and can induce apoptosis through both extrinsic and intrinsic pathways [16]. For this reason, compounds that promote ROS formation in cancer cells could be considered as potential anticancer agents [12,13,14,15,16,17,18,19,20,21,22,23,24], including ferrocene-containing organometallics [21,22,23,24].

Ferrocenyl compounds tend to be nontoxic and chemically stable, and their redox character can be fine-tuned, which may play a vital role in the generation of mitochondrial ROS. There are several examples of ferrocene-containing bioactive compounds that exhibit anticancer, antifungal and antimalarial effects. The most well-known are ferroquine and ferrocifen. As recently summarized by Peter and Aderibigbe [22], other anticancer ferrocene derivatives include ferrocene–indole (against the A549 human lung carcinoma cell line), 1,2,4-trioxane-ferrocene hybrids (against leukemia cells), ferrociphenols (against the MCF7 and MDA-MB231 breast cancer cell lines), ferrocenyl derivatives of clotrimazole and ferrocene conjugates of chalcones and steroids.

The ferrocenyl group undergoes a Fenton-like reaction under physiological conditions, which leads to the generation of ROS. High levels of ROS, as mentioned above, can disrupt normal cellular processes through a nonspecific attack on proteins, lipids and DNA, which causes cell death and the suppression of tumor growth [21,22,23,24].

Previously, our research group synthesized a series of such imipridones, in which the skeletal *N*-benzyl substituents were replaced by the ferrocenylalkyl groups. The hybrids were found to exhibit much more pronounced long-term cytotoxic effects against the A-2058 melanoma cell line than ONC201 and ONC212, which is possibly due to the contribution of the ROS-implicating apoptotic pathways that are activated by the ferrocenyl moieties. Furthermore, these ferrocenylalkyl-substituted imipridones also displayed a marked efficacy against the COLO-205 and EBC-1 cell lines [25]. At this stage, it must be pointed out that convincing preclinical evidence has been disclosed on the interplay between the TRAIL and redox signaling pathways that is implicated in cancer [26]. Prompted by these precedents and by the anticipated synergistic effects of ferrocene-driven oxidative stress and imipridone-induced ClpP and TRAIL activation [27], we undertook to construct and evaluate a small library of novel and potentially proapoptotic organometallic imipridone hybrids, which contain ferrocene fragments that are tethered by triazole and/or alkyne linkers at different positions of the *N*-benzyl groups on the terminal regions of the heterocyclic skeleton.

We hypothesized that the dual promotion of ROS formation in the mitochondria by the ferrocene moiety and the imipridone scaffold in the designed hybrids leads to a more efficient decrease in the cancer cell viability and, more importantly, can eliminate the resistance that is often experienced to imipridone treatment.

## 2. Results and Discussion

### 2.1. Synthesis of the Reference and Hybrid Imipridones

In the designed compounds, the ferrocenyl moiety is connected to the terminal aromatic rings (Figure 1) of the imipridone scaffold via a 1,2,3-triazole or an ethynylene linker.

**Scheme 1:**
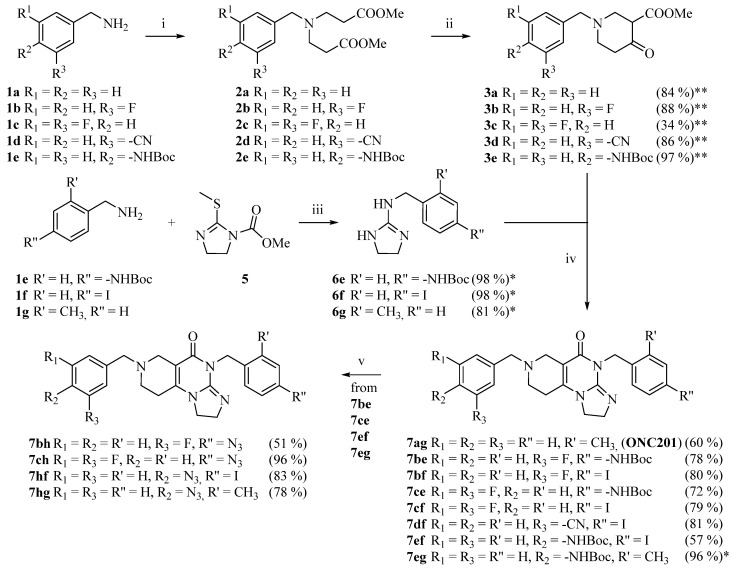
Synthetic route to the imipridone scaffold reaction conditions: (i) methyl acrylate (5 eq.), MeOH, r.t., 48 h; (ii) NaH (5 eq.), THF, 0 °C, then r.t., 2 h; (iii) MeOH/AcOH = 4/1, reflux, 18 h. (iv) 3.6 M NaOMe/MeOH (1.25 eq.), MeOH, reflux, 12 h. (v) 1) cc. HCl, r.t., 30 min. 2) NaNO_2_ (2 eq.), 0 °C, 45 min. 3) NaN_3_ (10 eq.), 0 °C, 45 min, then r.t., 1 h. * = crude product; ** = cumulative yield, crude product.

The targeted imipridones were synthesized by using a well-established pathway that is described in our previous work [25], as shown in Scheme 1. The commercially available primary amines (**1a**–**e**) were reacted with an excess amount of methyl acrylate in methanol at room temperature to obtain intermediates (**2a**–**e**), which were then cyclized by sodium hydride, which afforded the *N*-substituted piperidone carboxylates (**3a**–**e**). Primary amine building blocks (**1e**–**g**) were also reacted with activated methyltioimidazoline (**5**) to obtain cyclic guanidines (**6e**–**g**), which were then cyclized with piperidone carboxylates (**3a**–**e**) under standard conditions to access the imipridones, **7ag**, **7be**, **7bf**, **7ce**, **7cf**, **7df**, **7ef** and **7eg**. The derivatives carrying a Boc-protected amino group on either of the aromatic rings (**7be**, **7ce**, **7ef** and **7eg**) were used as precursors to obtain the appropriate azido derivative (**7bh**, **7ch**, **7hf** and **7hg**) by deprotection, diazotation and sequence-terminating azide introduction.

**Scheme 2:**
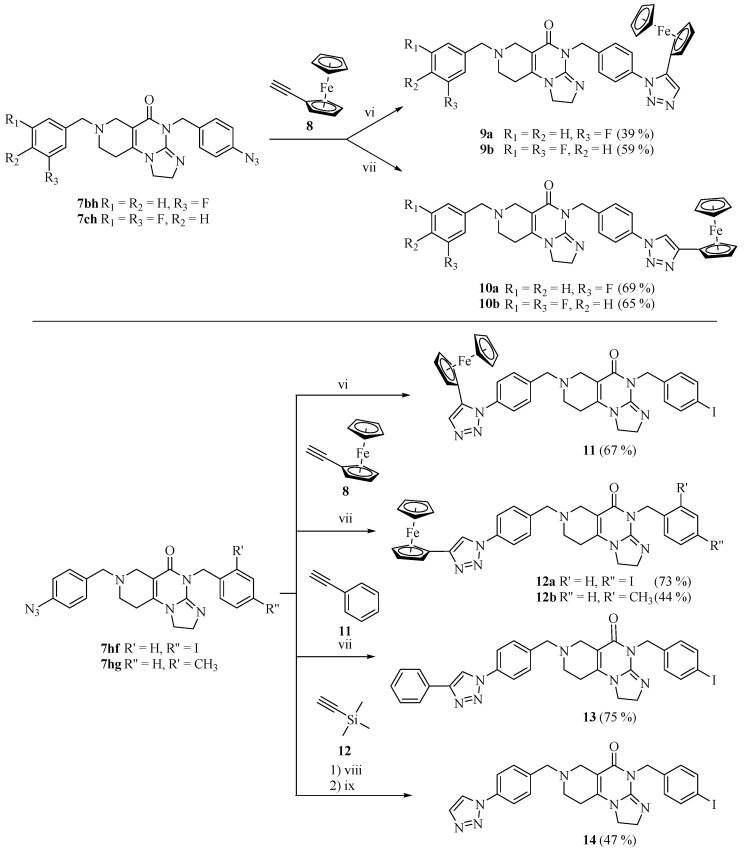
Azide–alkyne cycloadditions. Reaction conditions: (vi) 5% Cp*RuCl(PPh_3_)_2_, DMF, r.t., 12 h; (vii) CuI (1 eq.), DMSO, r.t., 12 h; (viii) CuI (1 eq.), CuSO_4_ (0.5 eq.), Na-*L*-ascorbate (1 eq.), H_2_O (0.1 mL), DMSO, r.t., 12 h, and then excess trimethylsilyl acetylene, 80 °C, 2 h; (ix) TBAF (2 eq.), THF, r.t., 1.5 h.

**Scheme 3:**
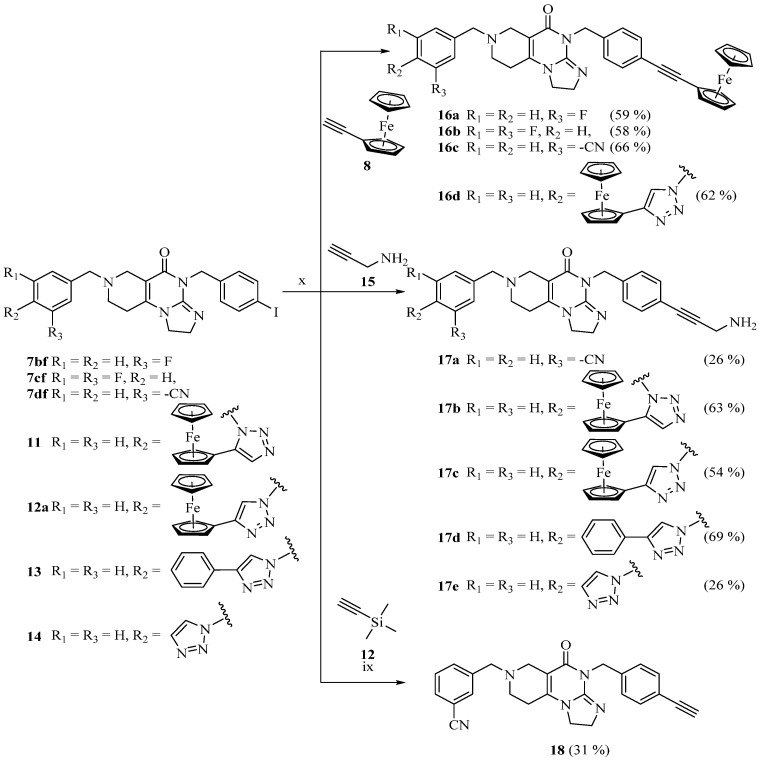
Sonogashira couplings. Reaction conditions: (ix) TBAF (2 eq.), THF, r.t., 1.5 h; (x) 20% CuI, 10% PdCl_2_ (PPh_3_)_2_, DIPEA (5 eq.), DMF, r.t., 24 h.

The introduction of the ferrocenyl group was achieved by transition-metal (copper- and ruthenium)-catalyzed azide–alkyne cycloadditions [28,29,30] and the Sonogashira coupling of the commercially available ethynylferrocene with the corresponding azido- and iodo-functionalized imipridones, respectively, to assemble the targeted triazole- and alkyne-tethered organometallic hybrids (Scheme 2 and Scheme 3).

It should be noted that Compound **7hf**—which carries 4-N_3_ and 4-I substituents on the aromatic rings—participated exclusively in azide–alkyne cycloadditions, without being accompanied by undesired side reactions. The iodoaryl group remained intact and, thus, it could be used for the Sonogashira coupling in the following step.

To examine the effect of the ferrocenyl group, reference compounds were also synthesized. In these molecules, the ferrocenyl group connected to the triazole-ring (in **12a** and **17c**) was replaced with the phenyl group (**13**, **17d**) or was left unsubstituted (**14**, **17e**). These derivatives were prepared by using phenylacetylene or trimethylsilylacetylene in copper-catalyzed azide–alkyne cycloaddition protocols. The removal of the trimethylsilyl-protecting group was carried out by tetra-*n*-butylammonium fluoride (TBAF) in THF.

### 2.2. Cell Viability Screenings of the Imipridone Analogues

Cell viability screenings were performed on PANC-1 (pancreatic ductal adenocarcinoma), A2058 (melanoma), EBC-1 (lung squamous carcinoma) and Fadu (hypopharynx squamous carcinoma) human cancer cell lines at three concentrations (2.8, 8.3 and 25 µM) to determine the structure–activity relationship and to narrow down the range of compounds for further investigations. The results of the viability screenings are shown in Table 1.

### 2.3. Structure–Activity Relationships

It can be clearly seen that the cells treated with the reference compound, ONC201, had a consistent reduction in viability at all three concentrations; however, depending on the cell line, a significant portion survived the treatment (47–63% for PANC-1 and A2058, 31–37% for EBC-1 and 16–25% for Fadu). The structure of Compound **18** is the most closely related to that of ONC201 in terms of the steric bulk, even though it contains electron-withdrawing groups on the aromatic rings. Nevertheless, the cell viability results of **18** indicate no significant difference between **18** and ONC201 at these concentrations.

However, upon treatment with compounds **17b**–**d** at a concentration of 25 µM, the viability of the investigated cell lines dropped down to 1–3%. All of the three models, with such remarkable efficiency, are 4-(4-(3-aminoprop-1-yn-1-yl)benzyl)-substituted imipridones that carry a triazole ring at the *para* position on the benzyl group at the *N*-7 position of the angular tricyclic scaffold. A comparison of the performance of the ferrocene-containing hybrids, **17b** and **17c,** indicates that the 1,4-disubstitution of the triazole ring is more favorable than the alternative 1,5 substitution pattern. Since **17d** contains a phenyl instead of a ferrocenyl group on the 1,4-disubstituted triazole ring, the comparison of the results that were obtained at an imipridone concentration of 25.0 µM would apparently suggest that it is not the ferrocenyl moiety that is prominently responsible for the low viability of the cells; however, at a concentration of 8.3 µM, the organometallic counterpart, **17c,** performed better on all four cell lines compared to **17d**.

Compound **17e** induced only a small decrease in the cell viability at the highest concentration, and little-to-no decrease at lower concentrations, which proves that the substitution of the triazole ring is crucial for achieving low cell viability.

From the aspect of structure–activity relationships (SAR), it is of importance that Compounds **11**, **12a,b**, **13** and **16d**, which are also decorated with triazole-based substituents on the *para* position of the *N*-7-benzyl group, do not display effects that are similar to those that are produced by **17b**–**d**, which proves that the *para*-aminopropynyl-substituted benzyl group on the *N*-4 skeletal atom is also necessary for the production of significant antiproliferative activity. On the other hand, the viability assays also indicate that, at least in combination with the triazolylbenzyl substituents on the *N*-7 skeletal position, the introduction of the 2-methyl-, 4-iodo- or 4- ferrocenylethynyl group in the benzyl group, depending on the *N*-4 skeletal atom, is not beneficial for lowering the cell viability. It must be pointed out here that, although, in the case of the isomer pair, **17b/17c**, the 1,4-disubtitution on the triazole ring was found favorable over the 1,5-disubstitution pattern. Among the triazole derivatives, **11**, **12a,b**, **13** and **16d**, the experiments on the A2058 and EBC-1 cell lines identified **11**, a 4-(4-iodobenzyl)-containing imipridone, as the most effective model, with the 4-(5-ferrocenyl-1*H*-1,2,3-triazol-1-yl)benzyl group attached to the *N*-7 position instead of its isomer, **12a**, which carries the 4-(4-ferrocenyl-1*H*-1,2,3-triazol-1-yl)benzyl group on the *N*-7 skeletal atom.

Demonstrating a further SAR, compounds **9a,b**, which are equipped with the 1,4 disubstituted triazolyl-substituted benzyl group at Position 4, caused only a little-to-no decrease in the cell viability, whereas **10a,b**, which contain the 1,5-disubstituted triazole ring on the benzyl group that is attached to the same (*N*-4) position, induced a more pronounced decrease in the viability of the investigated cell lines. In fact, **10a** was able to affect a substantial drop in the viability of the EBC-1 cells, down to the level (3%) at 25 μM, which was achieved by **17b**–**d** at the same concentration. (Pointing to the cell selectivity, the treatment with **10a** also led to the relatively low viability of the other cell lines at this concentration, but not as low as was achieved by **17b**–**d**).

The compounds with a ferrocenylethynyl group at the *para* position on the benzyl group that is attached to the *N*-4 atom (**16a**–**c**) showed moderate-to-no decrease and, interestingly, on PANC-1, even showed an increase in the cell viability. The increment in the viability might be ascribed to the fact that pancreatic cancer cells naturally have an elevated ROS level, and a small elevation in the ROS concentration can even be beneficial in attenuating their proliferation [16]. This tendency has also been revealed for other cell lines [17] that are associated with less pronounced increments in their viability.

As mentioned above, Compound **18** exhibited very similar effects to those of ONC201. Interestingly, **17a**, which also carries the 3-cyanobenzyl group on *N*-7 and the 4-(3-aminoprop-1-yn-1-yl)benzyl group, an extended alkyne residue on *N*-4 gave rise to an appreciable drop in the viability of all of the studied cells to markedly lower levels, relative to those induced by **18** and ONC201. This means that, cooperating with the aryltriazolyl moiety that pends on the benzyl group at the *N*-7 position, the presence of the aminopropynyl group on the benzyl group at the opposite *N*-4 position is necessary to attain very low-level or nearly complete loss of viability. Thus, the aforementioned SAR could also imply that: (i) Compounds **17b**–**d** act on a molecular target that is different from those identified for ONC201 and other emblematic imipridones; and (ii) The arytriazolyl and aminopropynyl groups, which are appropriately located on the two terminals of the imipridone framework, are necessary for cooperatively creating effective interactions with target(s) undisclosed so far. It is of note that the abovementioned substituent patterns seem incompatible with the known imipridone targets, with special regard to ClpP. This hypothesis is also supported by the fact that the PANC-1 (pancreas) and A2058 (melanoma) cells were less susceptible to ONC201 treatment than the Fadu (hypopharynx) or EBC-1 (lung) cancer cells; however, for the treatment with Compounds **17b**–**d**, the sensitivity of the four cell lines showed much less significant differences. Furthermore, during treatment with these compounds, especially in the case of the PANC-1 cell line, the cell viability dropped more rapidly with the same increment in concentration, which also points to the involvement of different biological target(s).

Since Compounds **10a** and **17b**–**d** can be considered as the most effective hybrids among the tested ones, these aryltriazolyl derivatives were selected for the dose–response curve determination and for the colony formation assays. It must be noted here that **10a**, which is a fluorinated compound, was identified as one the most potent antiproliferative agents in the recent series of our imipridone hybrids, which also include other mono- and difluoro-substituted derivatives (**9a**,**b**, **10b** and **16a**,**b**). Besides the conjugation with ferrocene-containing fragments, the introduction of fluoro substituents was considered to be an attractive strategy to attenuate the antiproliferative activity of the targeted hybrids. In terms of decreasing the efficient dose, we identified highly active imipridones, which were fluorinated on the *meta* position(s) of the benzyl group that was attached to the *N*-7 skeletal position, that feature IC_50_ values in the low nanomolar range on different human malignant cell lines [31]. On the other hand, the improved drug-like properties of the fluorinated compounds can be ascribed to the significant influence of fluorine on the polarity, acidity, conformation, membrane permeability and, thus, on the pharmacokinetics of the potential drug candidates [32]. Moreover, in the context of the possible prospects of our research on ferrocene-containing hybrids, it must be mentioned here that fluorination has also been successfully explored in labeling studies that were aimed at improving the bioanalytical sensitivity in drug uptake studies on organometallic anticancer agents [33].

### 2.4. Dose–Response Curves of Selected Imipridone Hybrids

**Figure 2:**
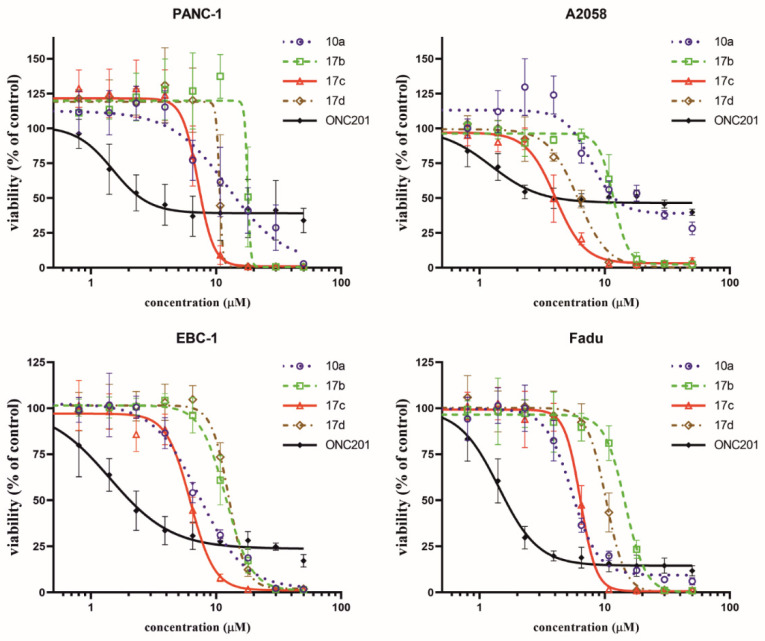
Dose–response curves of selected imipridone hybrids (**10a**, **17b**, **17c** and **17d**) and reference compound, ONC201, on different cancer cell lines based on MTT cell viability assay. Curves were fitted by GraphPad Prism 8 software using nonlinear regression (variable slope; 95% CI; *n* = 4).

As detailed above, the most effective compounds, **10a** and **17b**–**d,** were selected for the determination of their dose–response curves and IC_50_ values on previously examined cancer cell lines (PANC-1, A2058, EBC-1 or Fadu) by an MTT cell viability assay. ONC201 was applied as a reference compound. The dose–response curves are shown in Figure 2. The IC_50_ values are presented in Table 2.

Although the novel imipridone hybrids (**10a** and **17b**–**d**) resulted in higher IC_50_ values than the reference imipridone, ONC201, the dose–response curves reveal that each of the tested cancer cell lines contained a significant amount of viable cells after 72 h of ONC201 treatment. On the contrary, the novel imipridone hybrids, **17b**, **17c** and **17d,** were able to reduce the cell viability to near zero on every tested cancer cell line.

### 2.5. Colony Formation Assay

The cell viability screening and the dose–response curves revealed that PANC-1 and A2058 exhibit the least susceptibility to ONC201 treatment. Accordingly, previously published data [3] confirm that PANC-1 can be categorized as a cell line that is resistant to ONC201 (and ONC212) imipridone derivatives. On the basis of these findings, the PANC-1 cell line was selected for the colony formation assay, and the cells were treated with compounds at a 10 µM concentration for 24 and 72 h, followed by 5 and 3 days of postincubation periods.

**Figure 3:**
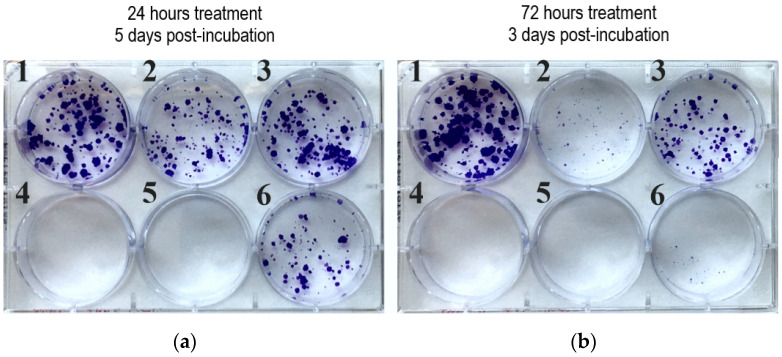
Colony formation assay of selected imipridone hybrids on PANC-1 cell line at 10 µM concentration: (**a**) 24 h treatment followed by 5 days postincubation; (**b**) 72 h treatment followed by 3 days postincubation. 1: control; 2: **10a**; 3: **17b**; 4: **17c**; 5: **17d**; 6: **ONC201**.

The results of the colony formation assay are presented in Figure 3, and they clearly demonstrate that, even after 72 h of exposure time with ONC201 (Figure 3b), there were still viable PANC-1 cells that were able to form colonies. Treatment with Compound **10a** led to a similar result. However, in the cases of Compounds **17c** and **17d**, the PANC-1 cells were completely eradicated, even after 24 h of exposure time (Figure 3a). In accordance with the IC_50_ data that were gained by the MTT test, 10 µM proved to be insufficient for **17b** to eradicate PANC-1 cells. On the basis of these results, **17c** and **17d** proved to be the most potent imipridone derivatives, and they bear the capacity to overcome the resistance to ONC201 treatment.

### 2.6. Cytotoxicity Assay with the Most Potent Hybrids on Cancer Cell Lines and Nontumorous Fibroblast Cells

**Figure 4:**
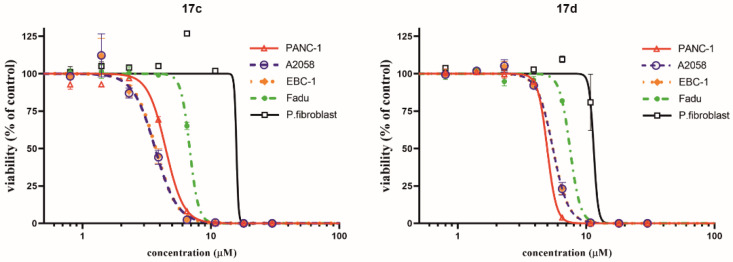
Dose–response curves of the two most potent imipridone hybrids, **17c** and **17d,** on different cancer cell lines, and on nontumorous primary fibroblast cells, after 72 h of treatment. Cell viability was determined by CellTiter-Glo assay. Curves were fitted by GraphPad Prism 8 software using nonlinear regression (variable slope; *n* = 3).

The imipridone hybrids, **17c** and **17d**, proved to be the most potent antiproliferative agents in the dose–response and colony formation assays. However, it is critical, from the aspect of their therapeutic potential, to assess their toxicity in nontumorous cells. To this end, concentration-dependent viability studies were performed with these two compounds on the four investigated tumor cell lines and on primary fibroblast cells by using CellTiter, which is a method that is more sophisticated than MTT analysis. The obtained dose–response curves (Figure 4) unambiguously reveal that the ferrocenyltriazole analogue, **17c**, can be considered to offer substantial therapeutic windows, as it led to the practically complete eradication of all the investigated tumor cells at a 10 µM concentration, while it displayed no toxicity on the fibroblast cells at the same concentration. The IC_50_ values that were obtained from these of experiments (Table 3) further support our view about the promising therapeutic potential of **17c**. The dose–response curves and the IC_50_ data that were obtained from the experiments with the phenyltriazolyl counterpart, **17d**, which were carried out under the same conditions, are indicative of significantly narrower therapeutic windows with regard to this compound. Nevertheless, at a 10 µM concentration, **17d** also completely eradicates PANC-1 cells, without showing toxicity on the fibroblast cells.

### 2.7. Apoptosis and Necrosis Quantitation Assay

**Figure 5:**
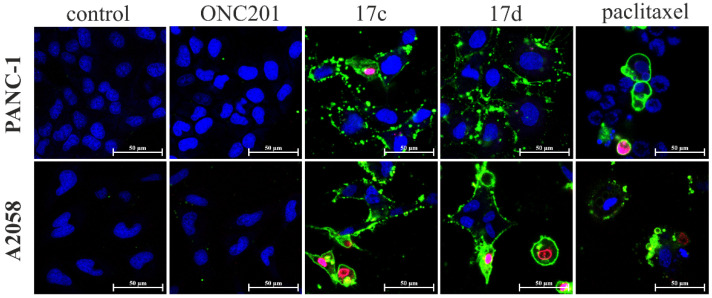
Apoptosis–necrosis detection assay on PANC-1 and A2058 cancer cells by confocal laser scanning microscopy. Cells were treated with ONC201, **17c** and **17d** for 24 h at 10 µM. Paclitaxel was used as a positive control at 1 µM, and untreated cells as a negative control. Annexin V labeled with CF488A stains apoptotic cells with green fluorescence by binding to phosphatidylserine exposed on the cell surface. Ethidium Homodimer III is a nucleic acid probe that is impermeant to live cells and early apoptotic cells, but that stains necrotic cells and late apoptotic cells with red fluorescence.

In order to gain preliminary information about the mechanism of action of the most potent imipridone hybrids, **17c** and **17d**, we performed an apoptosis–necrosis detection assay on PANC-1 and A2058 cells by using ONC201 as the structurally related reference, paclitaxel as the positive control and untreated cells as the negative control (Figure 5). The experiments demonstrated a marked apoptosis-inducing effect of both the selected novel imipridone hybrids on both cell lines, while no apoptotic/necrotic signal was observed after 24 h of treatment with ONC201. These findings indicate differences in the kinetics, and possibly the targets, of the proapoptotic/pronecrotic effects of **17c** and **17d**, and those identified for ONC201.

## 3. Materials and Methods

All fine chemicals were obtained from commercially available sources (Merck, Budapest, Hungary), Fluorochem (Headfield, UK), Molar Chemicals (Budapest, Hungary), VWR (Budapest, Hungary) and were used without further purification. THF was distilled from LiAlH_4_. Merck Kieselgel (230–400 mesh, 60 Å) was used for the flash column chromatography. The ^1^H- and ^13^C-NMR spectra were recorded in CDCl_3_ solution in 5 mm tubes at room temperature on a Bruker DRX-500 spectrometer (Bruker Biospin, Karlsruhe, Baden Württemberg, Germany), at 500 (^1^H) and 125 (^13^C) MHz, with the deuterium signal of the solvent as the lock, and TMS as the internal standard (^1^H, ^13^C). The 2D-COSY, HSQC, and HMBC spectra were obtained by using the standard Bruker pulse programs. The ^1^H- and ^13^C-NMR signals listed in the Supplementary Materials (S5) are unambiguously confirmed by the 2D-COSY, HSQC and HMBC measurements. The exact mass measurements were performed by using a Q Exactive Focus Orbitrap instrument (Thermo Fisher Scientific, Bremen, Germany) that was equipped with a heated electrospray ionization source. High-performance liquid chromatograms were acquired on a Jasco HPLC system (ABL&E-JASCO Hungary Ltd., Budapest, Hungary), using an Avantor ACE C18-PFP (100/4.00 mm; 3 μm; 100 Å; VWR) column. The HPLC column, Acetonitrile (cat. no.: 83639.320) and TFA (cat. no.: 153112E) were obtained for VWR. Data were analyzed by ChromPass 1.8 software. Samples were dissolved in acetonitrile (injected volume: 5–10 µL). Linear gradient elution (0 min: 15% B; 7 min: 100% B; 11 min) of Eluent A (0.1% TFA in water) with Eluent B (acetonitrile/water 9:1 mixture complemented with 0.1% TFA) was used. (Flow rate: 0.8 mL/min; detection: 254 nm).

### 3.1. Synthetic Procedures

#### 3.1.1. General Procedure for the Synthesis of N-Substituted Methylcarboxylate Piperidones (**3a**–**e**)

Methyl acrylate (15 mmol, 5 eq.) was added to a solution of a primary amine (**1a**–**e**) (3 mmol, 1 eq.) in methanol (5 mL), and the mixture was stirred at room temperature for 48 h. The solution was concentrated in vacuo to obtain the crude diester product (**2a**–**e**). Without further purification, it was dissolved in dry THF (8 mL), and NaH (15 mmol, 5 eq.) was added gradually at 0 °C while stirring the mixture. The suspension was brought to room temperature. After stirring for an additional 2 h, the suspension was poured over ice (100 g), and the pH was set to 7 with acetic acid. The aqueous phase was extracted 3 times with ethyl acetate (40 mL). The combined organic phases were dried over Na_2_SO_4_ and were concentrated in vacuo, obtaining the *N*-substituted methylcarboxylate piperidone product (**3a**–**e**), which was used without further purification.

#### 3.1.2. Synthesis of 2-(Methylthio)-4,5-dihydro-1*H*-imidazole-1-carboxylate (**5**)

2-Methylthio-4,5-dihydroimidazolium iodide (50 g, 0.205 mol, 1 eq.) and triethylamine (70 mL, 50.8 g, 0.502 mol, 2.45 eq.) were dissolved in DCM (250 mL). The solution was cooled to 0 °C and methylchloroformate (22 mL, 27 g, 0.28 mol, 1.4 eq.) was added dropwise. The reaction mixture was allowed to warm up to room temperature and was stirred overnight. The mixture was concentrated in vacuo, EtOAc (400 mL) was added and the suspension was stirred for 30 min. The precipitated ammonium salts were filtered off and washed with EtOAc (2 × 50 mL). The combined solution was evaporated to dryness. The solid residue was triturated with water (200 mL), filtered off and dried under vacuum to obtain **5** as a white solid. (Yield: 27.2 g (76%); Rf: 0.60 (DCM:MeOH = 25:1 + TEA; silicagel)).

#### 3.1.3. General Procedure for the Synthesis of Cyclic Guanidines (**6e**–**g**)

Primary amines (**1e**–**g**) (10 mmol, 1 eq.) were dissolved in a mixture of methanol (24 mL) and acetic acid (6 mL). To the solutions, 2-(methylthio)-4,5-dihydro-1*H*-imidazole-1-carboxylate (12.5 mmol, 1.25 eq.) was added, and the resulting mixtures were stirred at reflux for 18 h. The solutions were concentrated in vacuo, and the oily residues were dissolved in DCM (100 mL) and were washed twice with a 3 M NaOH solution (10 mL), and once with brine (10 mL). The organic phases were dried over Na_2_SO_4_ and were concentrated in vacuo. The products were used without further purification.

#### 3.1.4. General Procedure for the Synthesis of Imipridones (**7ag**, **7be**, **7bf**, **7ce**, **7cf**, **7df**, **7ef** and **7eg**)

*N*-substituted methylcarboxylate piperidones (**3a**–**e**) (2 mmol, 1 eq.) and cyclic guanidines (**6e**–**g**) (2 mmol, 1 eq.) were dissolved in methanol (10 cm^3^) and a 3.6 M NaOMe solution in methanol was added (2.5 mmol, 1.25 eq.). The mixtures were stirred at reflux for 12 h. In the case of reactions with 6f, the mixture was cooled after 12 h, and the precipitated white crystalline product (**7bf**, **7cf**, **7df** and **7ef**) was filtered off, washed twice with cold methanol (2 × 1 mL) and dried in vacuo. In other cases, the mixtures were cooled, concentrated in vacuo, dissolved in DCM (100 mL), washed with water (2 × 10 mL) and brine (10 mL), dried over Na_2_SO_4,_ and concentrated in vacuo. The crude yellow oily products were purified with column chromatography (silicagel, DCM:MeOH:NH_3_ = 15:1:0.1) and crystallized from Et_2_O as yellowish white powders (**7ag**, **7be**, **7ce** and **7eg**).

#### 3.1.5. General Procedure for the Synthesis of Azido Imipridones (**7bh**, **7ch**, **7hf** and **7hg**)

Imipridones with a *p*-NHBoc substituent (**7be**, **7ce**, **7ef** and **7eg**) (1 mmol, 1 eq.) were dissolved in cc. HCl (5 mL) and were stirred for 30 min with intensive gas evolution at the beginning. After 30 min, the solution was cooled to 0 °C and aqueous NaNO_2_ solution (2 mmol, 2 eq. in 1.5 mL water) was added dropwise using a syringe. The mixtures were stirred at 0 °C for 45 min. Solid NaN_3_ (10 mmol, 10 eq.) was added in portions over the course of 15 min. The mixtures were stirred for 30 min at 0 °C, then were allowed to warm up to room temperature and were stirred for an additional 1 h. The acid was neutralized with Na_2_CO_3_ and the mixtures were extracted with DCM (3 × 40 mL). The combined organic phases were washed with water (2 × 10 mL) and brine (10 mL), dried over Na_2_SO_4_ and concentrated in vacuo. The products were crystallized from Et_2_O as a yellowish-white solid (**7bh**, **7ch**, **7hf** and **7hg**).

#### 3.1.6. General Procedure for Copper(I) Catalysed 1,4-Azide-alkyne Cycloadditions

Azido imipridones (**7bh**, **7ch**, **7hf** and **7hg**) (0.5 mmol, 1 eq.), alkyne compounds (**8**, **11** and **12**) (0.5 mmol, 1 eq.) and CuI (0.5 mmol, 1 eq.) were dissolved in DMSO (2 mL) and were stirred for 12 h at room temperature in a closed vial. After 12 h, the mixture was poured over water (40 mL) and was stirred for 20 min. The precipitate was filtered off and washed with water (5 × 10 mL). The filter was washed with DCM (4 × 10 mL). The combined filtrate was dried over Na_2_SO_4_ and concentrated in vacuo. The crude product was purified using column chromatography (silicagel, DCM:MeOH:NH_3_ = 15:1:0.1) and was crystallized from Et_2_O to yield an orange (in the case of ferrocene-containing compounds—**10a,b**, **12a**,**b**) or white powder (**13**, **14**_protected_).

#### 3.1.7. General Procedure for Ruthenium(II) Catalysed 1,5-Azide-alkyne Cycloadditions

Azido imipridones (**7bh**, **7ch** and **7hf**) (0.5 mmol, 1 eq.), ethynyl-ferrocene (**8**) (0.5 mmol, 1 eq.) and Cp*RuCl(PPh_3_)_2_ (0.025 mmol, 0.05 eq.) were dissolved in DMF (2 mL) and stirred for 12 h at room temperature. After 12 h, the mixture was poured over water (40 mL) and stirred for 20 min. The precipitate was filtered off and washed with water (5 × 10 mL). The filter was washed with DCM (4 × 30 mL). The combined filtrate was dried over Na_2_SO_4_ and concentrated in vacuo. The crude product was purified using column chromatography (Al_2_O_3_, DCM) and was crystallized from Et_2_O to yield an orange powder (**9a**,**b**, **11**).

#### 3.1.8. General Procedure Used for the Sonogashira Coupling

Iodo derivatives (**7bf**, **7cf**, **11**, **12a**, **13** and **14**) (0.2 mmol, 1 eq.), alkyne compounds (**8**, **12** and **15**) (0.2 mmol, 1 eq.), CuI (0.04 mmol, 0.2 eq.), Pd(PPh_3_)_2_Cl_2_ (0.02 mmol, 0.1 eq.) and DIPEA (1 mmol, 5 eq.) were dissolved in DMF (2 mL) and stirred for 24 h. After 24 h, the mixture was poured over water (30 mL) and stirred for 20 min. The precipitate was filtered off and washed with water (5 × 10 mL). The filter was washed with DCM (4 × 20 mL). The combined filtrate was dried over Na_2_SO_4_ and concentrated in vacuo. The crude product was purified using column chromatography (silicagel, DCM:MeOH:NH_3_ = 15:1:0.1), and was crystallized from Et_2_O to yield an orange (in the case of ferrocene-containing compounds—**16a**–**d** and **17b,c**) or white powder (**17a**, **17d,e** and **18**_protected_).

#### 3.1.9. General Procedure for the Removal of Trimethylsilyl-Protecting Group

The trimethylsilyl-protected alkynes (**14**_protected,_ **18**_protected_) (0.1 mmol, 1 eq.) were dissolved in THF (5 mL), and TBAF (0.5 mmol, 5 eq.) was added. The mixture was stirred for 1.5 h at room temperature and poured over water (50 mL). The suspension was extracted with DCM (4 × 15 mL). The combined organic phase was dried over Na_2_SO_4_ and concentrated in vacuo. The product was crystallized from Et_2_O to yield a white powder (**14**, **18**).

A detailed spectral characterization with the assigned ^1^H- and ^13^C-NMR data and HRMS of the targeted compounds that were screened in the biological assays can be found in the Supplementary Material (S1).

### 3.2. Cell Culturing

PANC-1 (human pancreatic carcinoma) cells and Fadu (human hypopharyngeal carcinoma) cells were obtained from the American Type Culture Collection (ATCC, Rockville, MD, USA). PANC-1 and Fadu cells were cultured in Dulbecco’s modified Eagle’s medium (DMEM, Thermo Fisher Scientific, cat. no.: 41965039), supplemented with 10% FBS (ATCC, cat. no.: 30-2020). EBC-1 (human lung squamous cell carcinoma) cells were obtained from the Japanese Collection of Research Bioresources Cell Bank (JCRB, Ibaraki, Osaka, Japan) and were cultured in Eagle’s minimum essential medium (EMEM, Lonza, Basel, Switzerland, cat. no.: BE12-611F), supplemented with 10% FBS. The A2058 (human melanoma) cells were obtained from the European Collection of Authenticated Cell Cultures (ECACC, Salisbury, UK) and were cultured in Roswell Park Memorial Institute Medium (RPMI-1640, Thermo Fisher Scientific, cat. no.: 11875-093), supplemented with 10% FBS. Primary fibroblast culture was prepared from human skin biopsies. Fibroblasts were cultured in Dulbecco’s modified Eagle’s medium (DMEM) (Thermo Fisher Scientific, cat. no.: 41965039), supplemented with 10% FBS (Thermo Fisher Scientific), 1% penicillin–streptomycin (Thermo Fisher Scientific, cat. no.: 15070063) and 1% MEM Non-Essential Amino Acids Solution (Thermo Fisher Scientific, cat. no.: 11140035). Cell cultures were maintained in a humidified incubator (Heracell VIOS 160) at 37 °C under a 5% CO_2_ atmosphere.

### 3.3. MTT Cell Viability Assay

The effects of the synthesized compounds on the cell viability were measured via an MTT cell viability assay (Sigma-Aldrich, Inc., St Louis, MN, USA). Cells were seeded in a transparent 96-well cell culture plate (VWR) with the following density: PANC-1: 750 cells/well; Fadu: 1000 cells/well; and A2058 and EBC-1: 1500 cells/well. After seeding, cells were incubated further for 48 h before the treatment. Cells were treated with the compounds for 72 h. Three concentrations (25.0 µM; 8.3 µM; 2.8 µM) were applied at the cell-viability screening, while, at the determination of the dose–response curves, 1.67-fold serial-diluted compound concentrations were applied (range: 50–0.8 µM). After the treatment, the treating mediums were removed, and a 50 µL/well MTT solution (1 mg/mL) was added. Cells were incubated in a CO_2_ incubator for 2 h. The medium was removed, and the formazan crystals that were formed by the cells were dissolved by using 1% 0.1 M HCl and 10% Triton X-100 (Merck) containing propan-2-ol. The absorbance was read at 570 nm, using 635 nm as a reference wavelength, on a BioTek Synergy 2 Multi-Mode Reader (BioTek, Winooski, VT, USA). Untreated cells (in the corresponding DMSO-containing medium) were used as the control. Data were evaluated by MS Excel 2016. Dose–response curves and IC_50_ values were determined by GraphPad Prism 8 software (nonlinear regression; variable slope). Results are derived from at least two independent experiments, and each experiment was performed in duplicates (*n* = 4).

### 3.4. CellTiter-Glo Cell Viability Assay

Effects of the two most potent compounds, 17c and 17d, on the viability of PANC-1, A2058, Fadu and EBC-1 cancer cells and normal fibroblast cells was measured via the CellTiter-Glo^®^ luminescent cell viability assay (Promega, Madison, WI, USA), according to the manufacturer’s instructions. Cells were plated onto a flat-bottomed white 96-well plate (BRANDplates, cat. no.: 781965) and were treated with the same method described for the MTT cell viability assay. After the treatment, the luminescence signal was recorded using a microplate reader (BioTek Synergy 2 Multi-Mode Reader, BioTek, Winooski, VT, USA). Dose–response curves (using a nonlinear regression model) were generated, and IC_50_ values were determined using Graph Pad Prism 8 software (GraphPad Software, San Diego, CA, USA).

### 3.5. Colony Formation Assay

The long-term cell survival after treatments was determined by clonogenic assay. PANC-1 cells were seeded in a transparent 6-well cell culture plate (VWR) (density: 750 cells/well). After seeding, cells were incubated for 72 h before the treatment. Cells were treated with the compounds at 10 µM for 24 or 72 h. Then, the medium was removed, and the cells were washed with phosphate-buffered saline (PBS). The cells formerly treated for 24 or 72 h were further incubated in their corresponding medium for 5 or 3 days, respectively. Thereafter, the medium was removed, and the cells were washed with PBS and were fixed with 4% paraformaldehyde for 10 min. After fixation, cells were washed with PBS, and 0.5% *w/v* crystal violet (CV) solution was added. After 1 h, the CV solution was removed, and cells were washed thoroughly with water. Images were created by Corel Photo-Paint 2019.

### 3.6. Apoptosis and Necrosis Quantitation Assay

PANC-1 and A2058 cells were seeded in eight-well Ibidi^®^ μ-Slide microscopic slides (5 × 10^3^ cells/well) and were allowed to adhere for 48 h. Cells were then treated with 10 µM of ONC201, **17c** and **17d**, and 1 µM of paclitaxel in cell culture medium supplemented with 10% FBS and were incubated in a humidified 5% CO_2_ atmosphere incubator for 24 h at 37 °C. Apoptosis and Necrosis Quantitation Kit Plus (Biotium, cat. no.: 30065) was used, according to the manufacturer’s instructions. For the nuclear staining of live cells, DRAQ5 was used (5 µM, 30 min). Images of cells were acquired with a confocal laser microscope (Zeiss Confocal LSM 710, Carl Zeiss AG, Oberkochen, Germany). (Objective: Plan-Apochromat 63×/1.40 Oil DIC M27. Pinhole: 4.04 AU. Laser wavelengths: 488 nm and 633 nm. Detection wavelengths: 504–536 nm; 602–631 nm; 692–758 nm).

## 4. Conclusions

Drug resistance that develops in the highly adaptive cancer cells is a major factor that hampers the clinical efficacy of anticancer chemotherapy. The prosurvival mechanisms that are upregulated in some tumor cells were also found to limit the anticancer activity of ONC201, which is the first-in-class imipridone. To address the problem that is associated with the resistance of cancer cells to imipridone treatment, we synthesized a set of novel ferrocene-containing hybrids with potential ROS-generating capabilities to attenuate the antiproliferative activity exerted by the imipridone core. This strategy of fragment-based design is in line with the concept on the hybrid compounds that emerged as prominent multitargeted anticancer agents that contain the inducers of two or more cytotoxic mechanisms [34].

The initial screenings revealed that four newly synthesized imipridone hybrids (**10a**, **17b**, **17c** and **17d**) were significantly more effective at decreasing the cell viability compared to ONC201, on all four examined cell lines (PANC-1, A2058, EBC-1 and Fadu). Although the IC_50_ values of these compounds were found to be higher than those of ONC201, the colony formation assays performed on the most treatment-resistant PANC-1 cell line [3] proved that two of the emergent hybrids (**17c** and **17d**) eliminated the resistance, and no viable cells could be observed after treatments at 10 µM concentrations.

The SAR discussed above strongly suggest that—in a cooperative manner—a 4/5-aryl-1*H*-1,2,3-triazol-1-yl residue (aryl = phenyl, ferrocenyl or others not investigated here) and the protruding basic 3-aminopropynyl substituent placed at the *para* positions of the benzyl substituents that pend on the distant imipridone skeletal positions, 7 and 4, respectively, are able to overcome the resistance of cancer cells, for which the assays are evaluated in this research.

However, the dose–response curves show that the cell viability drops very rapidly with the change in the concentration of the presented hybrids, so that even the two most potent compounds should be applied at relatively high concentrations within a very narrow window to evade cell resistance, which might limit their applicability. On the other hand, by highlighting the beneficial properties of **17c** and **17d**, it was also demonstrated that, in shorter treatments (24 h), these compounds are far more efficient in apoptosis/necrosis induction in PANC-1 and A2058 cells than ONC201, which caused no detectable apoptotic/necrotic signal after an identical period of treatment. Finally, in comparative cytotoxicity studies on PANC-1 and A2058 cells and on nontumorous primary fibroblasts, the organometallic derivative, **17c,** emerged as the most potent anticancer agent in the presented research. Without exerting an observable toxicity on the fibroblasts at 10 μM, this lead compound was capable of selectively eradicating the tumor cells at the aforementioned concentration. The results of all of the comparative experiments suggest that the advantageous properties of the two most potent compounds, with particular regard to overcoming resistance, might probably be associated with their multitarget character, which warrants the activation of at least a dual mechanism of action.

In summary, despite the fact that the IC_50_ values that were produced by our most potent hybrids are 5–10 times higher than those of ONC201 on the same cell lines, the overall results are very promising with regard to **17c** and **17d** as potential anticancer drug candidates. We believe this work is worth continuing by fine-tuning the structures of the aforementioned most potent hybrids with the variation and reasonable modification of the substitution pattern on the imipridone core, and by exploring the molecular fragments introduced so far and their structurally related versions. The cytotoxicity of **17c**,**d**, and their modified versions, are to be assessed on a significantly extended platform of cell lines to evaluate the effects of diverse functionalizations on the aromatic rings and on the amino group, as well as further versions of the hybridization on the triazole ring with other pharmacophores.

## Figures and Tables

**Table 1:** Cell viability screening on PANC-1, A2058, EBC-1 and Fadu cell lines. (Data represent the mean ± SD; *n* = 4). Cell viability data below 10% and in between 10 and 20% are presented in bold and in italics, respectively.

Compound Identifier and Structure		Cell Viability (% of Control) ± SD (*n* = 4)				
		µM	PANC-1	A2058	EBC-1	Fadu
**ONC 201**	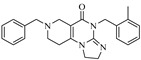	**25**	54 ± 23	47 ± 4	31 ± 3	*16 ± 2*
		**8.3**	63 ± 33	52 ± 3	34 ± 4	21 ± 1
		**2.8**	62 ± 25	59 ± 6	37 ± 3	25 ± 4
**9a**	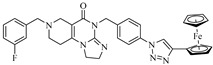	**25**	88 ± 5	82 ± 12	62 ± 15	69 ± 21
		**8.3**	94 ± 10	90 ± 14	66 ± 8	74 ± 6
		**2.8**	114 ± 25	88 ± 7	75 ± 7	79 ± 7
**9b**	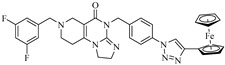	**25**	109 ± 7	94 ± 9	65 ± 13	72 ± 6
		**8.3**	126 ± 12	103 ± 15	79 ± 13	71 ± 13
		**2.8**	122 ± 20	104 ± 13	88 ± 9	74 ± 8
**10a**	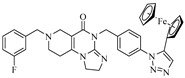	**25**	13 ± 3	26 ± 2	**3 ± 1**	**9 ± 2**
		**8.3**	48 ± 1	54 ± 3	34 ± 4	*19 ± 5*
		**2.8**	101 ± 9	114 ± 5	96 ± 9	79 ± 2
**10b**	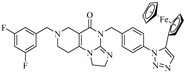	**25**	77 ± 18	60 ± 6	21 ± 6	29 ± 11
		**8.3**	93 ± 7	83 ± 8	67 ± 7	66 ± 28
		**2.8**	117 ± 12	120 ± 8	104 ± 9	82 ± 16
**16a**	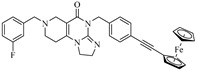	**25**	124 ± 46	77 ± 5	58 ± 3	59 ± 2
		**8.3**	120 ± 42	90 ± 13	63 ± 14	75 ± 8
		**2.8**	126 ± 49	87 ± 10	76 ± 4	85 ± 14
**16b**	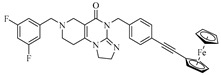	**25**	103 ± 17	80 ± 5	68 ± 12	72 ± 6
		**8.3**	123 ± 34	89 ± 3	69 ± 13	84 ± 13
		**2.8**	126 ± 31	94 ± 5	80 ± 9	79 ± 4
**16c**	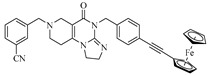	**25**	83 ± 9	77 ± 10	61 ± 18	58 ± 24
		**8.3**	116 ± 25	80 ± 7	70 ± 13	79 ± 13
		**2.8**	123 ± 35	91 ± 16	81 ± 6	78 ± 9
**17a**	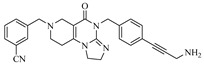	**25**	38 ± 32	82 ± 4	77 ± 4	36 ± 36
		**8.3**	73 ± 16	96 ± 3	101 ± 7	52 ± 25
		**2.8**	97 ± 13	92 ± 8	94 ± 6	88 ± 10
**18**	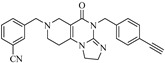	**25**	50 ± 26	44 ± 4	35 ± 2	*14 ± 2*
		**8.3**	50 ± 22	50 ± 4	36 ± 5	*17 ± 2*
		**2.8**	52 ± 26	50 ± 6	35 ± 12	16 ± 1
**11**	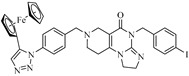	**25**	95 ± 5	58 ± 22	27 ± 19	51 ± 27
		**8.3**	126 ± 27	65 ± 3	52 ± 12	65 ± 26
		**2.8**	126 ± 13	84 ± 10	86 ± 7	81 ± 13
**12a**	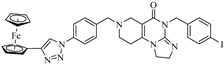	**25**	91 ± 12	86 ± 9	58 ± 15	55 ± 17
		**8.3**	99 ± 12	87 ± 6	69 ± 17	64 ± 26
		**2.8**	100 ± 9	91 ± 5	79 ± 17	72 ± 19
**12b**	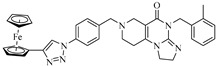	**25**	91 ± 1	89 ± 3	66 ± 4	89 ± 1
		**8.3**	108 ± 5	82 ± 2	73 ± 1	93 ± 4
		**2.8**	109 ± 9	79 ± 6	84 ± 7	98 ± 3
**13**	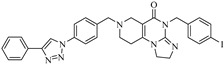	**25**	82 ± 21	85 ± 4	76 ± 4	75 ± 14
		**8.3**	104 ± 1	79 ± 7	82 ± 6	81 ± 14
		**2.8**	88 ± 23	73 ± 6	88 ± 19	90 ± 4
**17b**	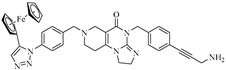	**25**	**1 ± 1**	**3 ± 1**	**3 ± 3**	**3 ± 3**
		**8.3**	98 ± 16	74 ± 3	60 ± 31	73 ± 21
		**2.8**	95 ± 4	83 ± 8	97 ± 8	92 ± 10
**17c**	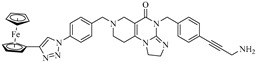	**25**	**1 ± 1**	**2 ± 1**	**3 ± 2**	**2 ± 2**
		**8.3**	23 ± 8	**7 ± 3**	*12 ± 10*	22 ± 24
		**2.8**	101 ± 8	79 ± 9	83 ± 13	63 ± 32
**17d**	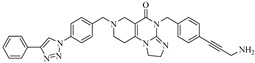	**25**	**1 ± 1**	**3 ± 1**	**3 ± 1**	**3 ± 3**
		**8.3**	96 ± 20	45 ± 9	63 ± 14	51 ± 49
		**2.8**	102 ± 19	85 ± 7	107 ± 13	87 ± 9
**17e**	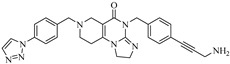	**25**	91 ± 10	76 ± 2	86 ± 12	58 ± 20
		**8.3**	103 ± 13	89 ± 4	111 ± 17	89 ± 17
		**2.8**	92 ± 11	93 ± 6	108 ± 17	100 ± 7
**16d**	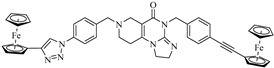	**25**	77 ± 20	76 ± 4	53 ± 11	53 ± 4
		**8.3**	85 ± 21	81 ± 9	70 ± 17	71 ± 19
		**2.8**	78 ± 10	91 ± 17	80 ± 18	72 ± 17

**Table 2:** IC_50_ values (µM) of selected imipridone hybrids (**10a**, **17b**, **17c** and **17d**) and reference compound, ONC201, after 72 h of treatment, based on MTT assay. IC_50_ values were determined by GraphPad Prism 8 software (nonlinear regression; variable slope; best-fit values; *n* = 4).

Treated Cells	10a	17b	17c	17d	ONC201
PANC-1	12.4	17.8	7.2	10.6	1.5
A2058	7.8	11.8	4.0	6.1	1.2
EBC-1	7.3	12.0	6.2	12.6	1.4
Fadu	5.5	14.6	6.4	10.3	1.4

**Table 3:** IC_50_ values (µM) of the two most potent imipridone hybrids, **17c** and **17d,** on cancer cells and on nontumorous primary fibroblast cells after 72 h of treatment, based on CellTiter-Glo cell viability assay. IC_50_ values were determined by GraphPad Prism 8 software (nonlinear regression; variable slope; best-fit values; *n* = 3).

Treated Cells	17c	17d
PANC-1	4.4	5.0
A2058	3.7	5.5
EBC-1	3.7	5.5
Fadu	6.8	7.5
Primary fibroblast	15.8	11.4

## Data Availability

The data generated and analyzed during our research are not available in any public database or repository but will be shared by the corresponding author upon reasonable request.

## Supplementary Materials

The following are available online at www.mdpi.com/article/10.3390/ph15040468/s1, S1: HPLC chromatograms of selected compounds; S2: ^1^H-, ^13^C NMR and HRMS data of the targeted compounds; S3: Copies of the ^1^H- and ^13^C-NMR spectra; S4: Copies of the HRMS spectra; S5: Data of MTT Cell Viability Assay; S6: Data of CellTiter-Glo Cell Viability Assay.
