# Supplementary material for: Synthesis and Antiproliferative Activity of Novel Imipridone–Ferrocene Hybrids with Triazole and Alkyne Linkers
Source: Pharmaceuticals (Basel). 2022 Apr 12;15(4):468. doi: 10.3390/ph15040468 (PMC9028308; doi:10.3390/ph15040468)
Supplement: Supplementary file 1 [file pharmaceuticals-15-00468-s001.zip › pharmaceuticals-1651318-supplementary.pdf]

Supplementary Materials

Synthesis and antiproliferative activity of novel imipridone-ferrocene hybrids with triazole and alkyne linkers

Tamás Czuczi [1], József Murányi [2], Péter Bárány [1] István Móra [2], Adina Borbély [3], Miklós Csala [2,4] and Antal Csámpai [1,*]

[1] Department of Organic Chemistry, Eötvös Loránd University (ELTE) Budapest Pázmány P. sétány 1/A, H-1117, Hungary; czuczi.tamas@gmail.com; (T.C.), peterbarany@ceasar.elte.hu; (P.B.), csampai@caesar.elte.hu (A.Cs.)
[2] MTA-SE Pathobiochemistry Research Group, Tűzoltó u. 37-47, H1094 Budapest, Hungary; (jozsefmuranyi84@gmail.com. (J.M.) istvan.mora1313@gmail.com. (I.M.) and csala.miklos@med.semmelweis-univ.hu.; (M.Cs.)
[3] MTA-ELTE Lendület Ion Mobility Mass Spectrometry Research Group and Department of Analytical Chemistry, Eötvös Loránd University (ELTE) Budapest Pázmány P. sétány 1/A, H-1117, Hungary; adina.borbely@ttk.elte.hu.; (A.B.)
[4] Department of Molecular Biology, Semmelweis University H1094 Budapest, Hungary (M.Cs.)
* Correspondence: csampai@caesar.elte.hu; Tel.: (+36 1 372 2500/6591)

Content:

S.1. HPLC chromatograms of selected compounds pp. 2 – 6

S.2. ^{1}H-, ^{13}C NMR and HRMS data of the targeted compounds pp. 7 – 14

S.3. Copies of the ^{1}H- and ^{13}C-NMR spectra pp. 15 – 36

S.4. Copies of the HRMS spectra pp. 37 – 48

S.5. Data of MTT Cell Viability Assay pp. 49 – 51

S.6. Data of CellTiter-Glo Cell Viability Assay p. 52

S.1. HPLC chromatogram of selected compounds

ONC201

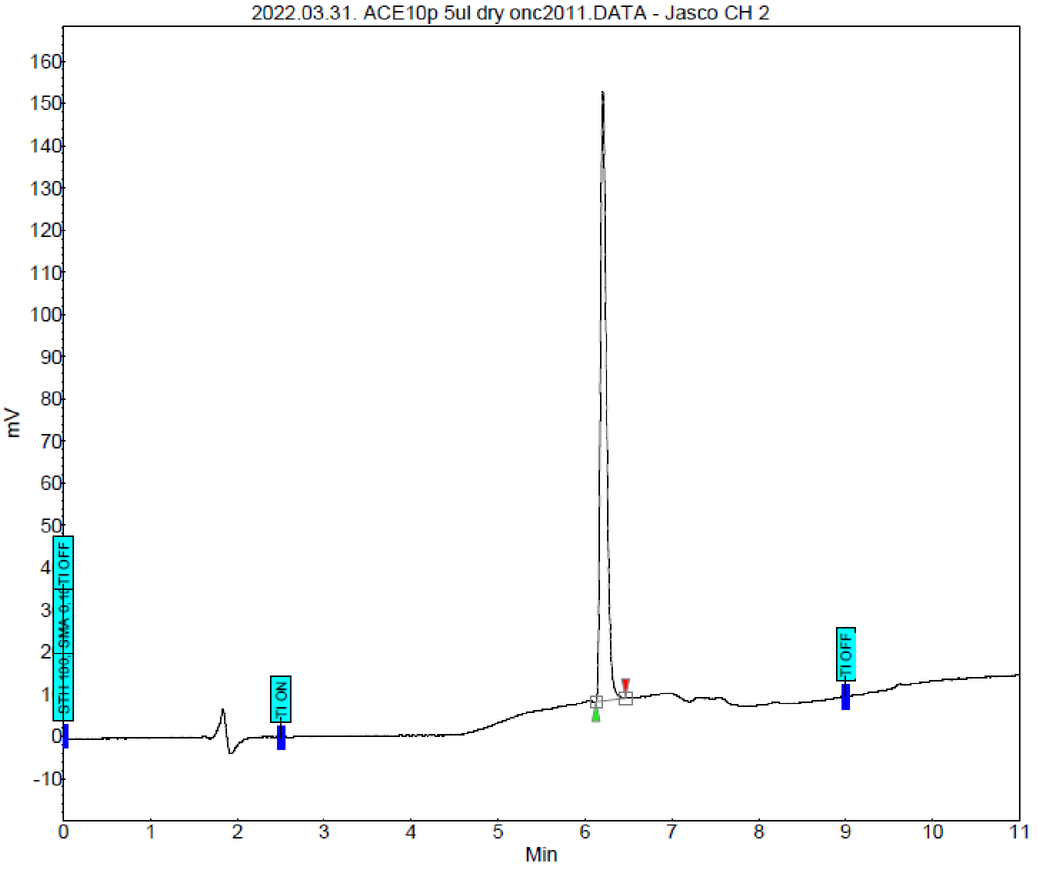

Index	Name	Time [Min]	Quantity [% Area]	Height [mV]	Area [mV.Min]	Area % [%]
1	UNKNOWN	6,207	100,00	144,3	11,541	100,000
Total			100,00	144,3	11,541	100,000

10a

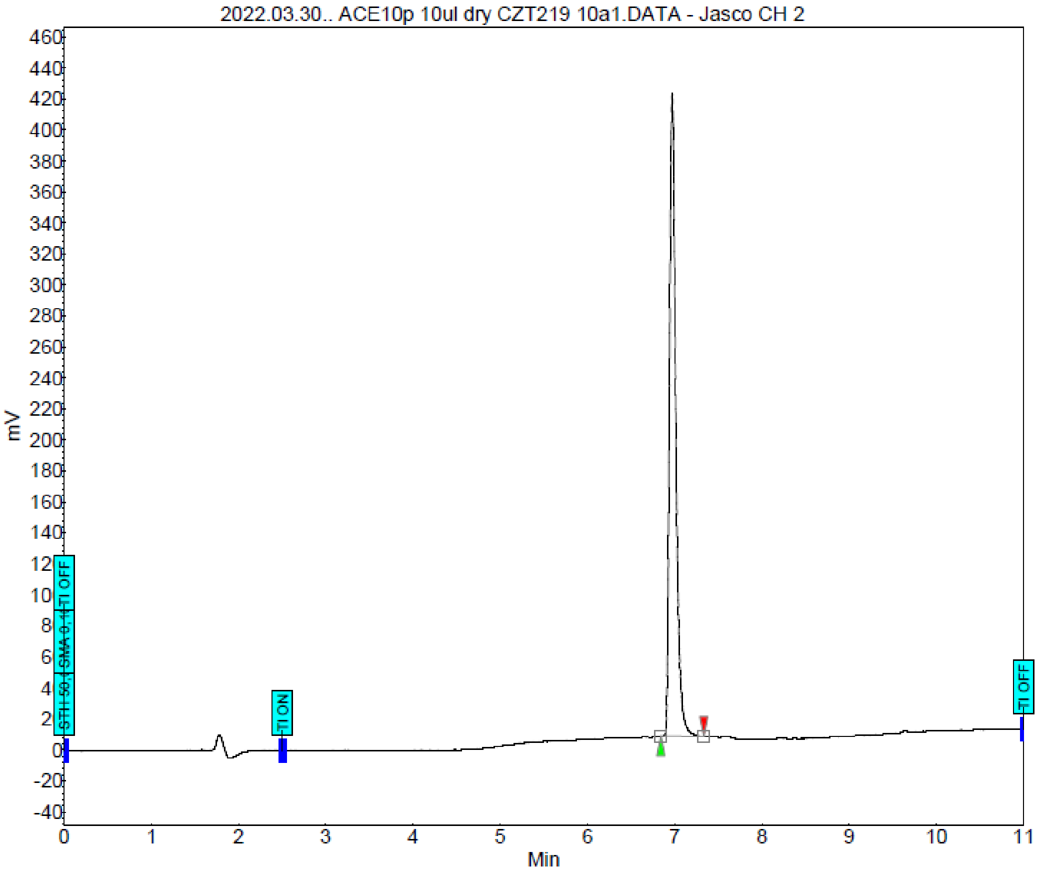

Index	Name	Time [Min]	Quantity [% Area]	Height [mV]	Area [mV.Min]	Area % [%]
1	UNKNOWN	6,970	100,00	414,6	34,845	100,000
Total			100,00	414,6	34,845	100,000

17b

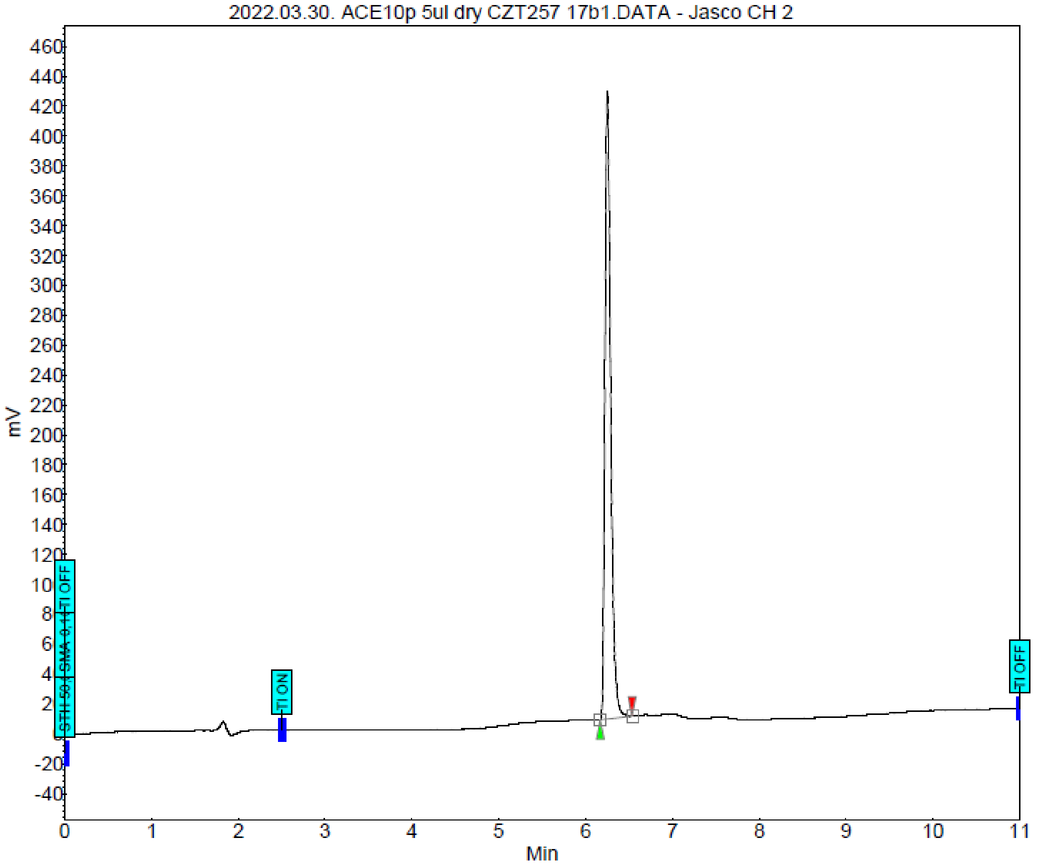

Index	Name	Time [Min]	Quantity [% Area]	Height [mV]	Area [mV.Min]	Area % [%]
1	UNKNOWN	6,252	100,00	420,0	32,562	100,000
Total			100,00	420,0	32,562	100,000

17c

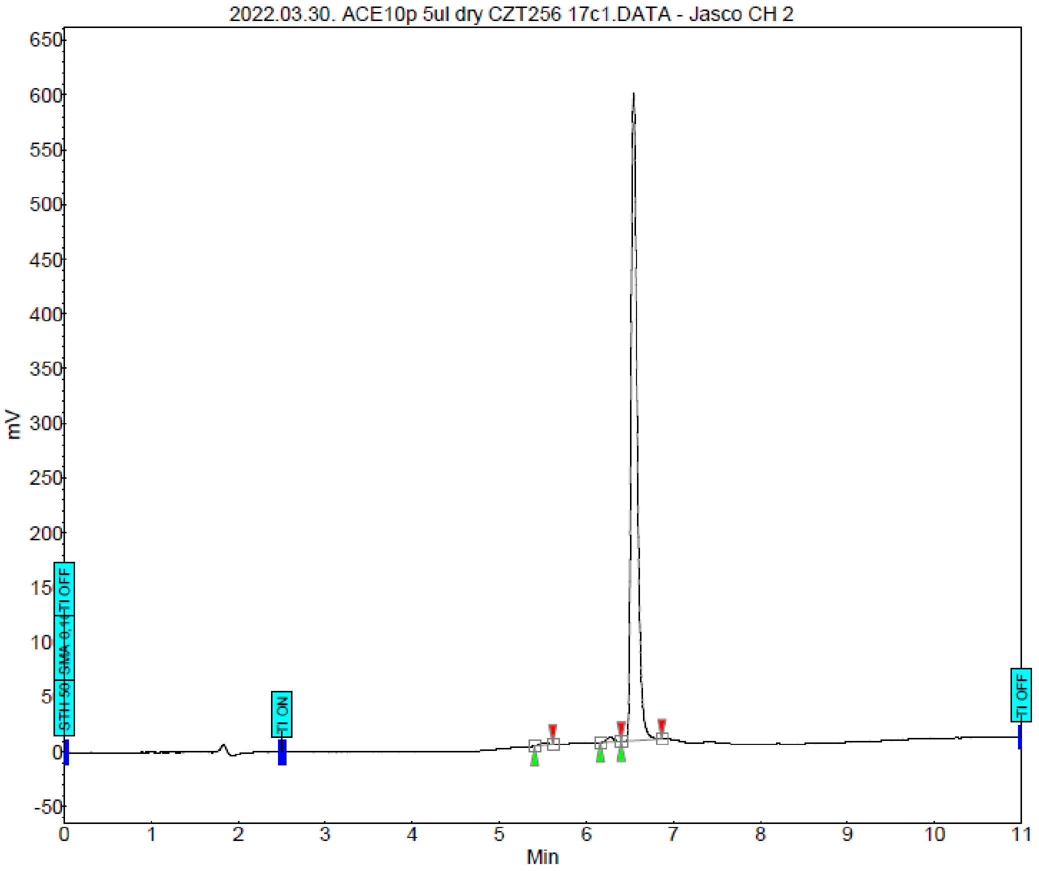

Index	Name	Time [Min]	Quantity [% Area]	Height [mV]	Area [mV.Min]	Area % [%]
1	UNKNOWN	5,500	0,50	2,4	0,242	0,504
2	UNKNOWN	6,272	0,75	4,1	0,359	0,749
3	UNKNOWN	6,542	98,75	591,3	47,277	98,746
Total			100,00	597,8	47,877	100,000

17d

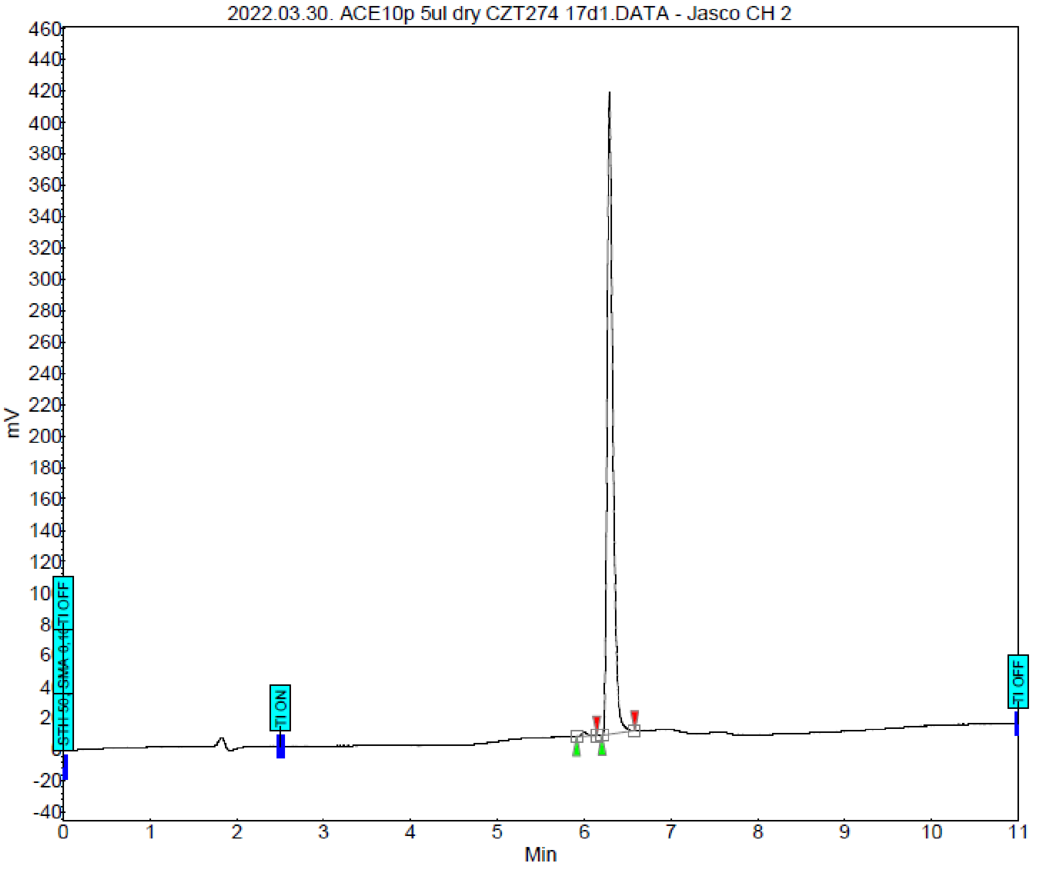

Index	Name	Time [Min]	Quantity [% Area]	Height [mV]	Area [mV.Min]	Area % [%]
1	UNKNOWN	6,002	0,48	2,1	0,155	0,483
2	UNKNOWN	6,292	99,52	409,3	32,006	99,517
Total			100,00	411,4	32,161	100,000

S.2. ^{1}H-, ^{13}C NMR and HRMS data of the targeted compounds

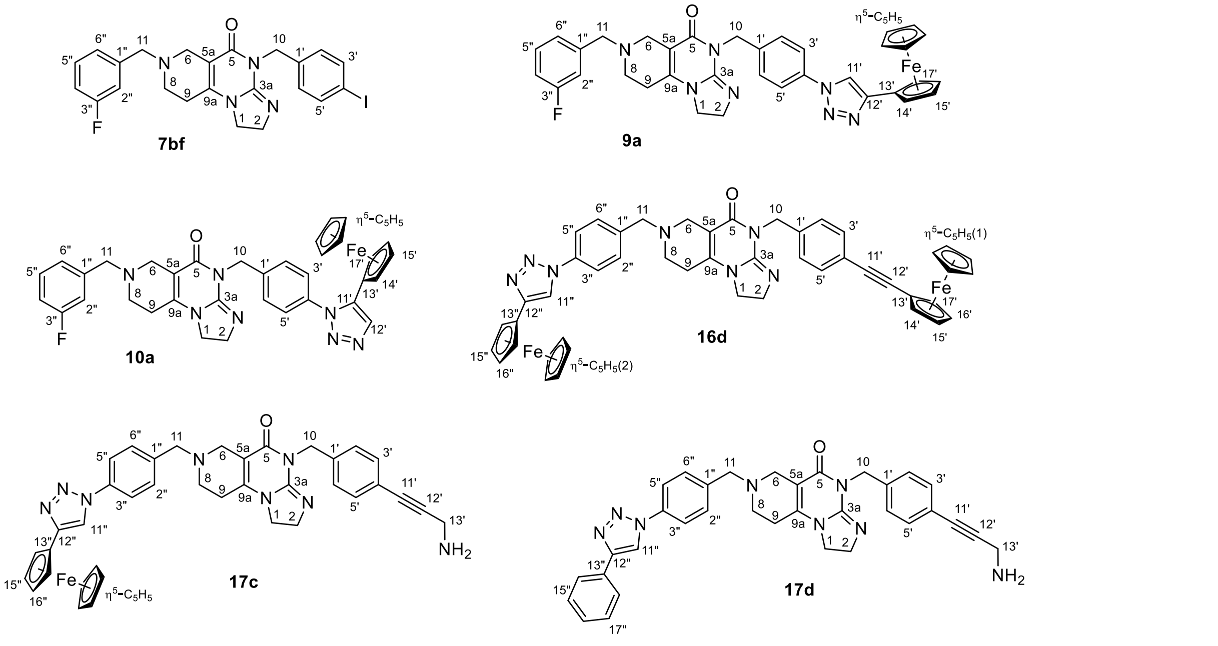

Figure S.5. Numbering of atoms presented on the structures of representative compounds is used for the assignment of ^{1}H- and ^{13}C-NMR data.

7-(3-fluorobenzyl)-4-(4-iodobenzyl)-2,4,6,7,8,9-hexahydroimidazo[1,2-*a*]pyrido[3,4-*e*]pyrimidin-5(1*H*)-one (**7bf**):

^{1}H-NMR (CDCl$_3$): 7.58 (d, *J*=8.4 Hz, 2H, H-3′,5′); 7.25 (m, 1H, H-5″); 7.20 (d, *J*=8.4 Hz, 2H, H-2′,6′); 7.07 (d, *J*=8.3 Hz, 1H, H-2″); 7.05 (dt, *J*=9.6 Hz and 2.0 Hz, 1H, H-6″); 6.94 (tdd, *J*=8.5 Hz, 2.6 Hz and 0.8 Hz, 1H, H-4″); 4.96 (s, 2H, H-10); 3.88 (s, 4H, H-1,2); 3.63 (s, 2H, H-11); 3.26 (br s, 2H, H-6); 2.64, (t, *J*=5.7 Hz, 2H, H-8); 2.46 (t, 2H, *J*=5.7 Hz, H-9). ^{13}C-NMR (CDCl$_3$): 163.0 (d, *J*=246.0 Hz, C-3″); 161.5 (C-5); 152.9 (C-3a);145.2 (C-9a); 140.6 (d, *J*=7.2 Hz, C-1″); 137.3 (C-3′,5′); 136.6 (C-1′); 130.8 (C-2′,6′); 129.8 (d, *J*=8.6 Hz, C-3″); 124.5 (d, *J*=8.3 Hz, C-6″); 115.6 (d, *J*=21.6 Hz, C-2″); 101.8 (C-5a); 93.0 (C-4′); 61.8 (C-11); 50.6 (C-2); 49.4 (C-6); 48.3 (C-8); 46.9 (C-1); 44.9 (C-10); 26.8 (C-9). HRMS: *m*/*z* calc. for $[C_{23}H_{23}FIN_4O]^+$: 517.0895 $[M+H]^+$; found: 517.0885; mass error: 1.96 ppm.

7-(3,5-difluorobenzyl)-4-(4-iodobenzyl)-2,4,6,7,8,9-hexahydroimidazo[1,2-*a*]pyrido[3,4-*e*]pyrimidin-5(1*H*)-one (**7cf**):

^{1}H-NMR (CDCl$_3$): 7.59 (d, *J*=8.4 Hz, 2H, H-3′,5′); 7.20 (d, *J*=8.4 Hz, 2H, H-2′,6′); 6.86 (m, 2H, H-2″,6″); 6.69 (tt, *J*=8.8 Hz and 2.2 Hz, 1H, H-4″); 4.97 (s, 2H, H-10); 3.89 (s, 4H, H-1,2); 3.61 (s, 2H, H-11); 3.24 (br s, 2H, H-6); 2.65, (t, *J*=5.7 Hz, 2H, H-8); 2.47 (t, 2H, *J*=5.7 Hz, H-9). ^{13}C-NMR (CDCl$_3$): 163.1 (dd, *J*=248.0 Hz and 12.9 Hz, C-3″,5″); 161.3 (C-5); 152.9 (C-3a); 145.6 (C-9a); 142.2 (t, *J*=9.4 Hz, C-1″); 137.4 (C-3′,5′); 136.6 (C-1′); 130.8 (C-2′,6′); 111.3 (dd, *J*=19.5 Hz and 4.4 Hz, C-2″,6″); 102.7 (t, *J*=26.0 Hz, C-4″); 101.6 (C-5a); 93.1 (C-4′); 61.4 (C-11); 50.7 (C-2); 49.4 (C-6); 48.4 (C-8); 46.9 (C-1); 44.9 (C-10); 26.8 (C-9). HRMS: *m*/*z* calc. for $[C_{23}H_{22}F_2IN_4O]^+$: 535.0801. $[M+H]^+$; found: 535.0790; mass error: 2.04 ppm.

7-(3-cyanobenzyl)-4-(4-iodobenzyl)-2,4,6,7,8,9-hexahydroimidazo[1,2-*a*]pyrido[3,4-*e*]pyrimidin-5(1*H*)-one (7df):

^{1}H-NMR ($CDCl_3$): 7.64 (br ~t, *J*~2 Hz, 1H, H-2″); 7.58 (d, *J*=8.4 Hz, 2H, H-3′,5′); 7.52-7.57 (overlapping m′s, 2H, H-4″ and H-6″); 7.41 (t, *J*=7.8 Hz, 1H, H-5″); 7.19 (d, *J*=8.4 Hz, 2H, H-2′,6′); 4.96 (s, 2H, H-10); 3.89 (s, 4H, H-1,2); 3.66 (s, 2H, H-11); 3.23 (br s, 2H, H-6); 2.66, (t, *J*=5.6 Hz, 2H, H-8); 2.47 (t, 2H, *J*=5.6 Hz, H-9). ^{13}C-NMR ($CDCl_3$): 161.3 (C-5); 152.9 (C-3a); 145.6 (C-9a); 139.7 (C-1″); 137.4 (C-3′,5′); 136.6 (C-1′); 133.2 (C-2″); 132.3 (C-6″); 131.1 (C-4″); 130.8 (C-2′,6′); 129.3 (C-5″); 118.9 (CN); 112.6 (C-3″); 101.6 (C-5a); 93.1 (C-4′); 61.4 (C-11); 50.6 (C-2); 49.3 (C-6); 48.6 (C-8); 46.9 (C-1); 45.1 (C-10); 26.8 (C-9). HRMS: *m*/*z* calc. for $[C_{24}H_{23}IN_5O]^+$: 524.0942 $[M+H]^+$; found: 524.0934; mass error: 1.50 ppm.

4-(4-Azidobenzyl)-7-(3-fluorobenzyl)-2,4,6,7,8,9-hexahydroimidazo[1,2-*a*]pyrido[3,4-*e*]pyrimidin-5(1*H*)-one (7bh):

^{1}H-NMR ($CDCl_3$): 7.47 (d, *J*=8.4 Hz, 2H, H-2′,6′); 7.27 (m, 1H, H-5″); 7.08 (br d, *J*=8.4 Hz, 1H, H-6″); 7.07 (dt, *J*=9.6 Hz and 2.0 Hz, 1H, H-2″); 6.95 (m, 1H, H-4″); 6.93 (d, *J*=8.3 Hz, 2H, H-3′,5′); 5.00 (s, 2H, H-10); 3.91-3.84 (m, 4H, H-1 and H-2); 3.64 (s, 2H, H-11); 3.27 (br s, 2H, H-6); 2.65 (t, *J*=5.7 Hz, 2H, H-8); 2.47 (t, *J*=5.7 Hz, 2H, H-9). ^{13}C-NMR ($CDCl_3$): 163.0 (d, *J*=246.2 Hz, C-3″); 161.4 (C-5); 153.1 (C-3a); 145.6 (C-9a); 140.6 (d, *J*=7.0 Hz, C-1″); 139.1 (C-4′); 133.8 (C-1′); 130.6 (C-2′,6′); 129.8 (d, *J*=8.8 Hz, C-5″); 124.5 (C-6″); 118.9 (C-3′,5′); 115.6 (d, *J*=21.5 Hz, C-6″); 114.3 (d, *J*=21.0 Hz, C-4″); 101.8 (C-5a); 61.8 (C-11); 50.6 (C-2); 49.4 (C-6); 48.4 (C-8); 46.9 (C-1); 44.9 (C-10); 27.0 (C-9). HRMS: *m*/*z* calc. for $[C_{23}H_{22}FN_7O]^+$: 432.1943 $[M+H]^+$; found: 432.1936; mass error: 1.53 ppm.

4-(4-Azidobenzyl)-7-(3,5-difluorobenzyl)-2,4,6,7,8,9-hexahydroimidazo[1,2-*a*]pyrido[3,4-*e*]pyrimidin-5(1*H*)-one (7ch):

^{1}H-NMR ($CDCl_3$): 7.44 (d, *J*=8.3 Hz, 2H, H-2′,6′); 6.90 (d, *J*=8.3 Hz, 2H, H-3′,5′); 6.84 (br ~dt, *J*~7 Hz and ~2 Hz, 2H, H-2″,6″); 6.66 (tt, *J*=9.0 Hz and 2.3 Hz, 1H, H-4″); 4.97 (s, 2H, H-10); 3.91-3.84 (m, 4H, H-1 and H-2); 3.59 (s, 2H, H-11); 3.23 (br s, 2H, H-6); 2.63 (t, *J*=5.7 Hz, 2H, H-8); 2.44 (t, *J*=5.7 Hz, 2H, H-9). ^{13}C-NMR ($CDCl_3$): 163.1 (dd, *J*=250.2 Hz and 15.6 Hz, C-3″,5″); 161.4 (C-5); 152.9 (C-3a); 145.5 (C-9a); 142.2 (t, *J*=8.4 Hz, C-1″); 139.1 (C-4′); 133.8 (C-1′); 130.4 (C-2′,6′); 118.9 (C-3′,5′); 111.3 (dd, *J*=19.3 Hz and 4.9 Hz, C-2″,6″); 102.7 (t, *J*=25.7 Hz, C-4″); 101.7 (C-5a); 61.4 (C-11); 50.6 (C-2); 49.4 (C-6); 48.4 (C-8); 46.9 (C-1); 44.8 (C-10); 26.8 (C-9). HRMS: *m*/*z* calc. for $[C_{23}H_{22}F_2N_7O]^+$: 450.1848 $[M+H]^+$; found: 450.1843; mass error: 1.20 ppm.

7-(4-Azidobenzyl)-4-(4-iodobenzyl)-2,4,6,7,8,9-hexahydroimidazo[1,2-*a*]pyrido[3,4-*e*]pyrimidin-5(1*H*)-one (7hf):

^{1}H-NMR ($CDCl_3$): 7.59 (d, *J*=8.2 Hz, 2H, H-3′,5′); 7.30 (d, *J*=8.3 Hz, 2H, H-2″,6″); 7.20 (d, *J*=8.3 Hz, 2H, H-2′,6′); 6.97 (d, *J*=8.3, 2H, H-3″,5″); 4.97 (s, 2H, H-10); 3.88 (s, 4H, H-1 and H-2); 3.62 (s, 2H, H-11); 3.24 (br s, 2H, H-6); 2.64 (t, *J*=5.6 Hz, 2H, H-8); 2.45 (t, *J*=5.6 Hz, 2H, H-9). ^{13}C-NMR ($CDCl_3$): 161.3 (C-5); 152.9 (C-3a); 145.7 (C-9a); 139.1 (C-4′); 137.3 (C-3′,5′); 136.6 (C-1′); 134.5 (C-1″); 130.8 (C-2′,6′); 130.5 (C-2″,6″), 119.1 (C-3″,5″); 101.8 (C-5a); 93.0 (C-4′); 61.6 (C-

11); 50.6 (C-2); 49.3 (C-6); 48.3 (C-8); 46.9 (C-1); 44.9 (C-10); 26.8 (C-9). HRMS: *m*/*z* calc. for $[C_{23}H_{23}IN_7O]^+$: 540.1003 $[M+H]^+$; found: 540.0994; mass error: 1.73 ppm.

7-(4-Azidobenzyl)-4-(2-methylbenzyl)-2,4,6,7,8,9-hexahydroimidazo[1,2-*a*]pyrido[3,4-*e*]pyrimidin-5(1*H*)-one (7hg):

^{1}H-NMR ($CDCl_3$): 7.31 (d, *J*=8.3 Hz, 2H, H-2″,6″); 7.11-7.06 (overlapping m′s, 3H, H-3′,4′,5′); 7.03 (m, 1H, H-6′); 6.96 (d, *J*=8.3, 2H, H-3″,5″); 5.03 (s, 2H, H-10); 3.91 (~t, *J*~10 Hz, 2H, H-2); 3.86 (~t, *J*~10 Hz, 2H, H-1); 3.62 (s, 2H, H-11); 3.27 (br s, 2H, H-6); 2.66 (t, *J*=5.6 Hz, 2H, H-8); 2.50 (t, *J*=5.6 Hz, 2H, H-9); 2.38 (s, 3H, $C\underline{H}_3$). ^{13}C-NMR ($CDCl_3$): 161.5 (C-5); 153.2 (C-3a); 145.7 (C-9a); 139.1 (C-4′); 135.6 (C-1′); 134.6 (C-1″); 134.2 (C-2′); 130.5 (C-2″,6″), 130.2 (C-5′); 126.8 (C-4′); 125.9 (C-3′); 125.2 (C-6′); 119.0 (C-3″,5″); 101.8 (C-5a); 61.6 (C-11); 50.6 (C-2); 49.4 (C-6); 48.3 (C-8); 46.9 (C-1); 43.2 (C-10); 26.8 (C-9); 19.2 ($\underline{C}H_3$). HRMS: *m*/*z* calc. for $[C_{24}H_{26}N_7O]^+$: 428.2193 $[M+H]^+$; found: 428.2185; mass error: 1.95 ppm.

7-(3-Fluorobenzyl)-4-(4-(4-ferrocenyl-1*H*-1,2,3-triazol-1-yl)benzyl)-2,4,6,7,8,9-hexahydro-imidazo[1,2-*a*]pyrido[3,4-*e*]pyrimidin-5(1*H*)-one (9a):

^{1}H-NMR ($CDCl_3$): 7.84 (s, 1H, H-11′); 7.68, (d, *J*=8.4 Hz, 2H, H-3′,5′); 7.63, (d, *J*=8.4 Hz, 2H, H-2′,6′); 7.26 (m, 1H, H-5″); 7.09 (d, *J*=8.3 Hz, 1H, H-6″); 7.06 (dt, *J*=9.5 Hz and 2.0 Hz, 1H, H-2″); 6.95 (td, *J*=8.6 Hz and 2.4 Hz, 1H, H-4″); 5.10 (s, 2H, H-10); 4.77 (br ~s, 2H, H-14′,17′); 4.32 (br ~s, 2H, H-15′,16′); 4.10 (s, 5H, η^5-$C_5\underline{H}_5$); 3.91 (s, 4H, H-1,2); 3.65 (s, 2H, H-11); 3.29 (br ~s, 2H, H-6); 2.67 (t, *J*=5.6 Hz, 2H, H-8); 2.48 (t, *J*=5.7 Hz, 2H, H-9). ^{13}C-NMR ($CDCl_3$): 163.0 (d, *J*=246.0 Hz C-3″); 161.4 (C-6); 152.9 (C-3a); 147.5 (C-12′); 145.8 (C-9a); 140.5 (d, *J*=7.1 Hz, C-1″); 137.6 (C-4′); 136.2 (C-1′); 130.1 (C-2′,6′); 129.8 (d, *J*=8.5 Hz, C-5″); 124.5 (C-6″); 120.1 (C-3′,5′); 116.5 (C-11′); 115.6 (d, *J*=21.6 Hz, C-2″); 114.3 (d, *J*=21.2 Hz, C-4″); 101.8 (C-5a); 75.0 (C-13′); 69.6 (η^5-$\underline{C}_5H_5$); 68.8 (15′,16′); 66.8 (C-14′,17′); 61.7 (C-11); 50.6 (C-2); 49.4 (C-6); 48.3 (C-8); 46.9 (C-1); 44.8 (C-10); 26.8 (C-9). HRMS: *m*/*z* calc. for $[C_{35}H_{32}FFeN_7O]^+$: 641.1996 $[M - e^-]^+$; found: 641.1984; mass error: 1.87 ppm

4-(4-Iodobenzyl)-7-(4-(4-ferrocenyl-1*H*-1,2,3-triazol-1-yl)benzyl)-2,4,6,7,8,9-hexahydroimidazo[1,2-*a*]pyrido[3,4-*e*]pyrimidin-5(1*H*)-one (12a):

^{1}H-NMR ($CDCl_3$): 7.87 (s, 1H, H-11″); 7.71 (d, *J*=8.4 Hz, 2H, H-3″,5″); 7.60 (d, *J*=8.1 Hz, 2H, H-3′,5′); 7.50 (d, *J*=8.4 Hz, 2H, H-2″,6″); 7.21 (d, *J*=8.1 Hz, 2H, H-2′,6′); 4.98 (s, 2H, H-10); 4.79 (t, *J*=1.7 Hz, 2H, H-14″,17″); 4.33 (t, *J*=1.7 Hz, 2H, H-15″,16″); 4.12 (s, 5H, η^5-$C_5\underline{H}_5$); 3.90 (br ~s, 4H, H-1,2); 3.72 (s, 2H, H-11); 3.29 (br ~s, 2H, H-6); 2.69 (t, *J*=5.7 Hz, 2H, H-8); 2.48 (t, *J*=5.7 Hz, 2H, H-9). ^{13}C-NMR ($CDCl_3$): 161.3 (C-6); 152.9 (C-3a); 147.6 (C-12″); 145.6 (C-9a); 138.6 (C-1″); 137.4 (C-3′,5′); 136.6 (C-1′); 136.3 (C-4″); 130.8 (C-2′,6′); 130.2 (C-2″,6″); 120.4 (C-3″,5″); 116.6 (C-11″); 101.7 (C-5a); 93.0 (C-4′); 75.0 (C-13″); 69.6 (η^5-$\underline{C}_5H_5$); 68.8 (C-15″,16″); 66.8 (C-14″,17″); 61.6 (C-11); 50.8 (C-2); 49.4 (C-6); 48.4 (C-8); 46.9 (C-1); 44.9 (C-10); 26.8 (C-9). HRMS: *m*/*z* calc. for $[C_{35}H_{33}FeIN_7O]^+$: 750.1135 $[M+H]^+$; found: 750.1112; mass error: 3.09 ppm

4-(2-Methylbenzyl)-7-(4-(4-ferrocenyl-1*H*-1,2,3-triazol-1-yl)benzyl)-2,4,6,7,8,9-hexahydroimidazo[1,2-*a*]pyrido[3,4-*e*]pyrimidin-5(1*H*)-one (12b):

^{1}H-NMR ($CDCl_3$): 7.88 (s, 1H, H-11″); 7.72 (d, *J*=8.4 Hz, 2H, H-3″,5″); 7.49 (d, *J*=8.4 Hz, 2H, H-2″,6″); 7.13 (overlapping m's, 3H, H-3′,4′,5′); 7.04 (m, 1H, H-6′); 5.04 (s, 2H, H-10); 4.78 (t, *J*=1.7 Hz, 2H, H-14″,17″); 4.33 (t, *J*=1.7 Hz, 2H, H-15″,16″); 4.10 (s, 5H, η^5-$C_5\underline{H}_5$); 3.92 (~t, *J*~10 Hz, 2H, H-1); 3.88 (~t, *J*~10 Hz, 2H, H-2); 3.72 (s, 2H, H-11); 3.31 (br ~s, 2H, H-6); 2.71 (t, *J*=5.6 Hz, 2H, H-8); 2.52 (t, *J*=5.6 Hz, 2H, H-9); 2.39 (s, 3H, $C\underline{H}_3$). ^{13}C-NMR ($CDCl_3$): 161.5 (C-6); 153.3 (C-3a); 147.7 (C-12″); 145.6 (C-9a); 138.6 (C-1″); 136.2 (C-4″); 135.6 (C-2′); 134.3 (C-1′); 130.3 (C-2″,6″); 130.2 (C-3′); 126.8 (C-5′); 125.9 (C-4′); 125.2 (C-6′); 120.4 (C-3″,5″); 116.6 (C-11″); 101.7 (C-5a); 74.9 (C-13″); 69.6 (η^5-$\underline{C}_5H_5$); 68.8 (C-15″,16″); 66.8 (C-14″,17″); 61.6 (C-11); 50.6 (C-2); 49.5 (C-6); 48.4 (C-8); 47.0 (C-1); 43.2 (C-10); 26.8 (C-9); 19.3 ($\underline{C}H_3$). HRMS: *m/z* calc. for $[C_{36}H_{36}FeN_7O]^+$: 638.2325 $[M+H]^+$; found: 638.2305; mass error: 3.17 ppm.

4-(4-Iodobenzyl)-7-(4-(4-phenyl-1*H*-1,2,3-triazol-1-yl)benzyl)-2,4,6,7,8,9-hexahydroimidazo[1,2-*a*]pyrido[3,4-*e*]pyrimidin-5(1*H*)-one (13):

^{1}H-NMR ($CDCl_3$): 8.18 (s, 1H, H-11″); 7.92 (dd, *J*=7.8 Hz and 2.1 Hz, 2H, H-14″,18″); 7.74 (d, *J*=8.4 Hz, 2H, H-3″,5″); 7.60 (d, *J*=8.3 Hz, 2H, H-3′,5′); 7.51 (d, *J*=8.4 Hz, 2H, H-2″,6″); 7.47 (t, *J*=7.8 Hz, 2H, H-15″,17″); 7.37 (tt, *J*=7.8 Hz and 2.1 Hz, 1H, H-16″); 7.21 (d, *J*=8.3 Hz, 2H, H-2′,6′); 4.98 (s, 2H, H-10); 3.90 (br ~s, 4H, H-1,2); 3.72 (s, 2H, H-11); 3.30 (br ~s, 2H, H-6); 2.70 (t, *J*=5.6 Hz, 2H, H-8); 2.49 (t, *J*=5.6 Hz, 2H, H-9). ^{13}C-NMR ($CDCl_3$): 161.4 (C-6); 153.0 (C-3a); 148.6 (C-12″); 145.8 (C-9a); 139.0 (two coalesced lines, C-1″ and C-13″); 137.3 (C-3′,5′); 136.8 (C-1′); 136.4 (C-4″); 130.8 (C-2′,6′); 130.4 (C-2″,6″); 128.8 (C-15″,17″); 128.3 (C-16″); 125.7 (C-14″,18″); 120.7 (C-3″,5″); 117.4 (C-11″); 101.7 (C-5a); 93.1 (C-4′); 61.7 (C-11); 50.5 (C-2); 49.5 (C-6); 48.4 (C-8); 47.0 (C-1); 45.0 (C-10); 27.1 (C-9). HRMS: *m/z* calc. for $[C_{31}H_{29}IN_7O]^+$: 642.1473 $[M+H]^+$; found: 642.1455; mass error: 2.78 ppm

7-(3-Fluorobenzyl)-4-(4-(5-ferrocenyl-1*H*-1,2,3-triazol-1-yl)benzyl)-2,4,6,7,8,9-hexahydroimidazo[1,2-*a*]pyrido[3,4-*e*]pyrimidin-5(1*H*)-one (10a):

^{1}H-NMR ($CDCl_3$): 7.79 (s, 1H, H-12′); 7.57 (d, *J*=8.2 Hz, 2H, H-2′,6′); 7.33 (d, *J*=8.2 Hz, 2H, H-3′,5′); 7.27 (m, 1H, H-5″); 7.09 (d, *J*=8.4 Hz, 1H, H-6″); 7.06 (dt, *J*=9.4 Hz and 2.2 Hz, 1H, H-2″); 6.95 (dt, *J*=8.6 Hz and 2.2 Hz, 1H, H-4″); 5.12 (s, 2H, H-10); 4.24 (br ~s, 2H, H-14′,17′); 4.20 (br ~s, 2H, H-15′,16′); 4.05 (s, 5H, η^5-$C_5\underline{H}_5$); 3.90 (br ~s, 4H, H-1,2); 3.65 (s, 2H, H-11); 3.30 (br ~s, 2H, H-6); 2.67 (t, *J*=5.7 Hz, 2H, H-8); 2.49 (t, *J*=5.7 Hz, 2H, H-9). ^{13}C-NMR ($CDCl_3$): 163.0 (d, *J*=246.1 Hz, C-3″); 161.3 (C-6); 152.9 (C-3a); 145.8 (C-9a); 140.5 (d, *J*=6.8 Hz, C-1″); 138.7 (C-1′); 137.2 (C-11′), 135.9 (C-4′); 132.4 (C-11′); 129.9 (d, *J*=7.6 Hz, C-5″); 129.4 (C-2′,6′); 125.8 (C-3′,5′); 124.5 (C-6″); 115.7 (d, *J*=21.7 Hz, C-2″); 114.3 (d, *J*=21.1 Hz, C-4″); 101.7 (C-5a); 70.5 (C-13′); 69.9 (η^5-$\underline{C}_5H_5$); 69.4 (C-15′,16′); 68.5 (C-14′,17′); 61.7 (C-11); 50.6 (C-2); 49.4 (C-6); 48.3 (C-8); 46.9 (C-1); 44.9 (C-10); 26.8 (C-9). HRMS: *m/z* calc. for $[C_{35}H_{33}FFeN_7O]^+$: 642.2075 $[M+H]^+$; found: 642.2058; mass error: 2.57 ppm

7-(3,5-Difluorobenzyl)-4-(4-(5-ferrocenyl-1*H*-1,2,3-triazol-1-yl)benzyl)-2,4,6,7,8,9-hexahydroimidazo[1,2-a]pyrido[3,4-*e*]pyrimidin-5(1*H*)-one (10b):

^{1}H-NMR ($CDCl_3$): 7.80 (s, 1H, H-12′); 7.58 (d, *J*=8.2 Hz, 2H, H-2′,6′); 7.33 (d, *J*=8.2 Hz, 2H, H-3′,5′); 6.88 (br ~d, *J*~6 Hz, 2H, H-2″,6″); 6.70 (tt, *J*=8.7 Hz and 1.9 Hz, 1H, H-4″); 5.13 (s, 2H, H-

10); 4.25 (br ~s, 2H, H-14′,17′); 4.20 (br ~s, 2H, H-15′,16′); 4.06 (s, 5H, η[5]-$C_5\underline{H}_5$); 3.92 (br ~s, 4H, H-1,2); 3.64 (s, 2H, H-11); 3.29 (br ~s, 2H, H-6); 2.68 (t, *J*=5.7 Hz, 2H, H-8); 2.51 (t, *J*=5.7 Hz, 2H, H-9). ^{13}C-NMR ($CDCl_3$): 163.1 (dd, *J*=248.6 Hz and 12.7 Hz, C-3″, 5″); 161.3 (C-6); 152.8 (C-3a); 145.8 (C-9a); 142.2 (t, *J*=8.4 Hz, C-1″); 138.7 (C-1′); 137.2 (C-11′), 136.0 (C-4′); 132.4 (C-11′); 129.4 (C-2′,6′); 125.8 (C-3′,5′); 111.4 (dd, *J*=20.2 Hz and 4.7 Hz, C-2″,6″); 102.8 (t, *J*=25.6 Hz C-4″); 101.6 (C-5a); 70.6 (C-13′); 69.9 (η[5]-$\underline{C}_5H_5$); 69.3 (C-15′,16′); 68.5 (C-14′,17′); 61.4 (C-11); 50.6 (C-2); 49.4 (C-6); 48.4 (C-8); 46.9 (C-1); 44.9 (C-10); 26.8 (C-9). HRMS: *m*/*z* calc. for $[C_{35}H_{32}F_2FeN_7O]^{2+}$: 330.6027 $[M+2H]^{2+}$; found: 330.6019; mass error: 2.28 ppm

4-(4-Iodobenzyl)-7-(4-(5-ferrocenyl-1*H*-1,2,3-triazol-1-yl)benzyl)-2,4,6,7,8,9-hexahydroimidazo[1,2-*a*]pyrido[3,4-*e*]pyrimidin-5(1*H*)-one (11):

^{1}H-NMR ($CDCl_3$): 7.81 (s, 1H, H-12″); 7.60 (d, *J*=8.4 Hz, 2H, H-3′,5′); 7.47 (d, *J*=8.6 Hz, 2H, H-2″,6″); 7.37 (d, *J*=8.6 Hz, 2H, H-3″,5″); 7.22 (d, *J*=8.4 Hz, 2H, H-2′,6′); 4.99 (s, 2H, H-10); 4.26 (br ~s, 2H, H-14″,17″); 4.21 (br ~s, 2H, H-15″,16″); 4.07 (s, 5H, η[5]-$C_5\underline{H}_5$); 3.90 (br ~s, 4H, H-1,2); 3.74 (s, 2H, H-11); 3.33 (br ~s, 2H, H-6); 2.69 (t, *J*=5.7 Hz, 2H, H-8); 2.48 (t, *J*=5.7 Hz, 2H, H-9). ^{13}C-NMR ($CDCl_3$): 161.4 (C-6); 153.0 (C-3a); 145.6 (C-9a); 139.9 (C-1″); 137.5 (C-3′,5′); 137.3 (C-11″); 136.8 (C-1′); 136.1 (C-4″); 132.3 (C-12″); 130.8 (C-2′,6′); 129.7 (C-2″,6″); 126.2 (C-3″,5″); 101.9 (C-5a); 93.1 (C-4′); 70.5 (C-13″); 70.0 (η[5]-$\underline{C}_5H_5$); 69.4 (C-15″,16″); 68.7 (C-14″,17″); 61.6 (C-11); 50.7 (C-2); 49.7 (C-6); 48.3 (C-8); 46.9 (C-1); 45.0 (C-10); 26.8 (C-9). HRMS: *m*/*z* calc. for $[C_{35}H_{33}FeIN_7O]^{+}$: 750.1135 $[M+H]^{+}$; found: 750.1112; mass error: 3.09 ppm

7-(3-Fluorobenzyl)-4-(4-(ferrocenylethynyl)benzyl)-2,4,6,7,8,9-hexahydroimidazo[1,2-*a*]pyrido[3,4-*e*]pyrimidin-5(1*H*)-one (16a):

^{1}H-NMR ($CDCl_3$): 7.40-7.38, (overlapping m′s, 4H, H-2′,3′,5′,6); 7.25 (m, 1H, H-5″); 7.08 (d, *J*=8.4 Hz, 1H, H-6″); 7.05 (dt, *J*=9.7 Hz and 2.0 Hz, 1H, H-2″); 6.93 (td, *J*=8.5 Hz and 2.1 Hz, 1H, H-4″); 5.03 (s, 2H, H-10); 4.47 (br ~s, 2H, H-14′,17′); 4.22 (coalesced br ~s′s, 7H, H-15′,16′ and η[5]-$C_5\underline{H}_5$); 3.89 (s, 4H, H-1,2); 3.63 (s, 2H, H-11); 3.26 (br ~s, 2H, H-6); 2.64 (t, *J*=5.7 Hz, 2H, H-8); 2.46 (t, *J*=5.7 Hz, 2H, H-9). ^{13}C-NMR ($CDCl_3$): 163.0 (d, *J*=246.0 Hz C-3″); 161.4 (C-6); 152.9 (C-3a); 145.6 (C-9a); 140.6 (d, *J*=7.3 Hz, C-1″); 136.4 (C-1′); 131.3 (C-2′,6′); 129.8(d, *J*=8.6 Hz, C-5″); 128.6 (C-3′,5′); 124.5 (C-6″); 123.0 (C-4′); 115.6 (d, *J*=21.6 Hz, C-2″); 114.3 (d, *J*=21.3 Hz, C-4″); 101.8 (C-5a); 88.2 (C-12′); 85.8 (C-11′); 71.4 (C-14′,17′); 70.0 (η[5]-$\underline{C}_5H_5$); 68.8 (C-15′,16′); 65.4 (C-13′); 61.7 (C-11); 50.6 (C-2); 49.5 (C-6); 48.3 (C-8); 46.8 (C-1); 45.2 (C-10); 26.8 (C-9). HRMS: *m*/*z* calc. for $C_{35}H_{32}FFeN_4O]^{+}$: 599.1904 $[M+H]^{+}$; found: 599.1888; mass error: 2.67 ppm.

7-(3,5-Difluorobenzyl)-4-(4-(ferrocenylethynyl)benzyl)-2,4,6,7,8,9-hexahydroimidazo[1,2-a]pyrido[3,4-*e*]pyrimidin-5(1*H*)-one (16b):

^{1}H-NMR ($CDCl_3$): 7.55 (dd, *J*=7.8 Hz and 1,7 Hz, 2H, H-2″,6″); 7.40-7.38, (overlapping m′s, 4H, H-2′,3′,5′,6); 6.70 (tt, *J*=8.8 Hz and 1.7 Hz, 1H, H-4″); 5.03 (s, 2H, H-10); 4.47 (br ~s, 2H, H-14′,17′); 4.21 (coalesced br ~s′s, 7H, H-15′,16′ and η[5]-$C_5\underline{H}_5$); 3.89 (s, 4H, H-1,2); 3.62 (s, 2H, H-11); 3.27 (br ~s, 2H, H-6); 2.66 (t, *J*=5.7 Hz, 2H, H-8); 2.47 (t, *J*=5.7 Hz, 2H, H-9). ^{13}C-NMR ($CDCl_3$): 163.1 (dd, *J*=248.0 Hz and 12.9 Hz, C-3″,5″); 161.5 (C-6); 153.0 (C-3a); 145.6 (C-9a);

142.3 (t, *J*=9.3 Hz, C-1″); 136.4 (C-1′); 131.3 (C-2′,6′); 128.7 (C-3′,5′); 123.0 (C-4′); 111.4 (dd, *J*=19.5 Hz and 4.4 Hz, C-2″,6″); 102.7 (t, *J*=26.0 Hz, C-4″); 101.6 (C-5a); 88.2 (C-12′); 85.7 (C-11′); 71.4 (C-14′,17′); 70.0 (η5-C5H5); 68.8 (C-15′,16′); 65.4 (C-13′); 61.4 (C-11); 50.6 (C-2); 49.4 (C-6); 48.4 (C-8); 46.9 (C-1); 45.3 (C-10); 26.8 (C-9). HRMS: *m*/*z* calc. for $[C_{35}H_{31}F_2FeN_4O]^+$: 617.1810 $[M+H]^+$; found: 617.1793, mass error: 2.75 ppm

3-((5-Oxo-4-(4-(ferrocenylethynyl)benzyl)-1,2,4,5,8,9-hexahydroimidazo[1,2-*a*]pyrido[3,4-*e*]pyrimidin-7(6*H*)-yl)methyl)benzonitrile (16c):

^{1}H-NMR ($CDCl_3$): 7.65 (br s, 1H, H-2″); 7.55 (coalesced br ~d's, *J*~8 Hz, 2H, H-4″,6″); 7.42 (t, *J*=7.6 Hz, 1H, H-5″); 7.40-7.38, (overlapping m's, 4H, H-2′,3′,5′,6); 5.03 (s, 2H, H-10); 4.47 (br ~s, 2H, H-14′,17′); 4.21 (coalesced br ~s's, 7H, H-15′,16′ and η5-C5H5); 3.92-3.87 (overlapping m's, 4H, H-1,2); 3.67 (s, 2H, H-11); 3.25 (br ~s, 2H, H-6); 2.65 (t, *J*=5.7 Hz, 2H, H-8); 2.47 (t, *J*=5.7 Hz, 2H, H-9). ^{13}C-NMR ($CDCl_3$): 161.5 (C-6); 152.9 (C-3a); 145.6 (C-9a); 139.8 (C-1″); 136.6 (C-1′); 131.3 (C-2′,6′); 133.3 (C-2″); 132.3 (C-6″); 131.1 (C-4″); 129.2 (C-5″); 128.7 (C-3′,5′); 123.0 (C-4′); 118.8 (CN); 112.6 (C-3″); 101.5 (C-5a); 88.2 (C-12′); 85.7 (C-11′); 71.4 (C-14′,17′); 69.9 (η5-C5H5); 68.8 (C-15′,16′); 65.4 (C-13′); 61.3 (C-11); 50.6 (C-2); 49.4 (C-6); 48.5 (C-8); 46.9 (C-1); 45.2 (C-10); 26.7 (C-9). HRMS: *m*/*z* calc. for $[C_{36}H_{32}FeN_5O]^+$: 606.1951 $[M+H]^+$; found: 606.1930; mass error: 3.42 ppm

7-(4-(4-Ferrocenyl-1*H*-1,2,3-triazol-1-yl)benzyl)-4-(4-(ferrocenylethynyl)benzyl)-2,4,6,7,8,9-hexahydroimidazo[1,2-*a*]pyrido[3,4-*e*]pyrimidin-5(1*H*)-one (16d):

^{1}H-NMR ($CDCl_3$): 7.88 (s, 1H, H-11″); 7.73 (d, *J*=8.2 Hz, 2H, H-3″,5″); 7.50 (d, *J*=8.2 Hz, 2H, H-2″,6″); 7.40-7.38, (overlapping m's, 4H, H-2′,3′,5′,6); 5.04 (s, 2H, H-10); 4.79 (br ~s, 2H, H-14″,17″); 4.48 (br ~s, 2H, H-14′,17′); 4.34 (br ~s, 2H, H-15″,16″); 4.22 (coalesced br ~s's, 7H, H-15′,16′ and ◎5-C5H5 (1)); 4.12 (s, 5H, η5-C5H5 (2)); 3.90 (br ~s, 4H, H-1,2); 3.74 (s, 2H, H-11); 3.31 (br ~s, 2H, H-6); 2.70 (t, *J*=5.7 Hz, 2H, H-8); 2.49 (t, *J*=5.7 Hz, 2H, H-9). ^{13}C-NMR ($CDCl_3$): 161.5 (C-6); 153.1 (C-3a); 147.7 (C-12″); 145.6 (C-9a); 138.6 (C-1″); 136.5 (C-1′); 136.3 (C-4″); 131.3 (C-2′,6′); 130.3 (C-2″,6″); 128.7 (C-3′,5′); 123.1 (C-4′); 120.4 (C-3″,5″); 116.7 (C-11″); 101.7 (C-5a); 88.2 (C-12′); 85.7 (C-11′); 75.1 (C-13″); 71.4 (C-14′,17′); 70.0 (η5-C5H5(1)); 69.6 (η5-C5H5(2)); 68.82 (C-15″,16″); 68.77 (C-15′,16′); 66.9 (C-14″,17″); 65.4 (C-13′); 61.6 (C-11); 50.6 (C-2); 49.4 (C-6); 48.4 (C-8); 46.9 (C-1); 45.3 (C-10); 26.8 (C-9). HRMS: *m*/*z* calc. for $[C_{47}H_{42}Fe_2N_7O]^+$: 832.2144 $[M+H]^+$; found: 832.2120; mass error: 2.90 ppm.

3-((4-(4-(3-Aminoprop-1-yn-1-yl)benzyl)-5-oxo-1,2,4,5,8,9-hexahydroimidazo[1,2-*a*]pyrido[3,4-*e*]pyrimidin-7(6*H*)-yl)methyl)benzonitrile (17a):

^{1}H-NMR ($CDCl_3$): 7.65 (br s, 1H, H-2″); 7.55 (two coalesced dd's, *J*=7.6 Hz and 1.4 Hz, 2H, H-4″,6″); 7.41 (t, *J*=7.6 Hz, 1H, H-5″); 7.37 (d, *J*=8.3 Hz, 2H, H-2′,6′); 7.31 (d, *J*=8.3 Hz, 2H, H-3′,5′); 5.01 (s, 2H, H-10); 3.89 (br ~s, 4H, H-1,2); 3.67 (s, 2H, H-11); 3.25 (br ~s, 2H, H-6); 3.62 (br s, 2H, H-13′); 2.67 (t, *J*=5.7 Hz, 2H, H-8); 2.48 (t, *J*=5.7 Hz, 2H, H-9); 1.65 (br s, 2H, NH_2). ^{13}C-NMR ($CDCl_3$): 161.3 (C-6); 153.1 (C-3a); 145.5 (C-9a); 139.7 (C-1″); 136.8 (C-1′); 133.2 (C-2″); 132.3 (C-6″); 131.5 (C-2′,6′); 131.1 (C-4″); 129.2 (C-5″); 128.5 (C-3′,5′); 122.3 (C-4′); 118.8 (CN); 112.6 (C-3″); 90.2 (C-12′); 82.4 (C-11′); 61.3 (C-11); 50.6 (C-2); 49.3 (C-6); 48.5 (C-8); 46.9

(C-1); 45.2 (C-10); 32.2 (C-13′); 26.7 (C-9). HRMS: *m/z* calc. for $[C_{27}H_{27}N_6O]^+$: 451.2241 $[M+H]^+$; found: 451.2234; mass error: 1.52 ppm

4-(4-(3-Aminoprop-1-yn-1-yl)benzyl)-7-(4-(5-ferrocenyl-1*H*-1,2,3-triazol-1-yl)benzyl)-2,4,6,7,8,9-hexahydroimidazo[1,2-*a*]pyrido[3,4-*e*]pyrimidin-5(1H)-one (17b):

^{1}H-NMR ($CDCl_3$): 7.79 (s, 1H, H-12″); 7.45 (d, *J*=8.6 Hz, 2H, H-2″,6″); 7.37 (d, *J*=8.1 Hz, 2H, H-2′,6′); 7.35 (d, *J*=8.6 Hz, 2H, H-3″,5″); 7.30 (d, *J*=8.1 Hz, 2H, H-3′,5′); 5.01 (s, 2H, H-10); 4.23 (t, *J*=1.6 Hz, 2H, H-14″,17″); 4.18 (t, *J*=1.6 Hz, 2H, H-15″,16″); 4.05 (s, 5H, η^5-$C_5\underline{H}_5$); 3.87 (br ~s, 4H, H-1,2); 3.72 (s, 2H, H-11); 3.60 (br s, 2H, H-13′); 3.31 (br ~s, 2H, H-6); 2.67 (t, *J*=5.7 Hz, 2H, H-8); 2.46 (t, *J*=5.7 Hz, 2H, H-9); 1.74 (br s, 2H, NH_2). ^{13}C-NMR ($CDCl_3$): 161.4 (C-6); 152.9 (C-3a); 145.7 (C-9a); 139.8 (C-1″); 137.3 (C-11″); 136.9 (C-1′); 135.9 (C-4″); 132.4 (C-12″); 131.5 (C-3′,5′); 129.6 (C-2″,6″); 128.5 (C-2′,6′); 126.0 (C-3″,5″); 122.0 (C-4′); 101.6 (C-5a); 90.1 (C-12′); 82.4 (C-11′); 70.5 (C-13″); 69.9 (η^5-$\underline{C}_5H_5$); 69.4 (C-15″,16″); 68.4 (C-14″,17″); 61.6 (C-11); 50.6 (C-2); 49.6 (C-6); 48.3 (C-8); 46.9 (C-1); 45.1 (C-10); 32.2 (C-13′); 26.7 (C-9). HRMS: *m/z* calc. for $[C_{38}H_{37}FeN_8O]^+$: 677.2434 $[M+H]^+$; found: 677.2402. mass error: 4.76 ppm.

4-(4-(3-Aminoprop-1-yn-1-yl)benzyl)-7-(4-(4-ferrocenyl-1H-1,2,3-triazol-1-yl)benzyl)-2,4,6,7,8,9-hexahydroimidazo[1,2-*a*]pyrido[3,4-*e*]pyrimidin-5(1*H*)-one (17c):

^{1}H-NMR ($CDCl_3$): 7.87 (s, 1H, H-11″); 7.72 (d, *J*=8.3 Hz, 2H, H-3″,5″); 7.50 (d, *J*=8.3 Hz, 2H, H-2″,6″); 7.37 (d, *J*=8.1 Hz, 2H, H-2′,6′); 7.32 (d, *J*=8.1 Hz, 2H, H-3′,5′); 5.02 (s, 2H, H-10); 4.79 (t, *J*=1.8 Hz, 2H, H-14″,17″); 4.34 (t, *J*=1.8 Hz, 2H, H-15″,16″); 4.12 (s, 5H, η^5-$C_5\underline{H}_5$); 3.90 (br ~s, 4H, H-1,2); 3.72 (s, 2H, H-11); 3.65 (br s, 2H, H-13′); 3.30 (br ~s, 2H, H-6); 2.70 (t, *J*=5.7 Hz, 2H, H-8); 2.49 (t, *J*=5.7 Hz, 2H, H-9); 1.77 (br s, 2H, NH_2). ^{13}C-NMR ($CDCl_3$): 161.4 (C-6); 152.9 (C-3a); 147.6 (C-12″); 145.6 (C-9a); 138.5 (C-1″); 136.8 (C-1′); 136.3 (C-4″); 131.5 (C-3′,5′); 130.2 (C-2″,6″); 128.5 (C-2′,6′); 122.3 (C-4′); 120.4 (C-3″,5″); 116.6 (C-11″); 101.8 (C-5a); 90.1 (C-12′); 82.4 (C-11′); 74.9 (C-13″); 69.7 (η^5-$\underline{C}_5H_5$); 68.9 (C-15″,16″); 66.8 (C-14″,17″); 61.6 (C-11); 50.6 (C-2); 49.4 (C-6); 48.4 (C-8); 46.9 (C-1); 45.2 (C-10); 32.2 (C-13′); 26.8 (C-9). HRMS: *m/z* calc. for $[C_{38}H_{37}FeN_8O]^+$: 677.2434 $[M+H]^+$; found: 677.2404; mass error: 4.46 ppm.

4-(4-(3-Aminoprop-1-yn-1-yl)benzyl)-7-(4-(4-phenyl-1*H*-1,2,3-triazol-1-yl)benzyl)-2,4,6,7,8,9-hexahydroimidazo[1,2-*a*]pyrido[3,4-*e*]pyrimidin-5(1*H*)-one (17d):

^{1}H-NMR ($CDCl_3$): 8.18 (s, 1H, H-11″); 7.91 (dd, J77.8 Hz and 2.1 Hz, 2H, H-14″,18″); 7.73 (d, *J*=8.3 Hz, 2H, H-3″,5″); 7.50 (d, *J*=8.3 Hz, 2H, H-2″,6″); 7.46 (t, *J*=7.8 Hz, 2H, H-15″17″); 7.37 and 7.36 (partly overlapping d (*J*=8.2 Hz) and tt (*J*=7.8 Hz and 2.1 Hz), 3H, H-2′,6′ and H-16″); 7.31 (d, *J*=8.2 Hz, 2H, H-3′,5′); 5.02 (s, 2H, H-10); 3.89 (br ~s, 4H, H-1,2); 3.71 (s, 2H, H-11); 3.62 (br s, 2H, H-13′); 3.30 (br ~s, 2H, H-6); 2.71 (t, *J*=5.7 Hz, 2H, H-8); 2.48 (t, *J*=5.7 Hz, 2H, H-9); 1.65 (br s, 2H, NH_2). ^{13}C-NMR ($CDCl_3$): 161.5 (C-6); 153.0 (C-3a); 148.5 (C-12″); 145.6 (C-9a); 138.9 (C-1″); 136.8 (C-1′); 136.2 (C-4″); 131.5 (C-3′,5′); 130.3 (C-2″,6″); 129.9 (C-13″); 128.9 (C-15″,17″); 128.6 (C-2′,6′); 128.5 (C-16″); 125.9 (C-14″,18″); 122.3 (C-4′); 120.7 (C-3″,5″); 117.7 (C-11″); 101.8 (C-5a); 90.1 (C-12′); 82.5 (C-11′); 61.6 (C-11); 50.6 (C-2); 49.4 (C-6); 48.4 (C-8); 46.9 (C-1); 45.2 (C-10); 32.2 (C-13′); 27.0 (C-9). HRMS: *m/z* calc. for $[C_{34}H_{33}N_8O]^+$: 569.2772 $[M+H]^+$; found: 569.2765; mass error: 1.20 ppm.

7-(3-cyanobenzyl)-4-(4-ethynylbenzyl)-2,4,6,7,8,9-hexahydroimidazo[1,2-*a*]pyrido[3,4-*e*]pyrimidin-5(1*H*)-one (**18**):

^{1}H-NMR ($CDCl_3$): 7.67 (br ~t, *J*~2 Hz, 1H, H-2″); 7.52-7.57 (overlapping m′s, 2H, H-4″ and H-6″); 7.42 (t, *J*=7.8 Hz, 1H, H-5″); 7.40 (br ~s 4H, H-2′,3′,5′,6′); 5.04 (s, 2H, H-10); 3.90 (s, 4H, H-1,2); 3.68 (s, 2H, H-11); 3.26 (br s, 2H, H-6); 3.03 (s, 1H, H-12′); 2.68, (t, *J*=5.6 Hz, 2H, H-8); 2.50 (t, 2H, *J*=5.6 Hz, H-9). ^{13}C-NMR ($CDCl_3$): 161.3 (C-5); 152.9 (C-3a); 145.6 (C-9a); 139.7 (C-1″); 137.7 (C-1′); 133.2 (C-2″); 132.3 (C-6″); 132.1 (C-3′,5′); 131.1 (C-4″); 129.3 (C-5″); 128.5 (C-2′,6′); 121.2 (C-4′); 118.8 (C̲N); 112.6 (C-3″); 101.5 (C-5a); 83.7 (C-11′); 77.06 (coalesced with the central line of the solvent signal, C-12′), 61.3 (C-11); 50.6 (C-2); 49.3 (C-6); 48.5 (C-8); 46.9 (C-1); 45.1 (C-10); 26.7 (C-9).

S.3. Copies of the ^{1}H- and ^{13}C-NMR spectra

^{1}H-NMR of 7bf

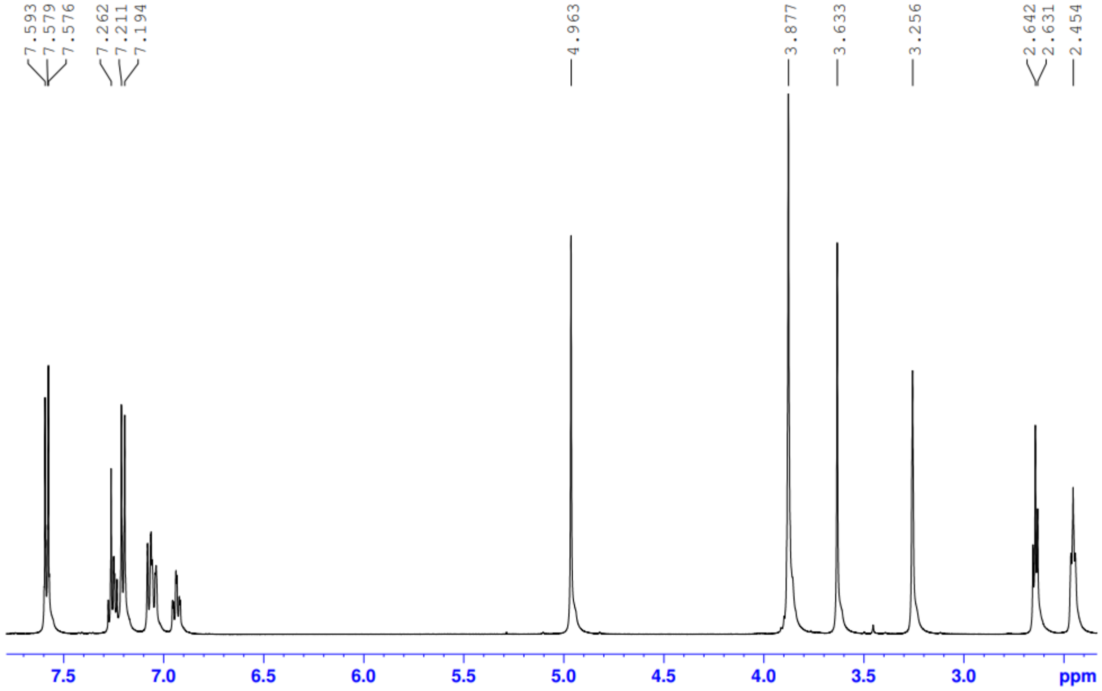

^{13}C-NMR of 7bf

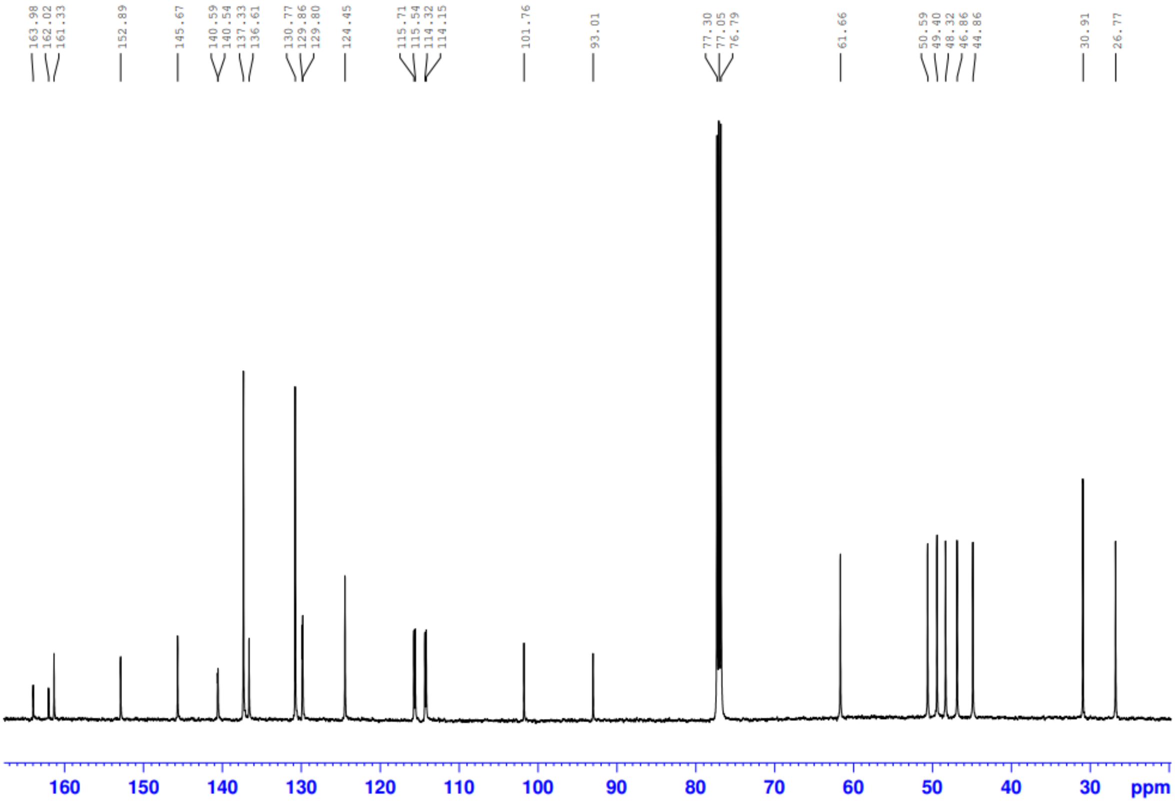

¹H-NMR of 7bh

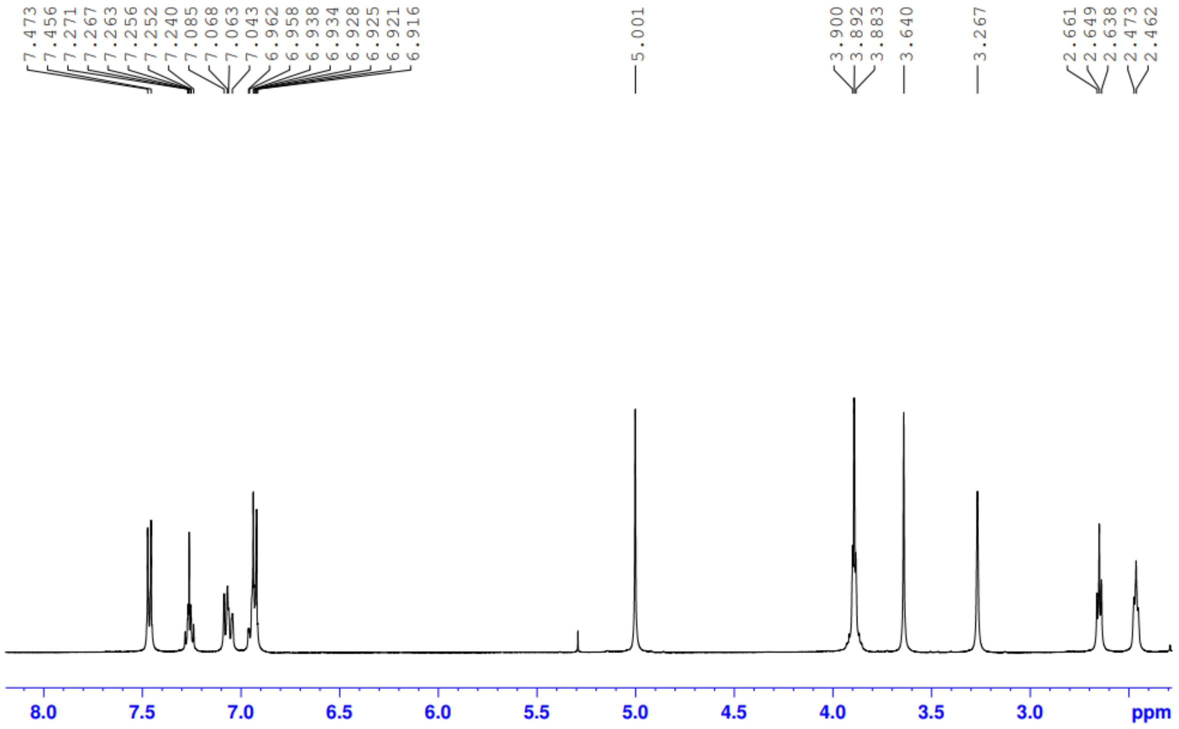

¹³C-NMR of 7bh

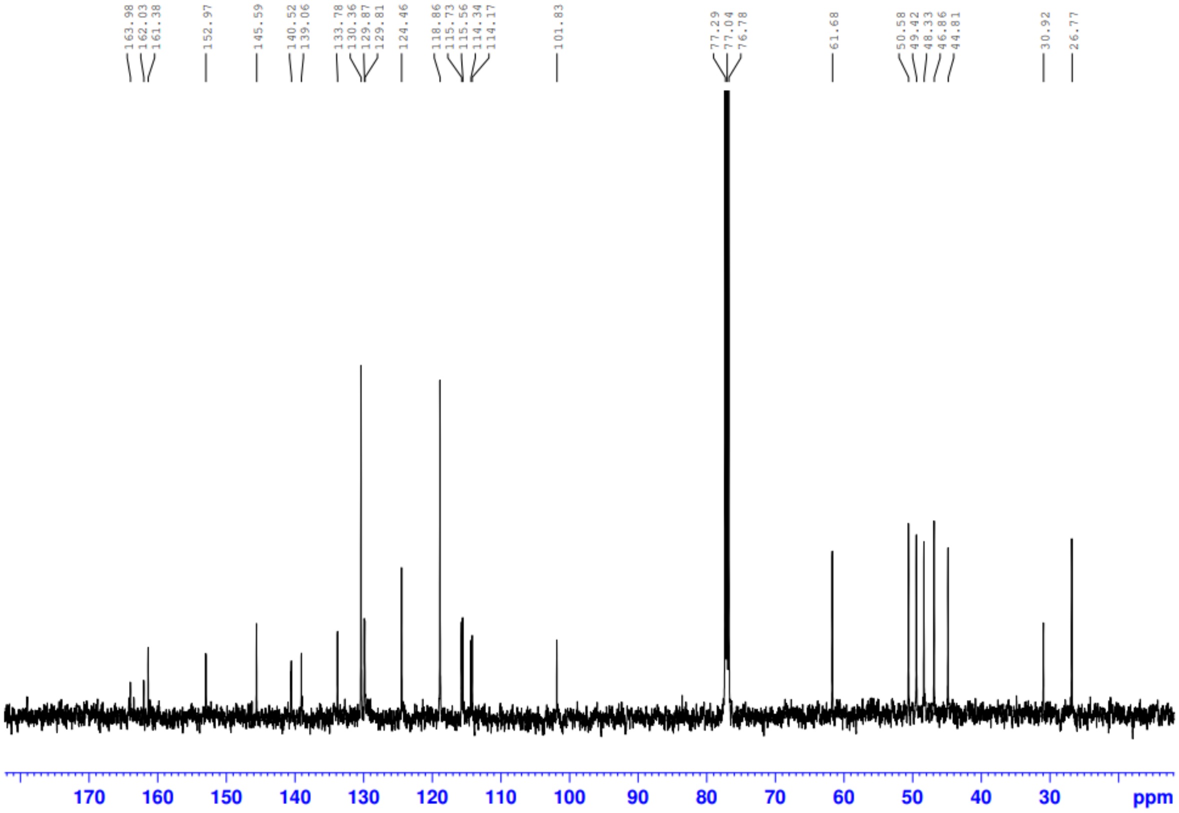

^{1}H-NMR of 7cf

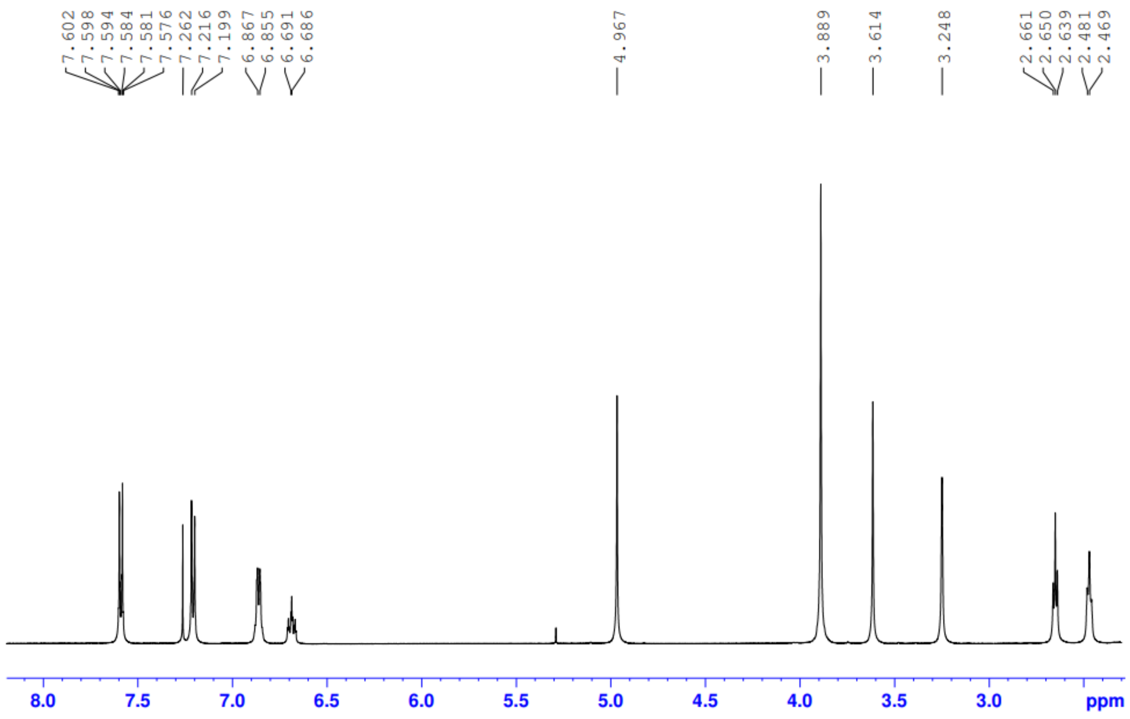

^{13}C-NMR of 7cf

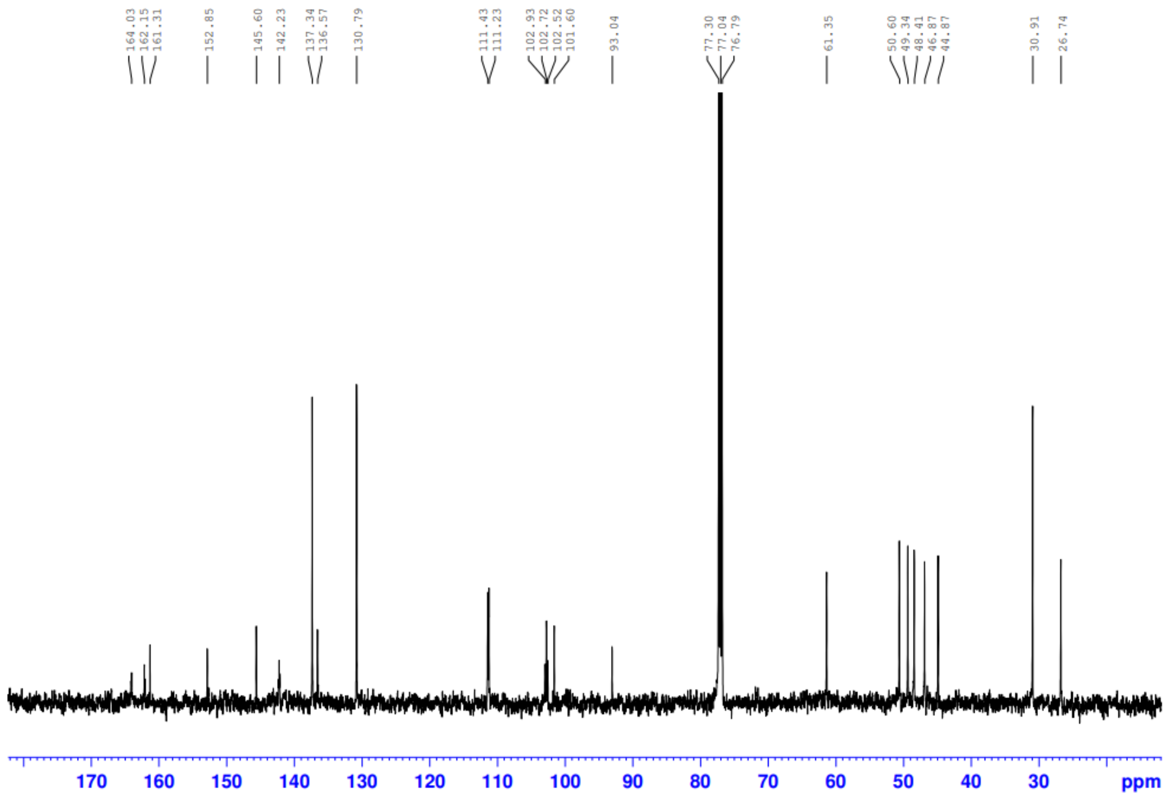

^{1}H-NMR of 7ch

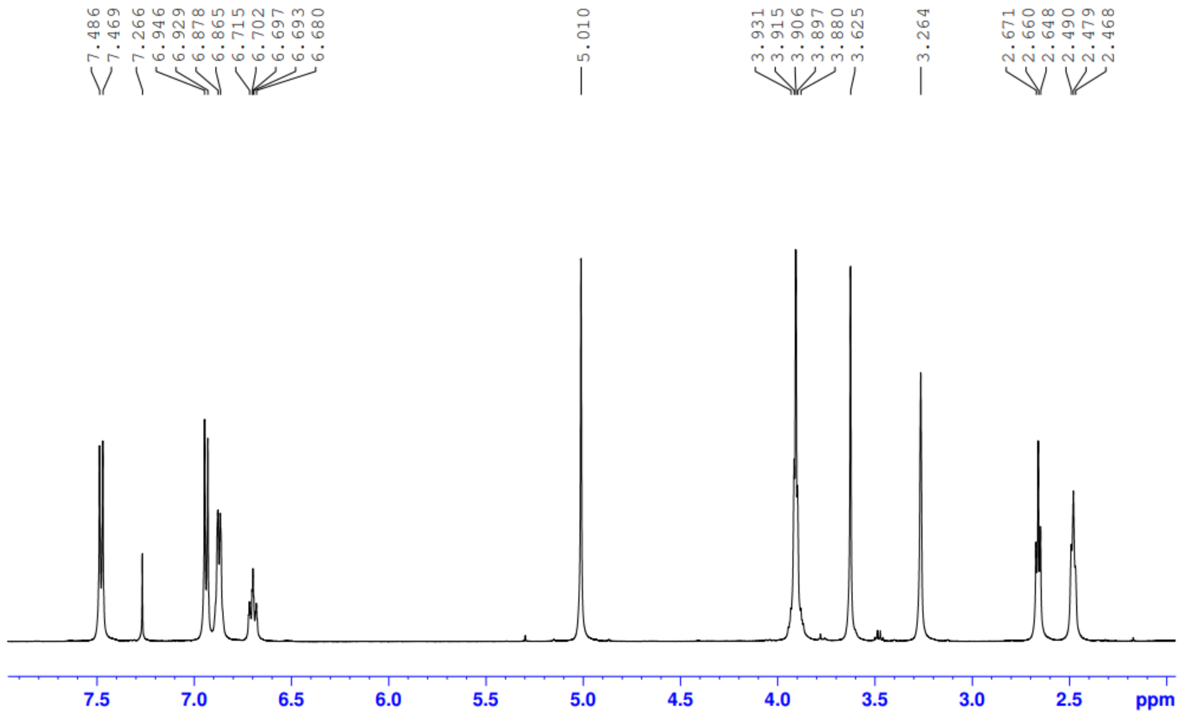

^{13}C-NMR of 7ch

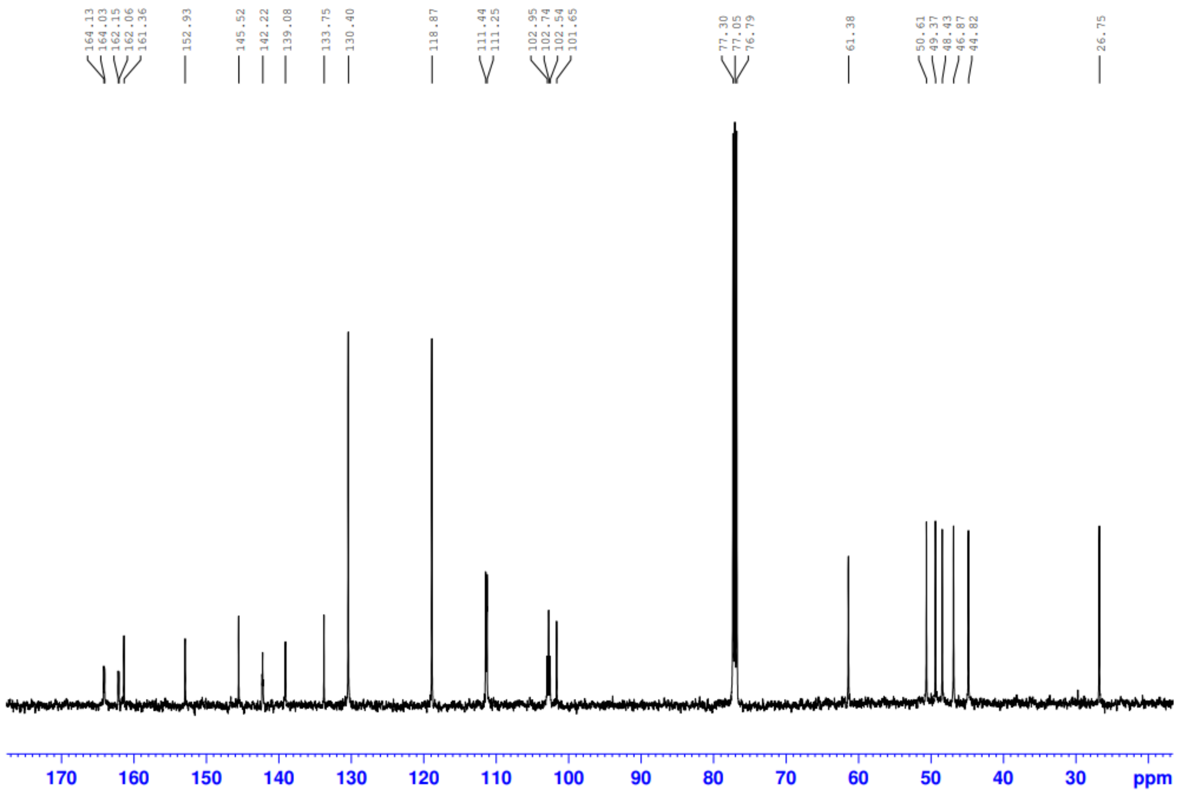

^{1}H-NMR of 7df

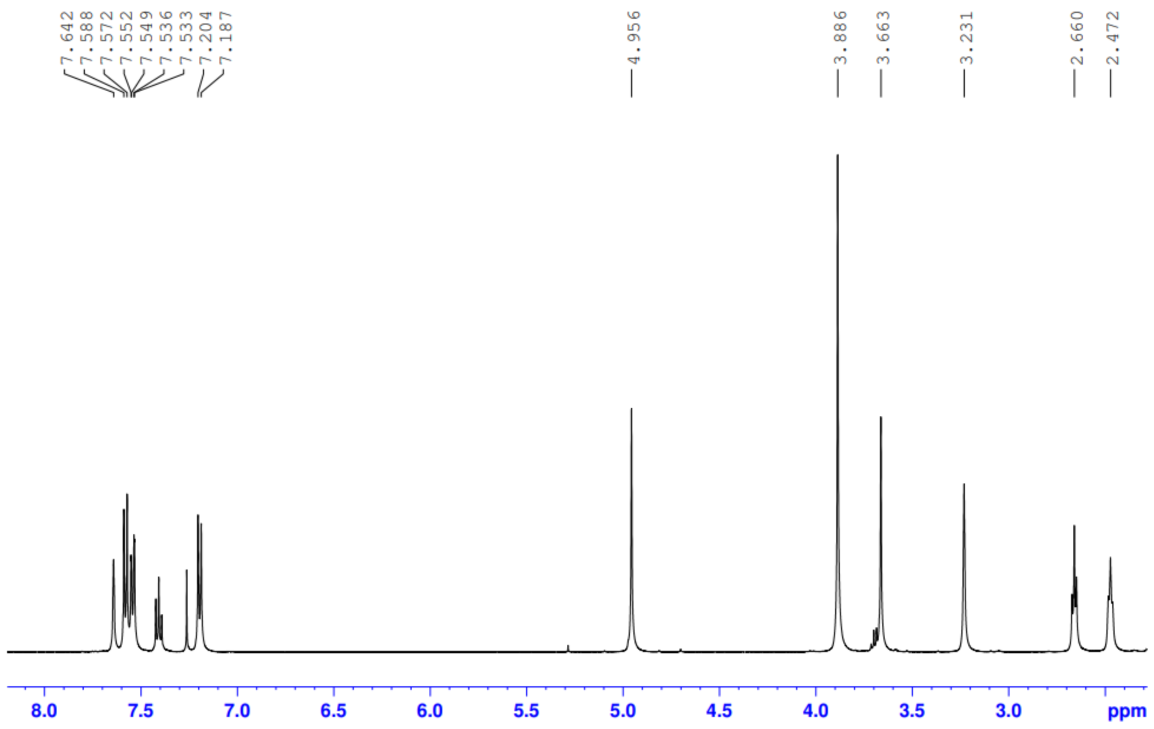

^{13}C-NMR of 7df

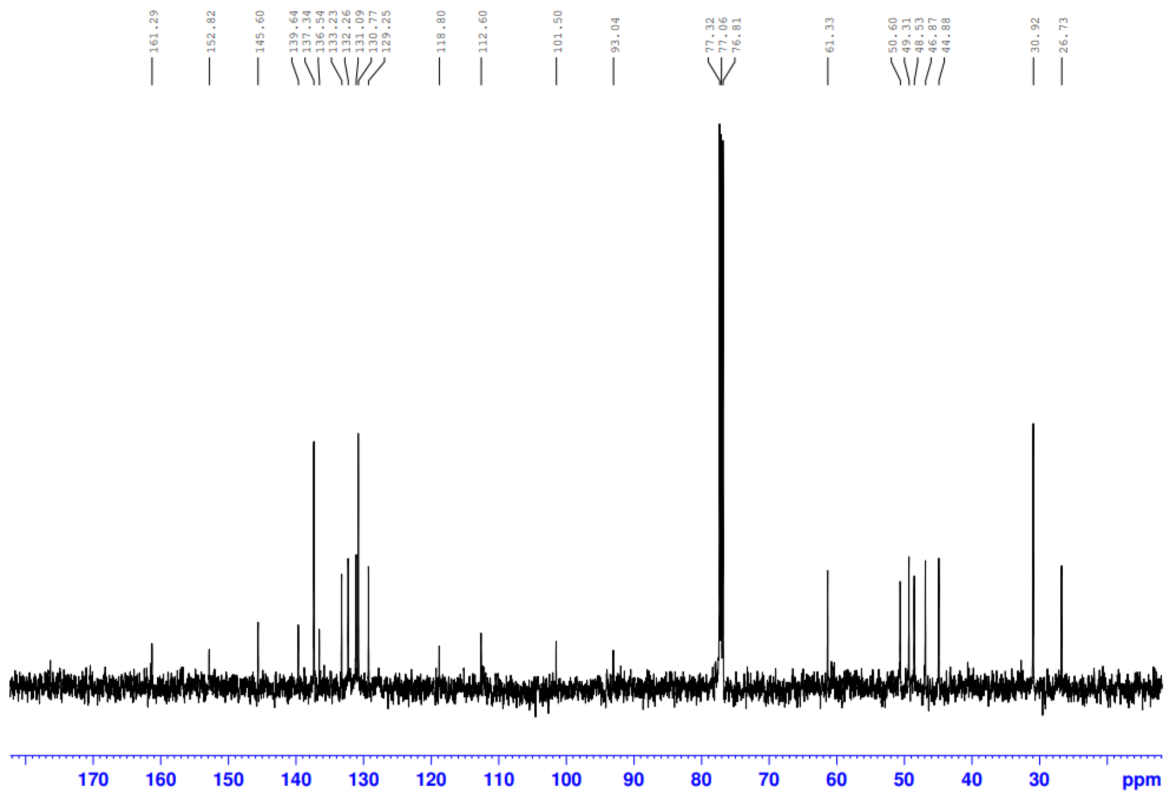

^{1}H-NMR of 7hf

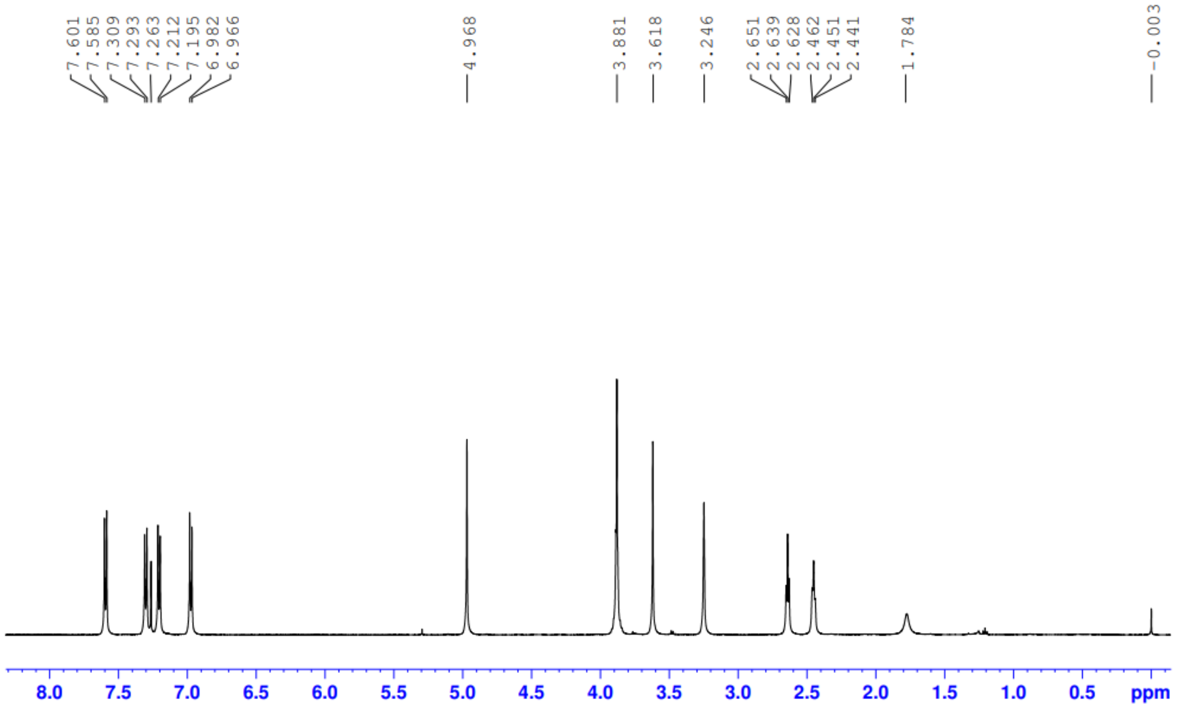

^{13}C-NMR of 7hf

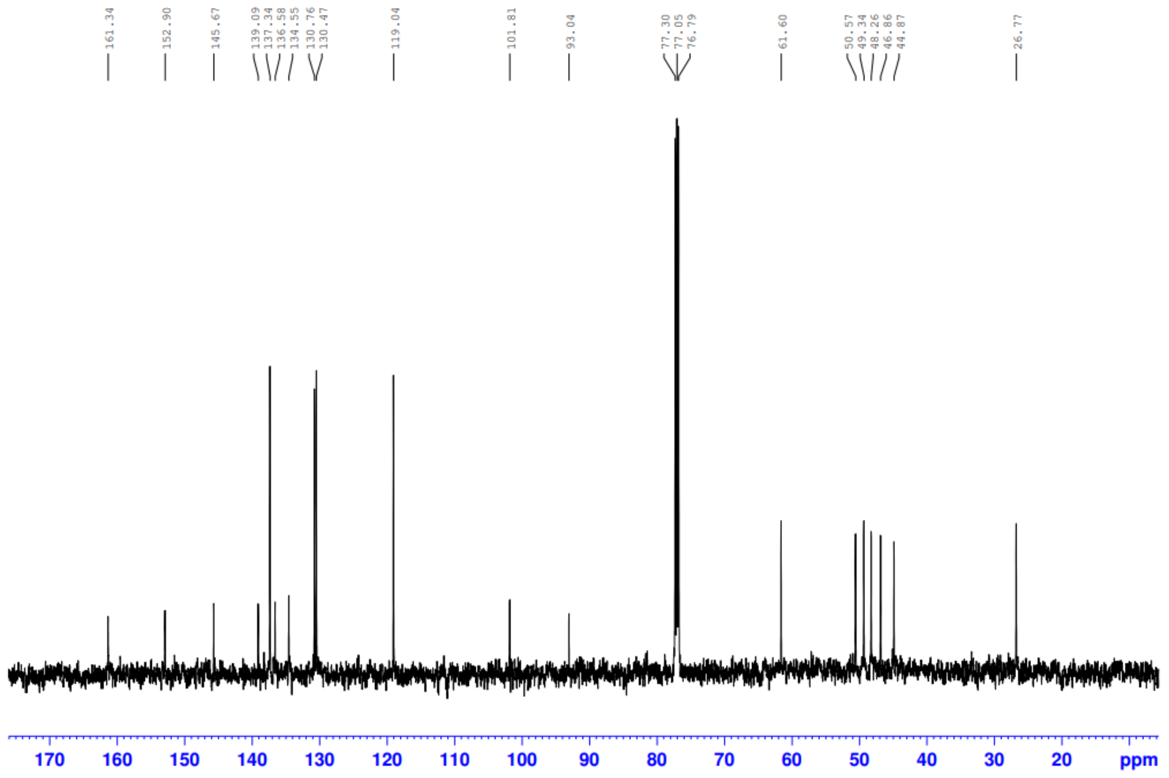

^{1}H-NMR of 7hg

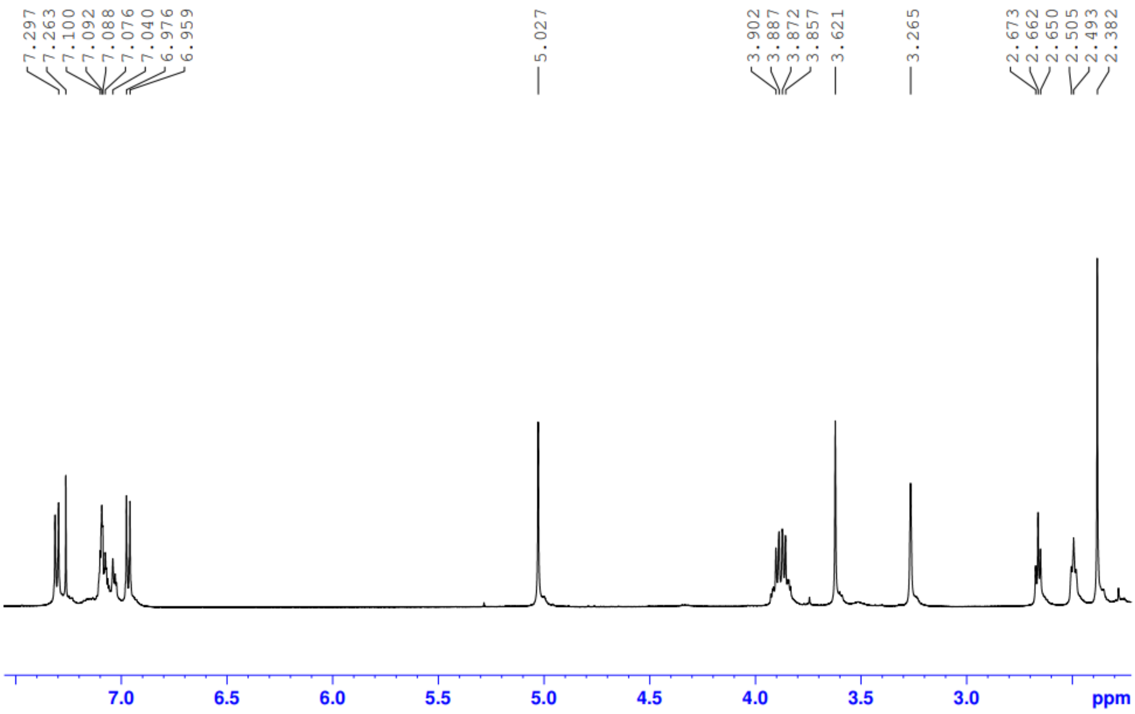

^{13}C-NMR of 7hg

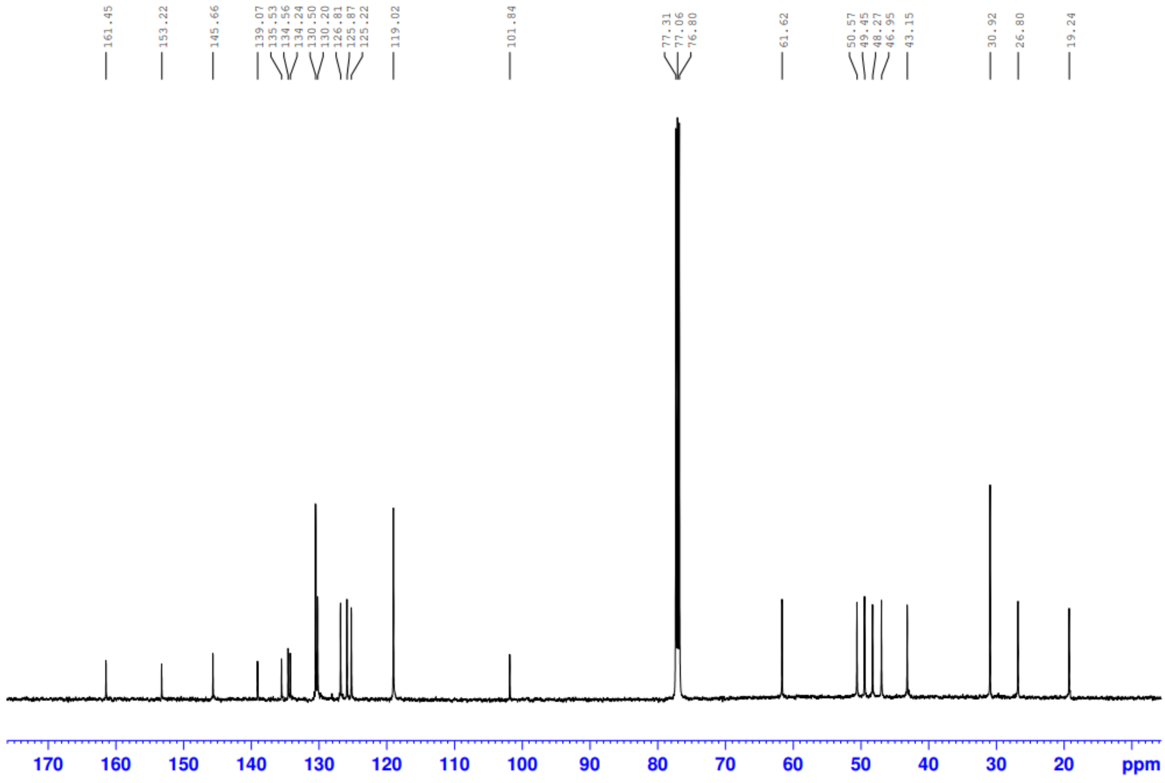

^{1}H-NMR of 9a

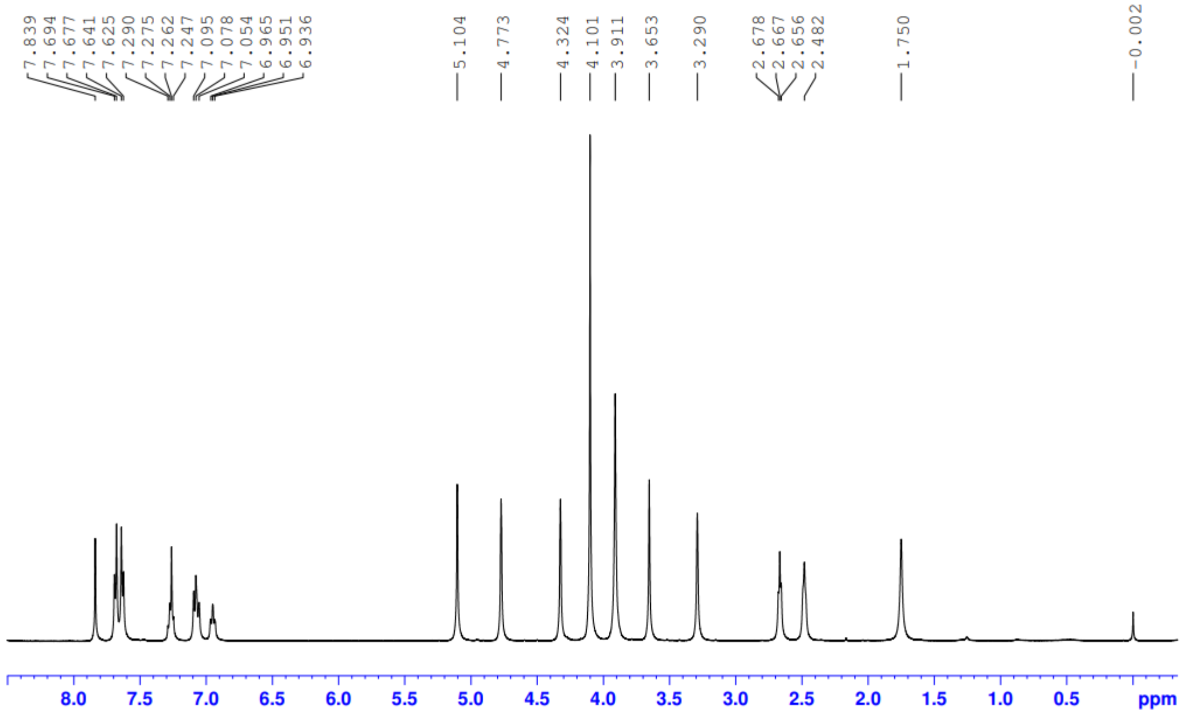

^{13}C-NMR of 9a

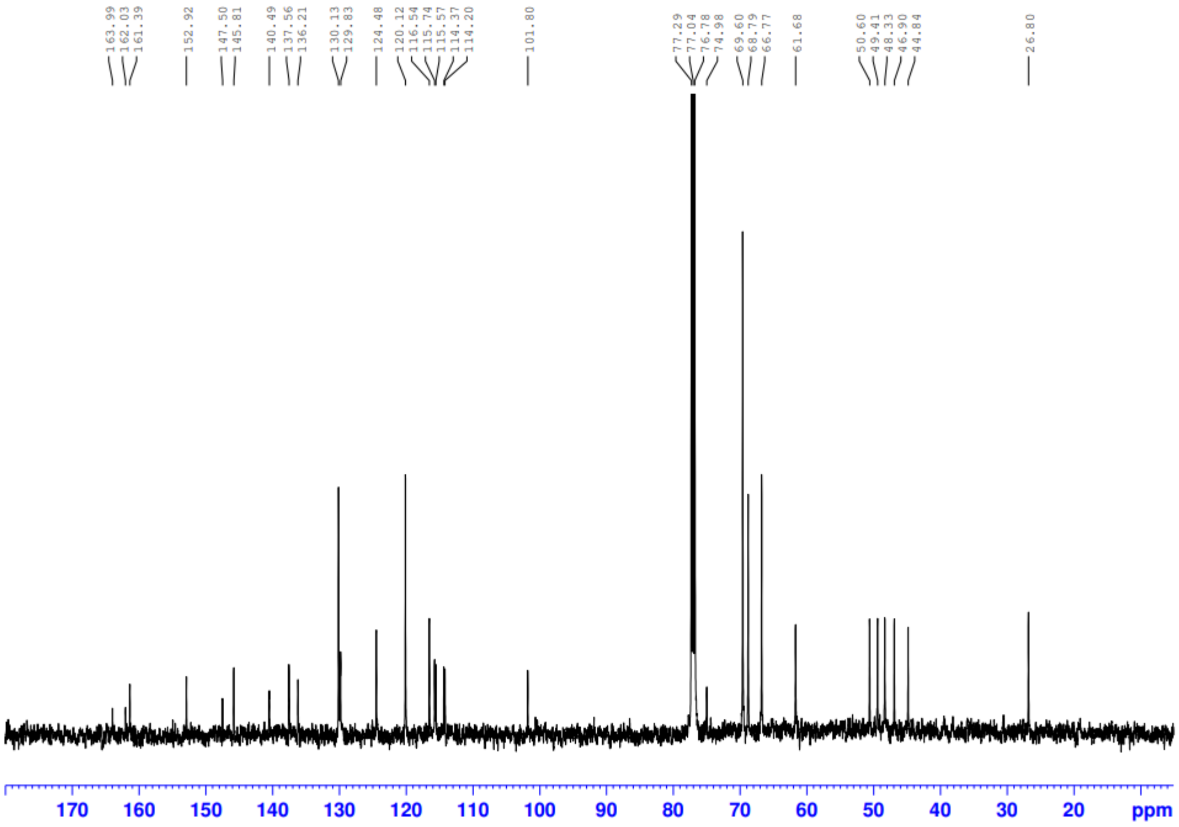

^{1}H-NMR of 9b

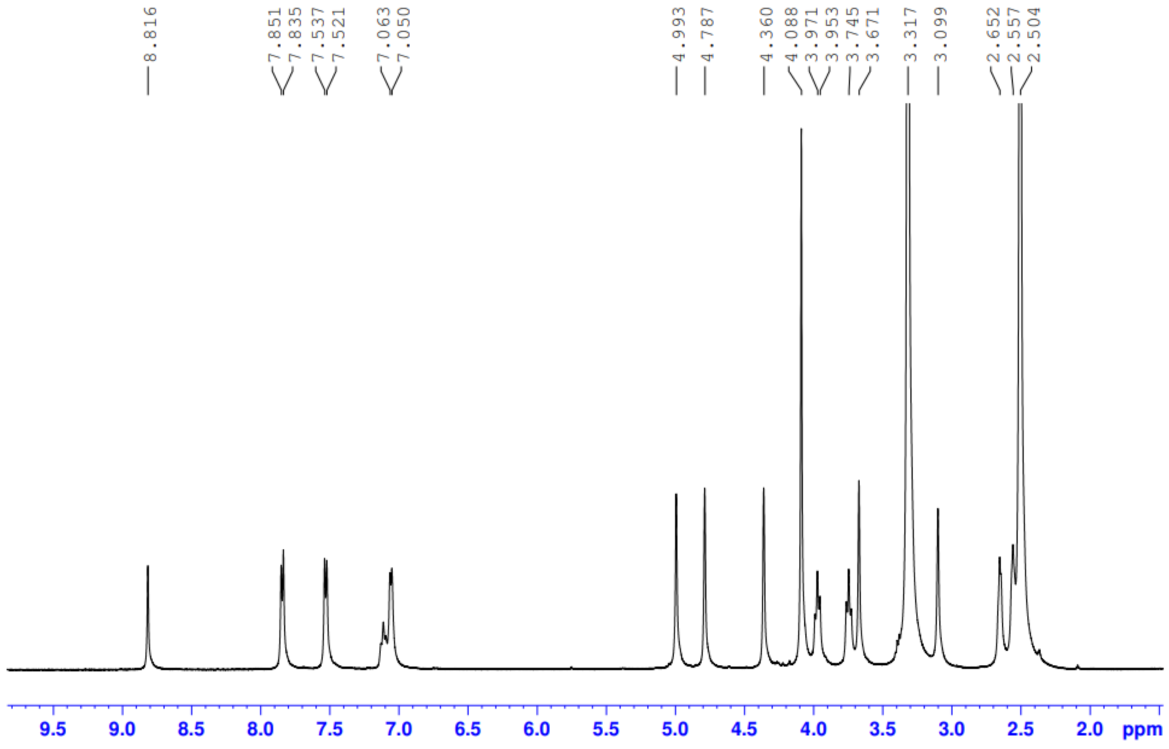

^{1}H-NMR of 10a

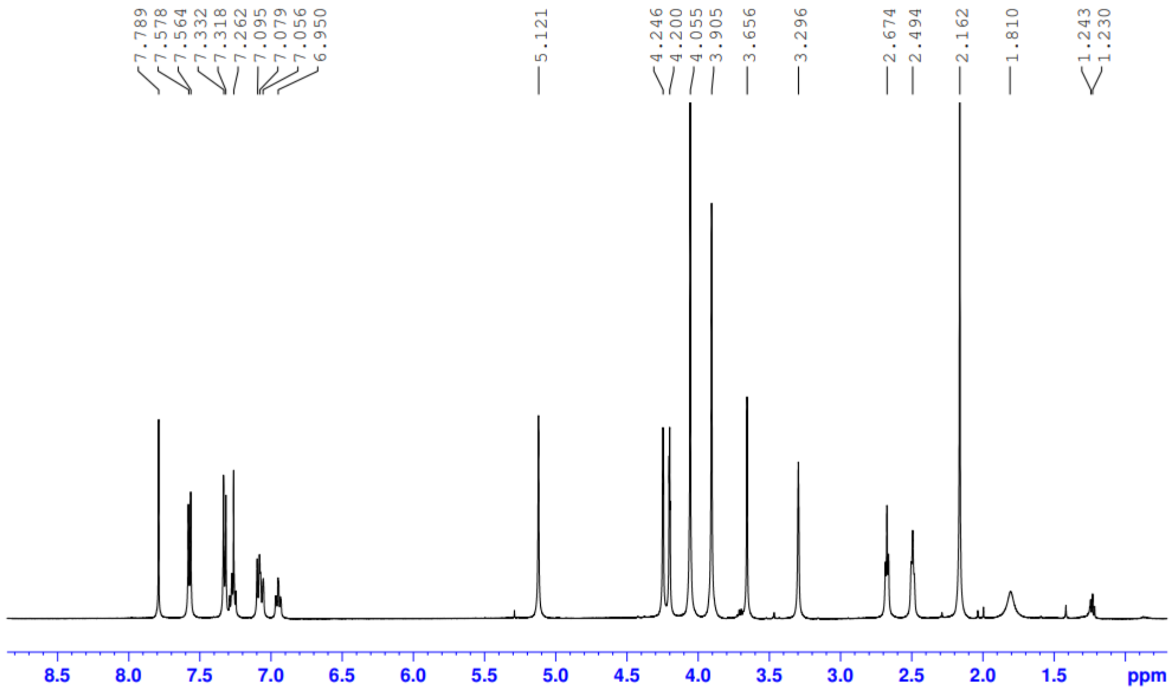

^{13}C-NMR of 10a

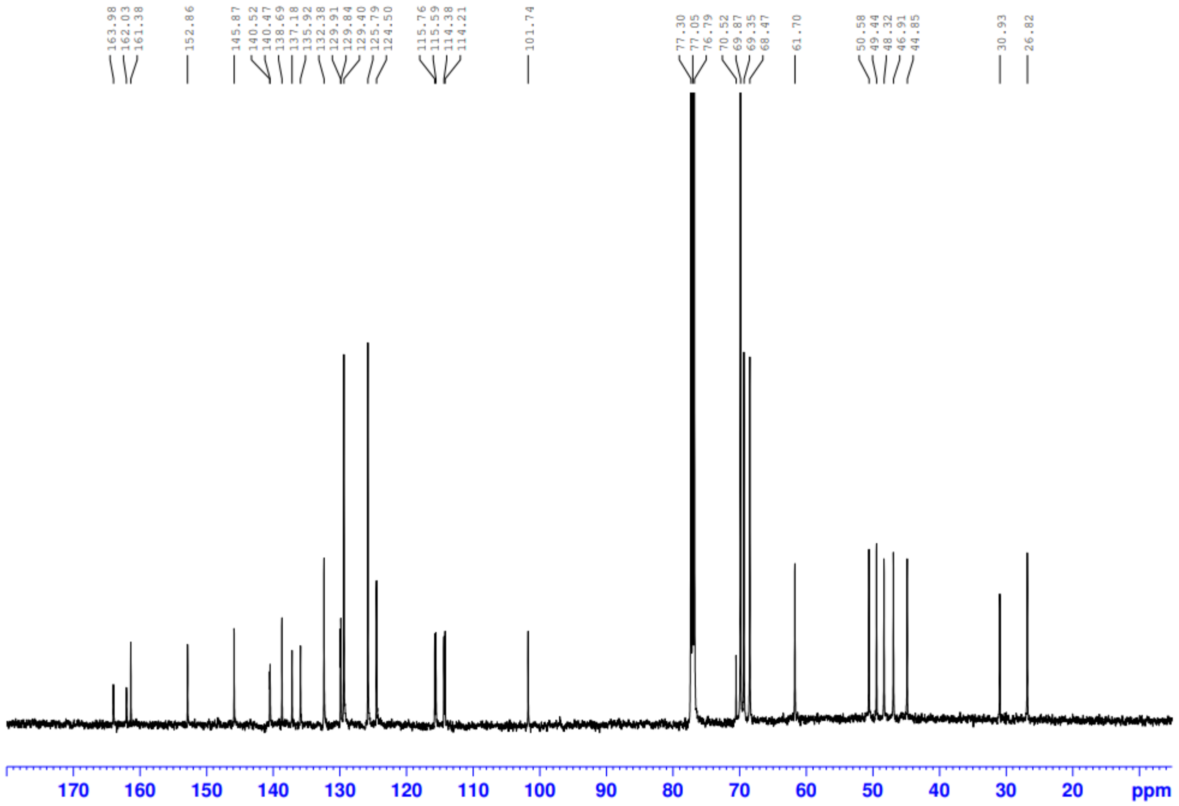

^{1}H-NMR of 10b

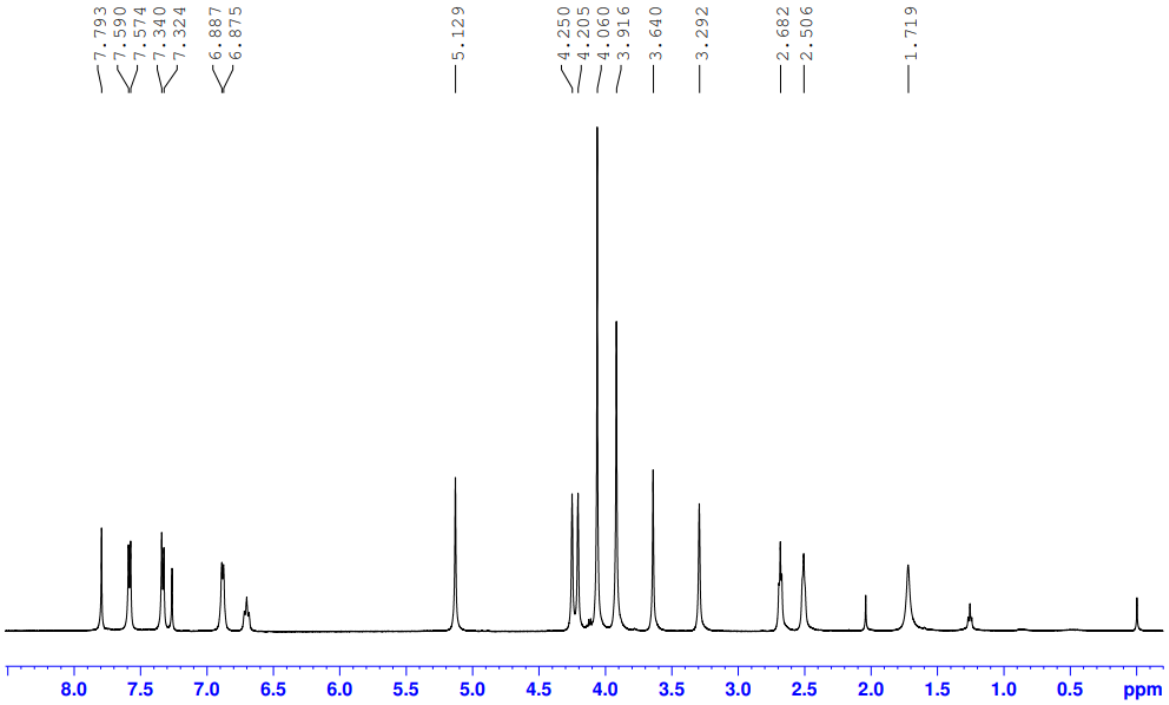

^{13}C-NMR of 10b

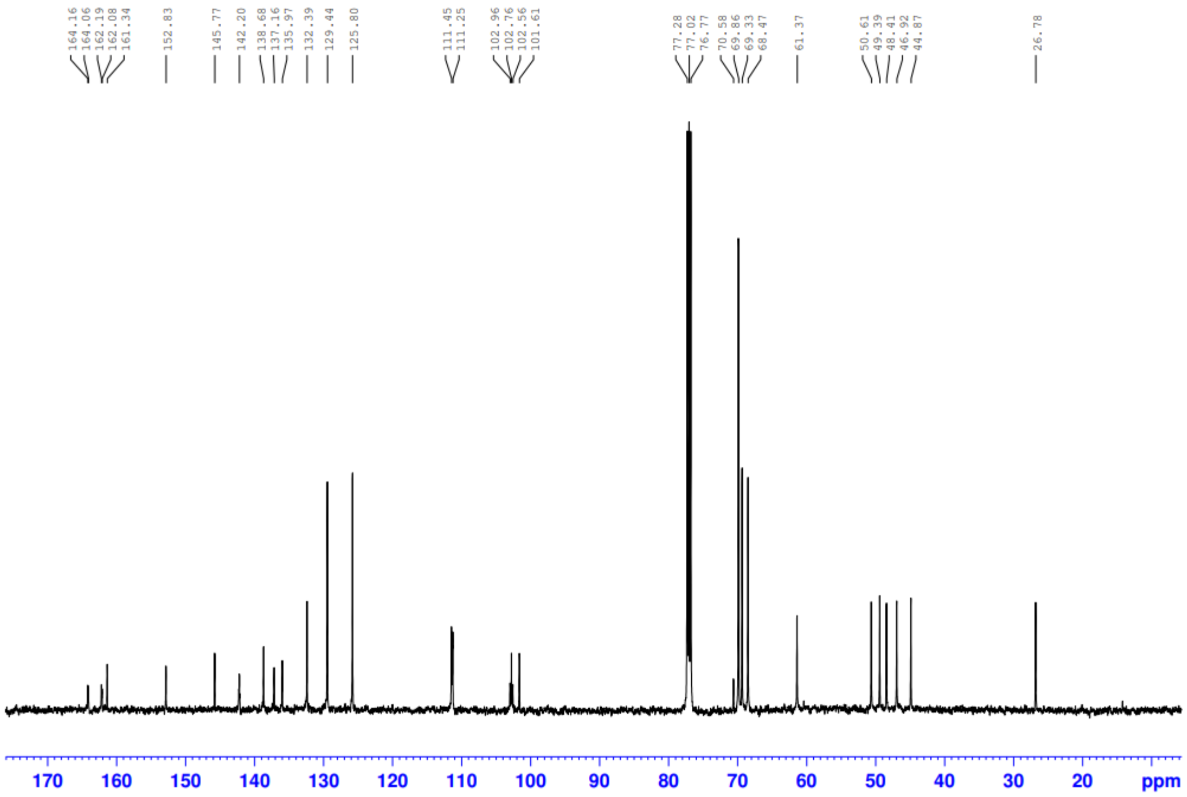

^{1}H-NMR of 11

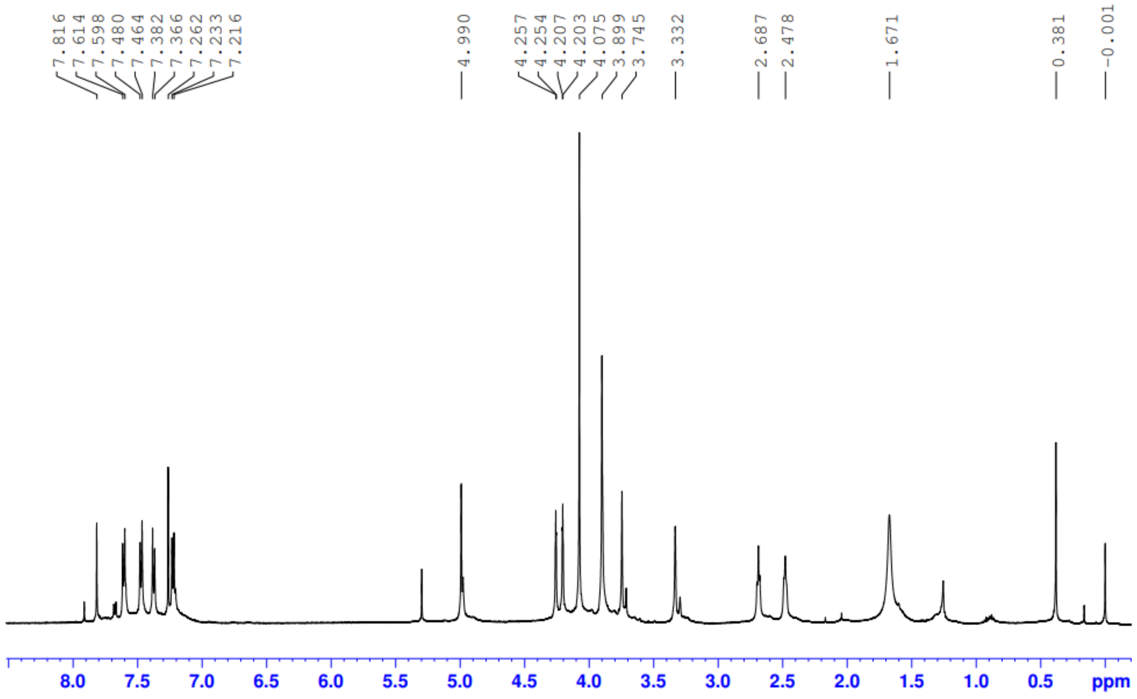

^{1}H-NMR of 12a

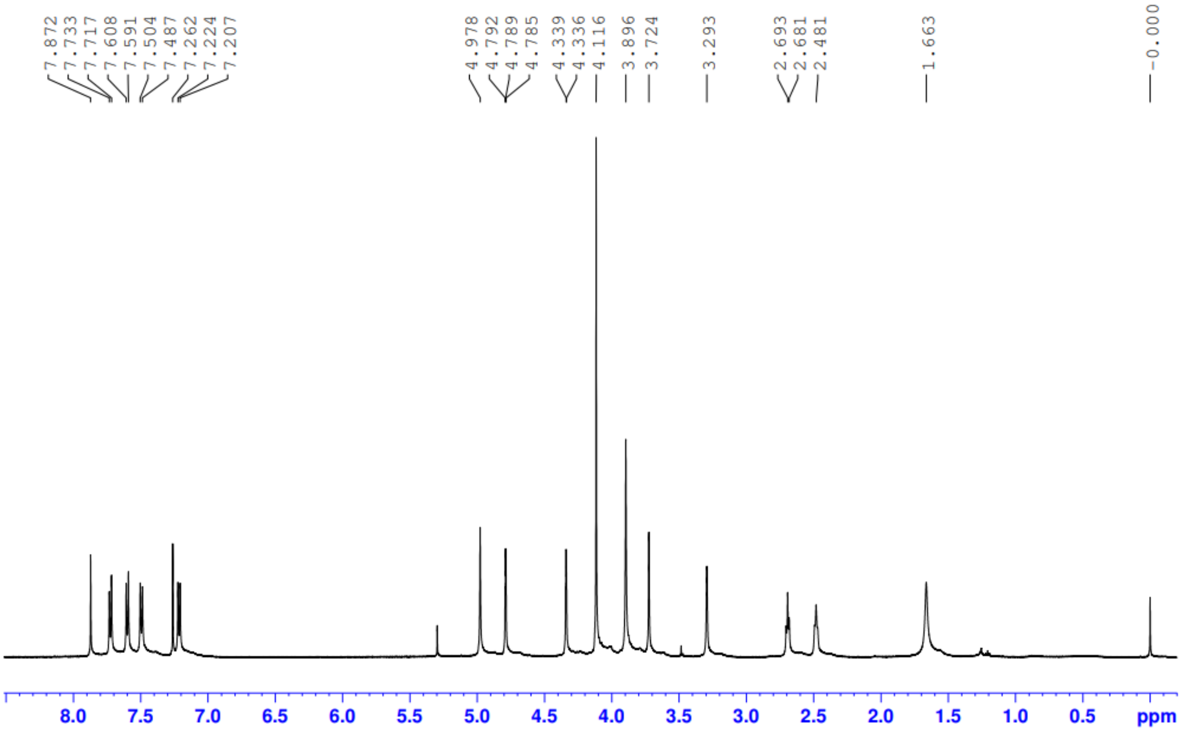

^{1}H-NMR of 12b

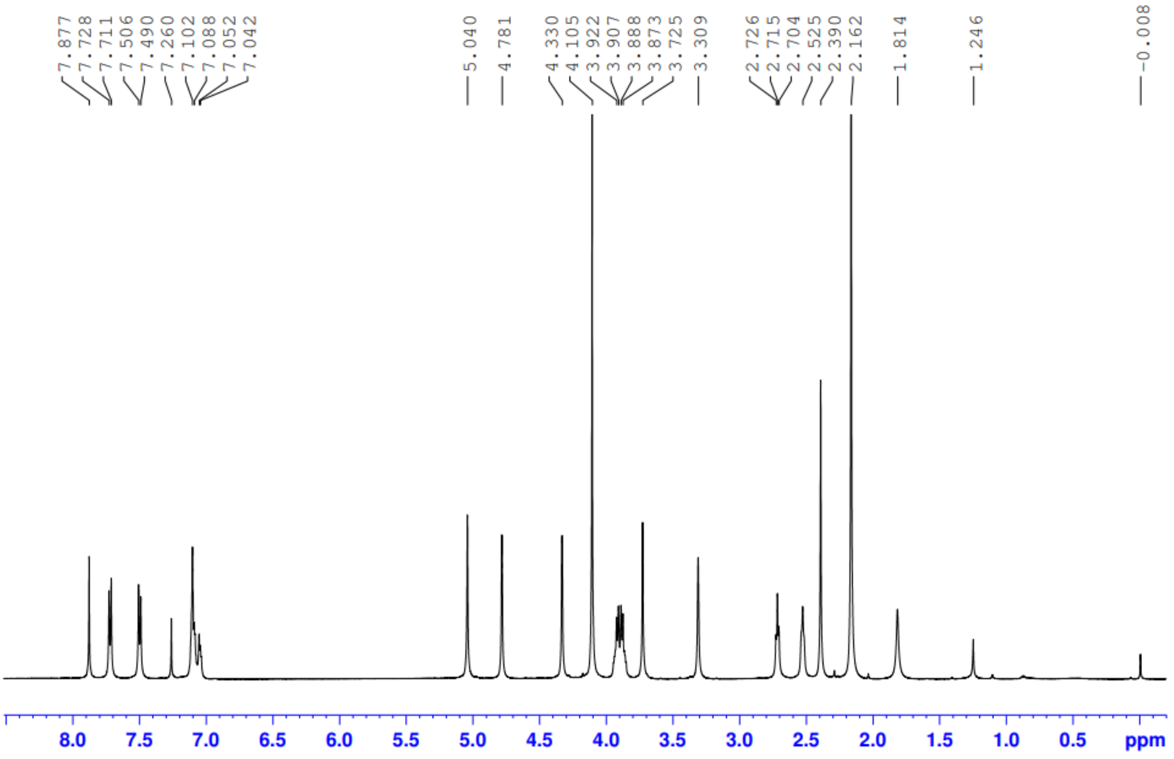

^{13}C-NMR of 12b

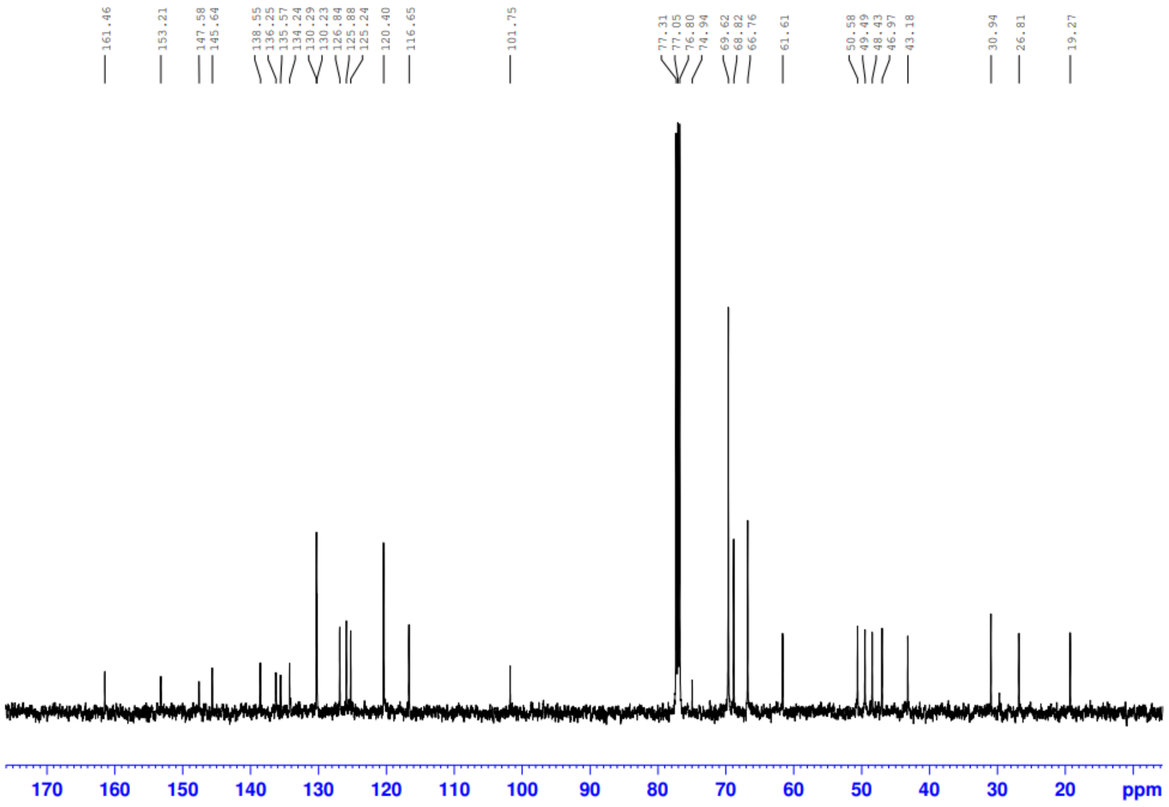

^{1}H-NMR of 13

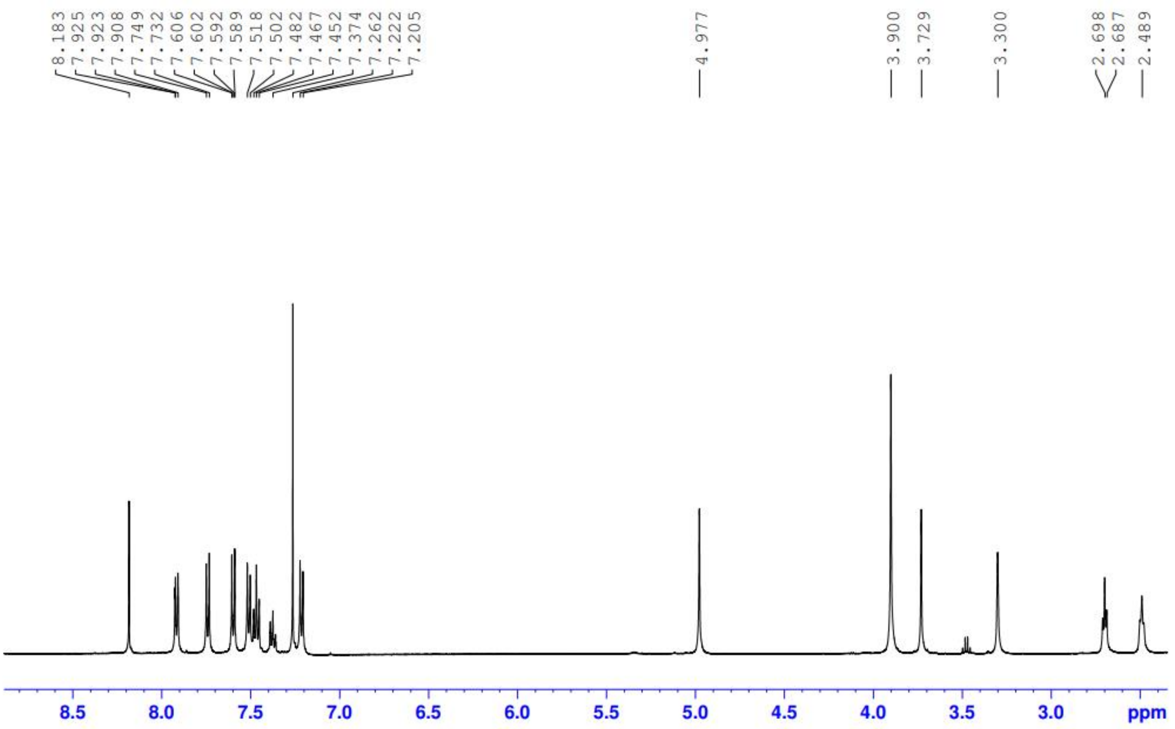

^{1}H-NMR of 16a

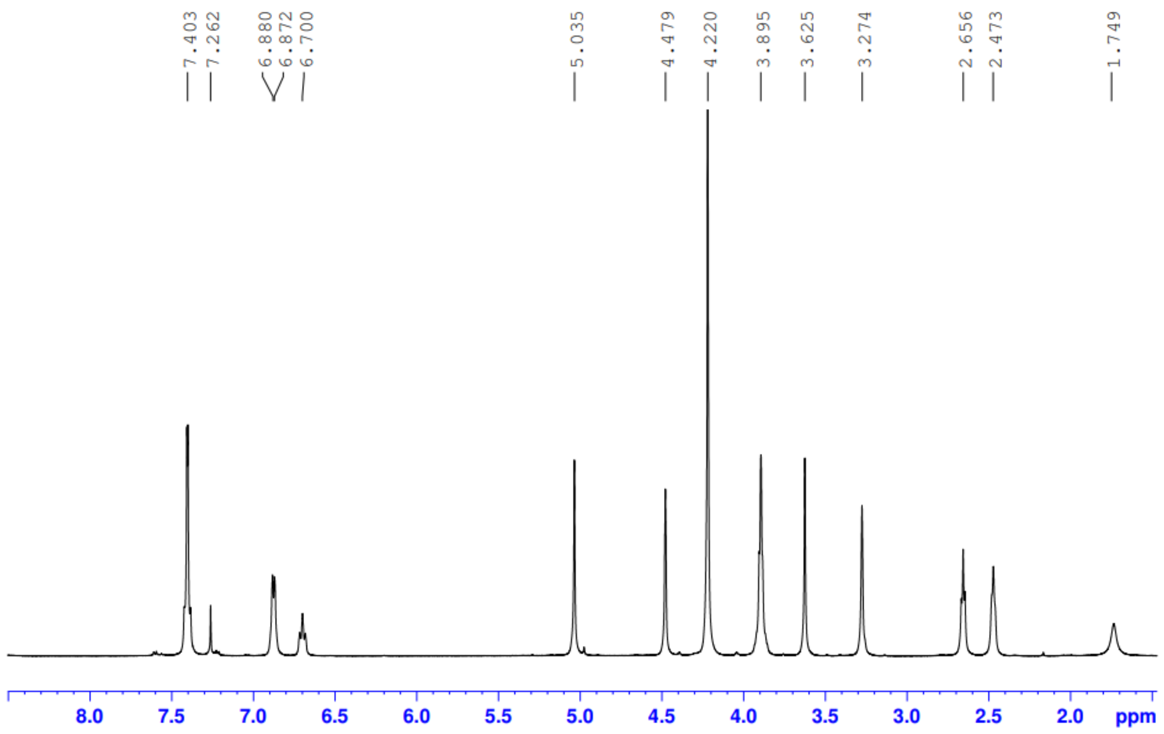

^{13}C-NMR of 16a

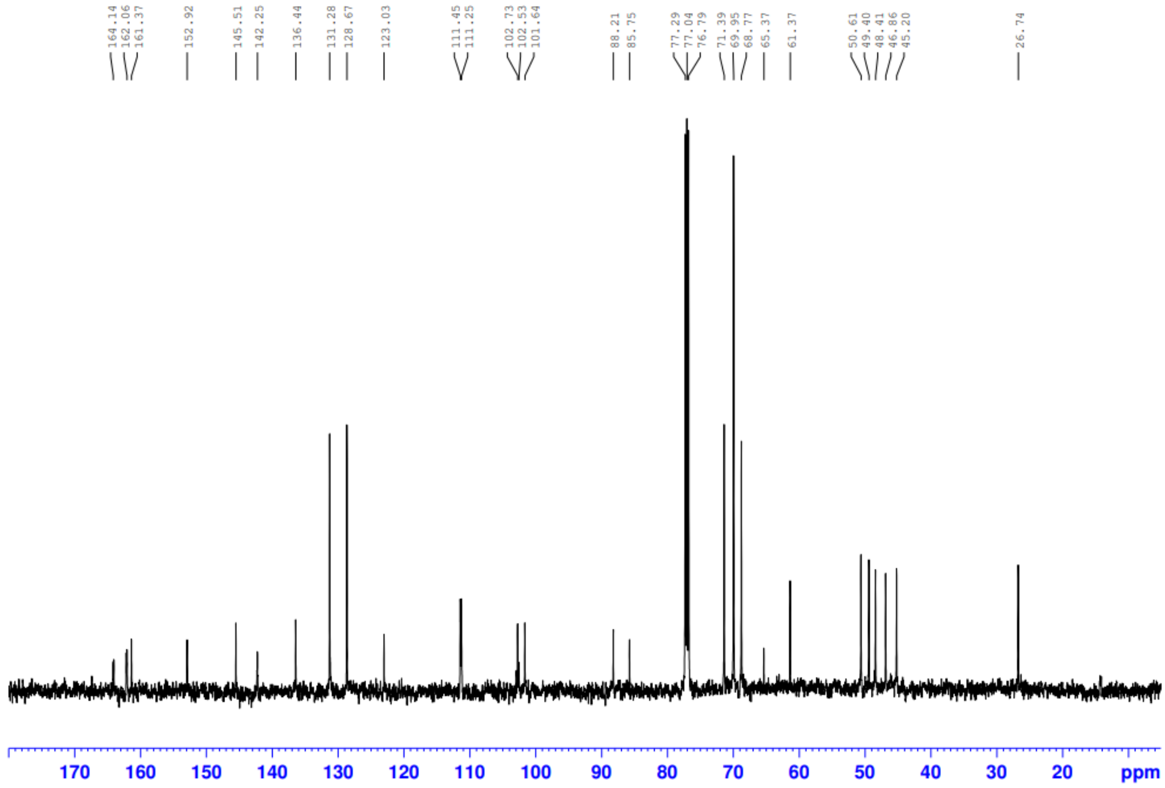

^{1}H-NMR of 16b

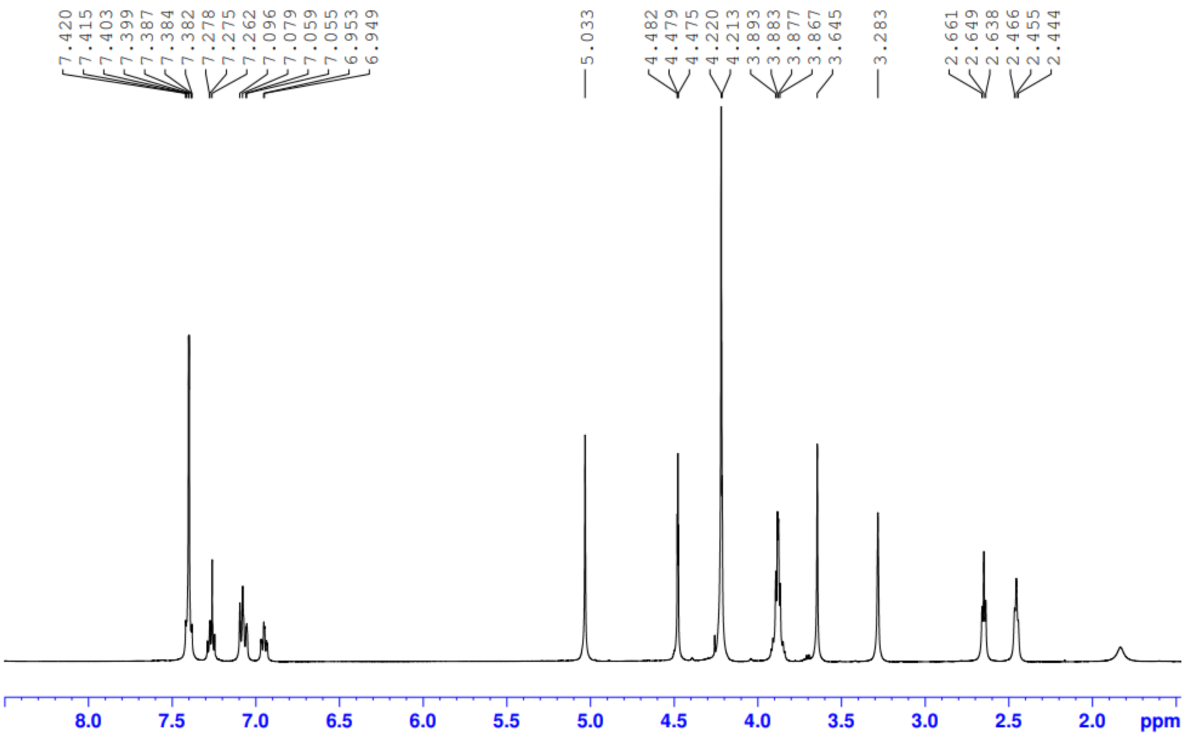

^{13}C-NMR of 16b

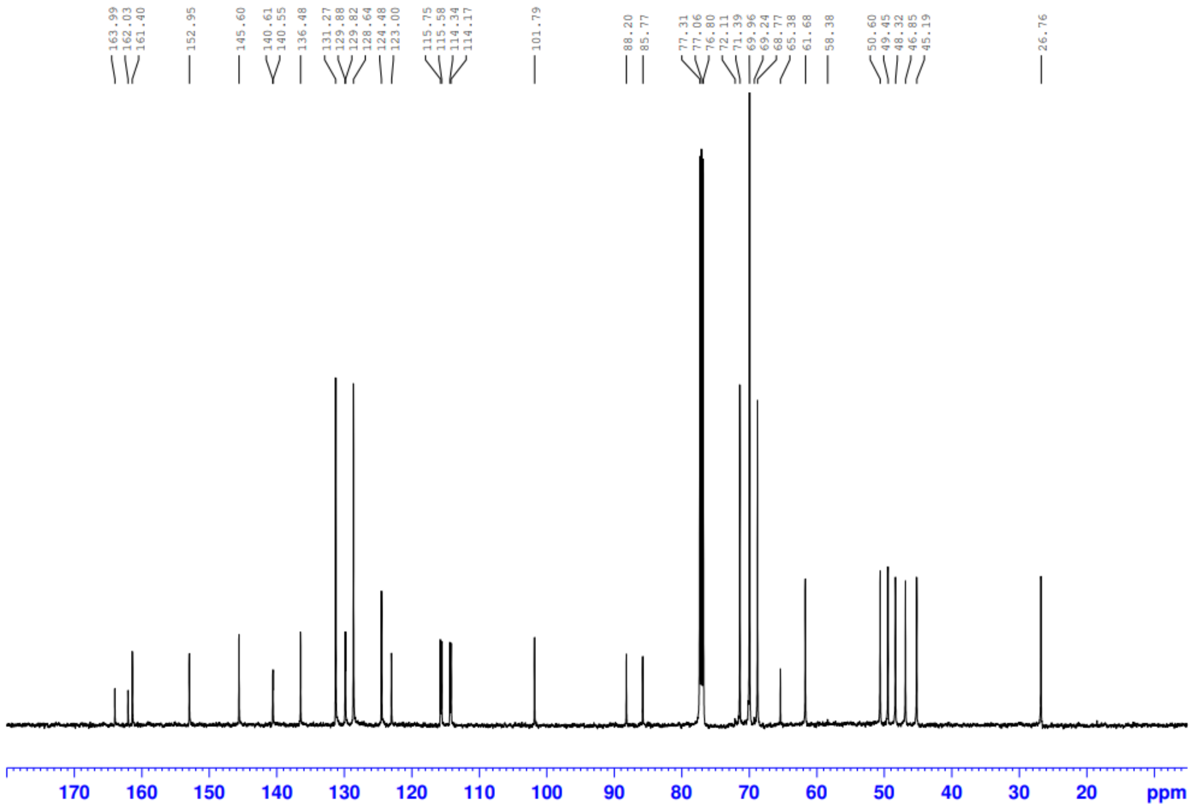

^{1}H-NMR of 16c

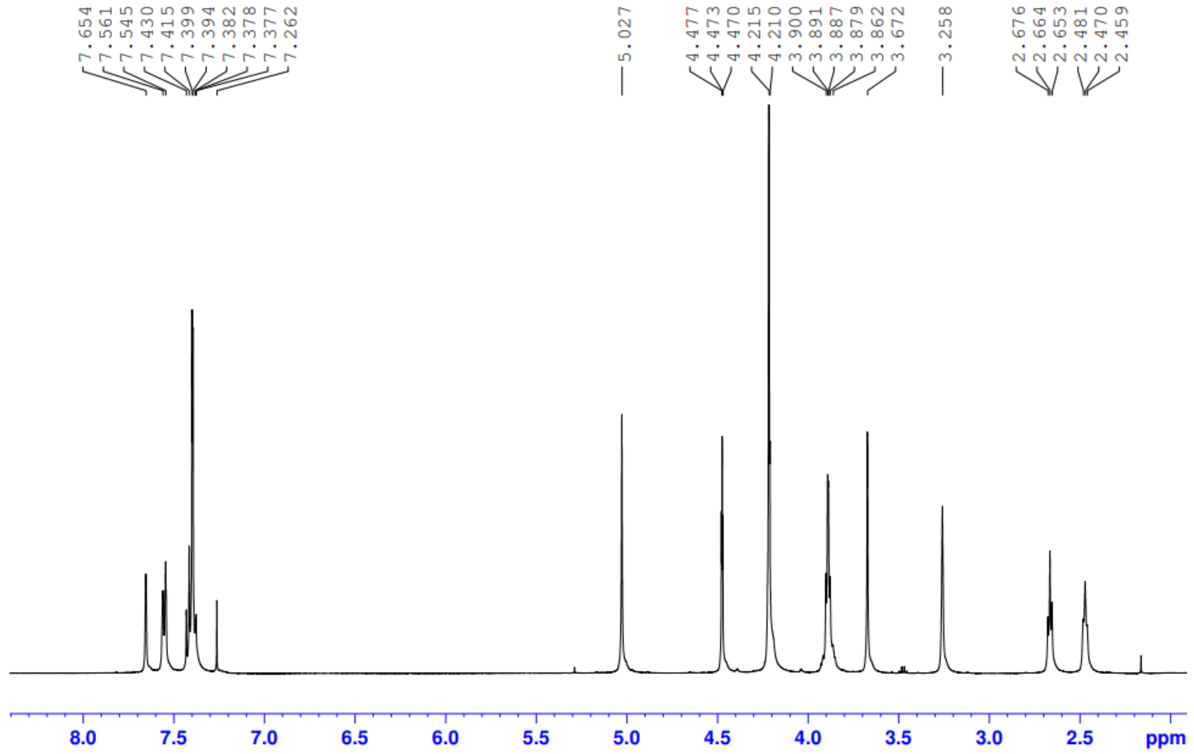

^{13}C-NMR of 16c

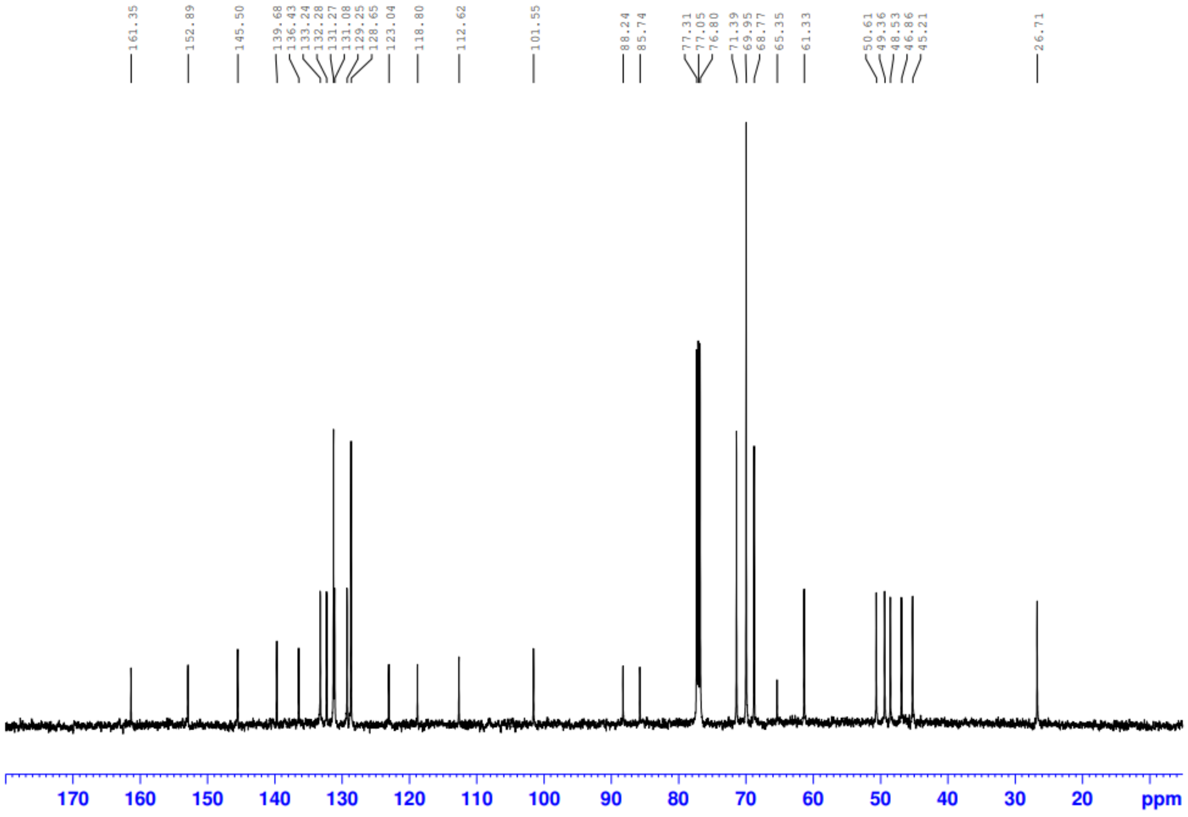

^{1}H-NMR of 16d

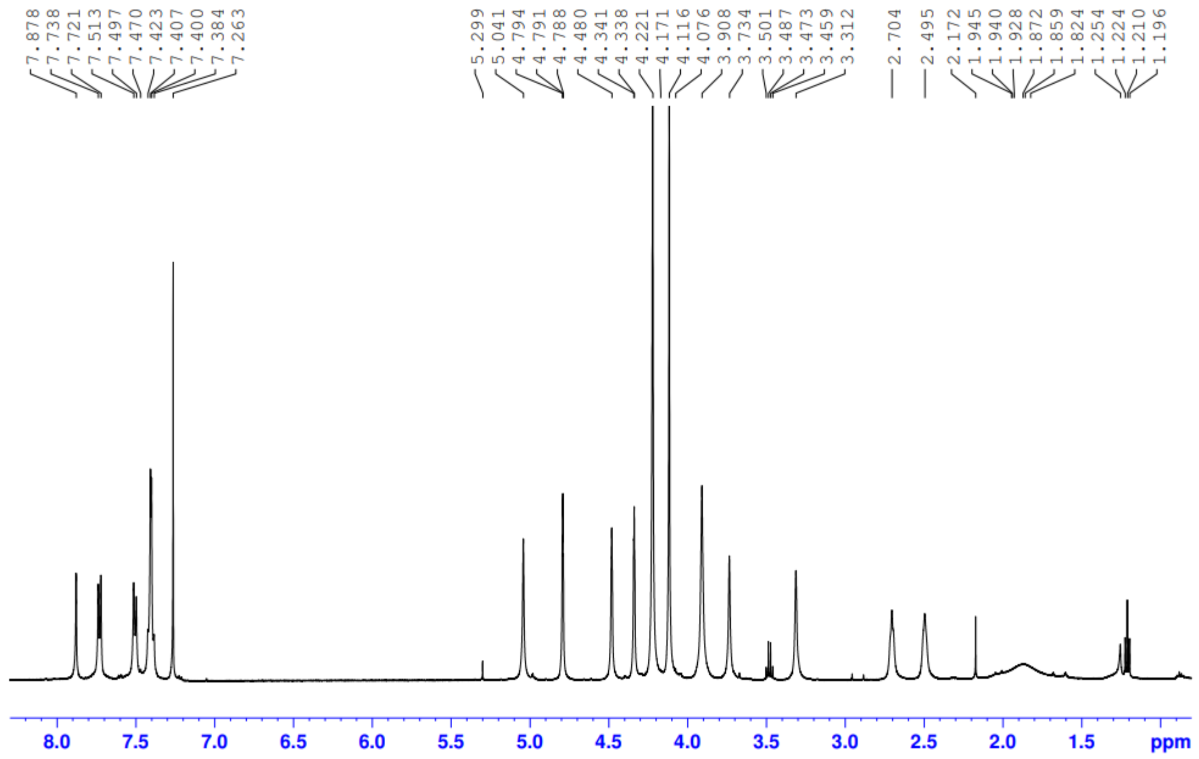

^{13}C-NMR of 16d

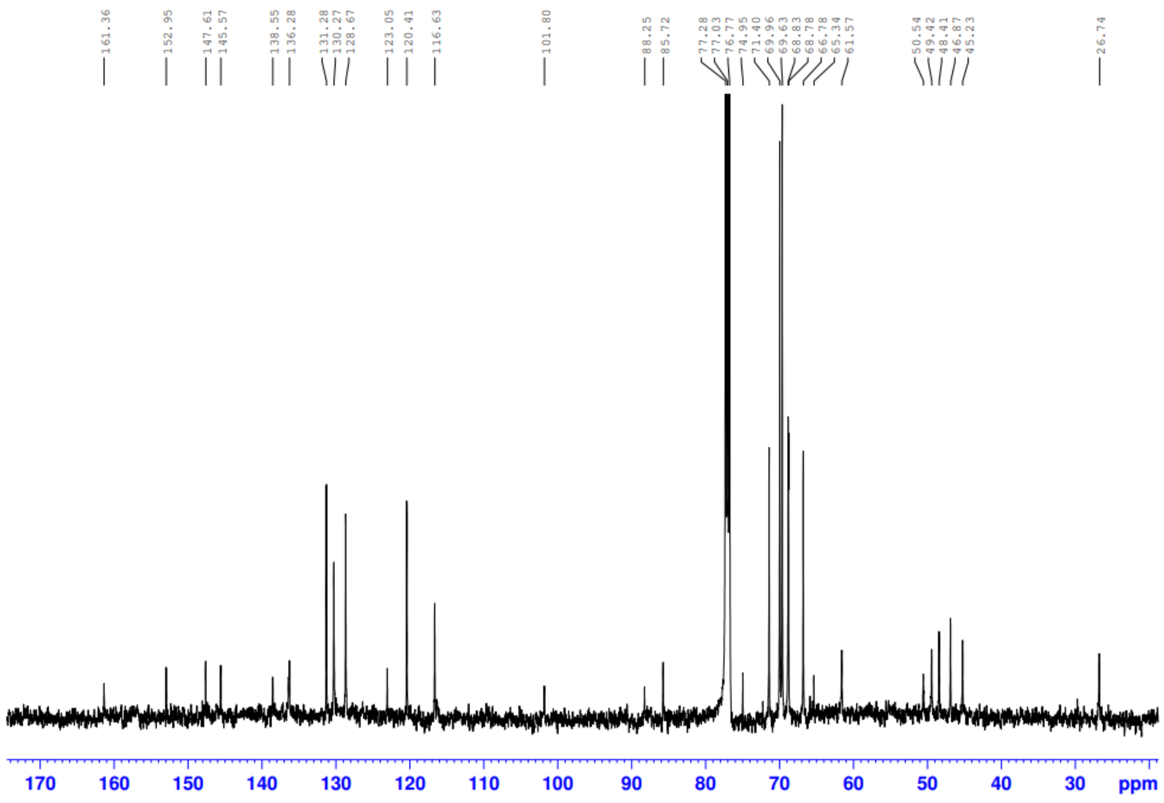

^{1}H-NMR of 17a

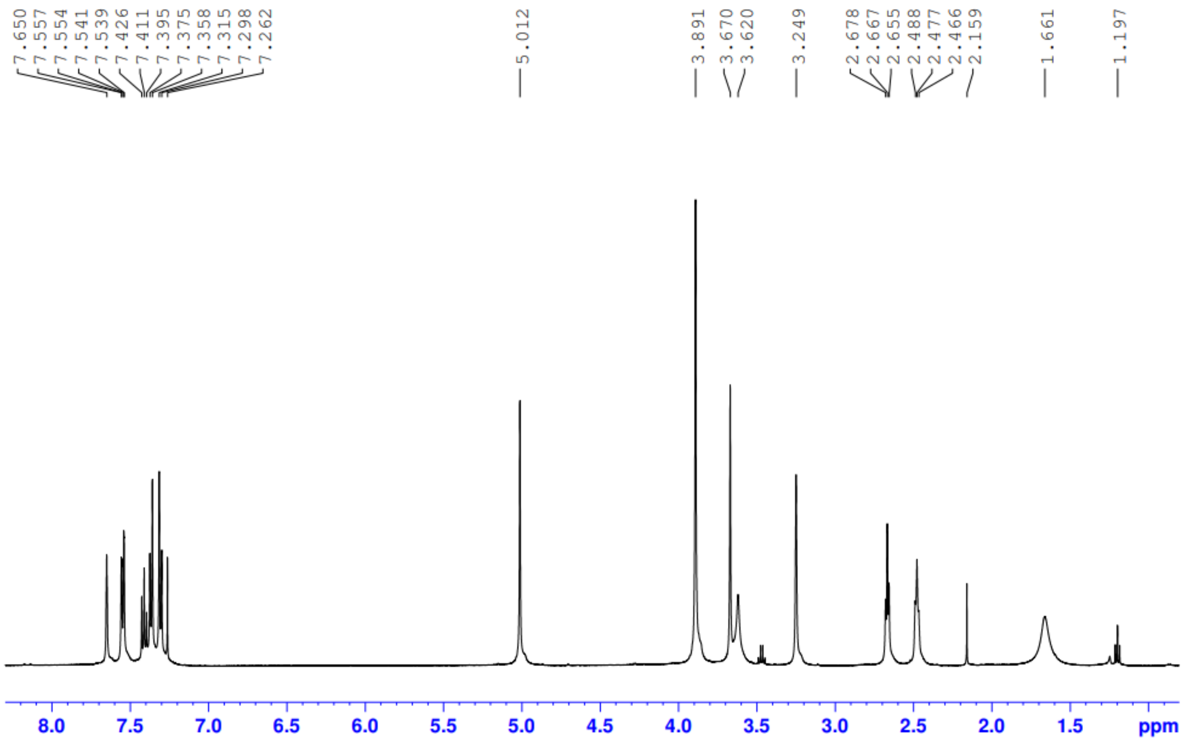

^{13}C-NMR of 17a

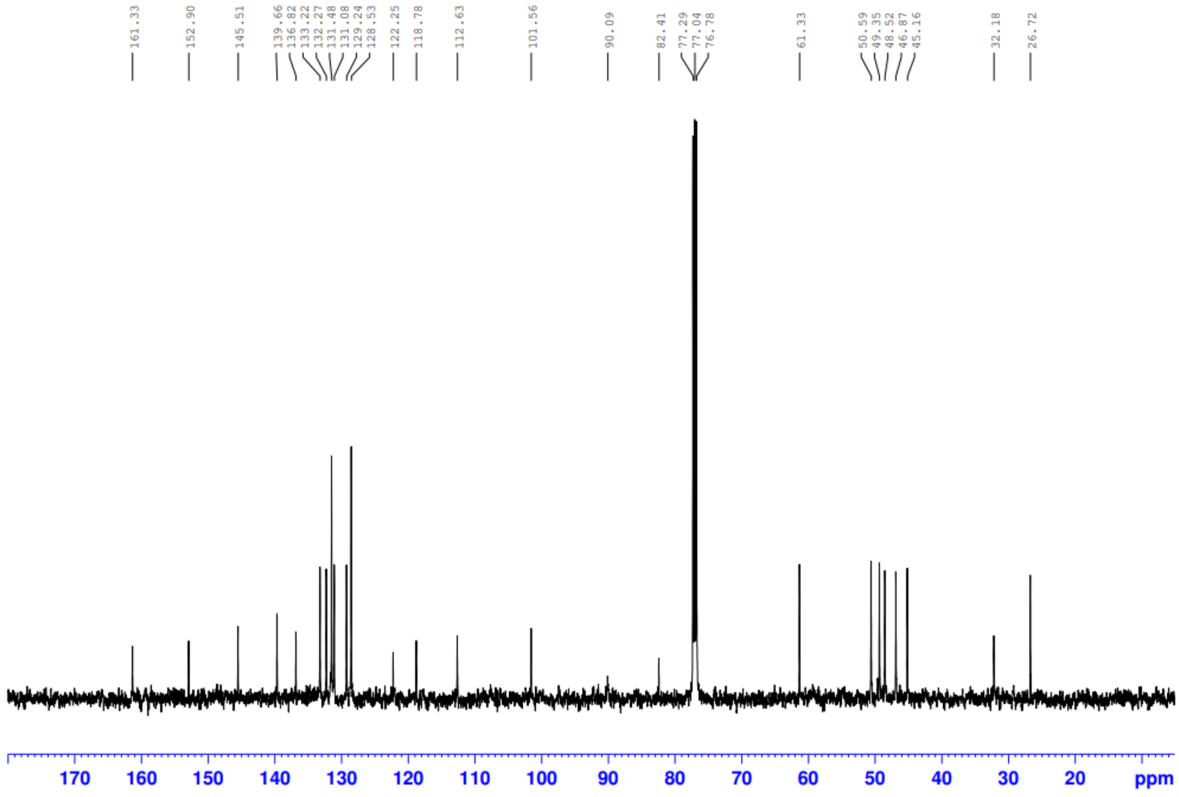

^{1}H-NMR of 17b

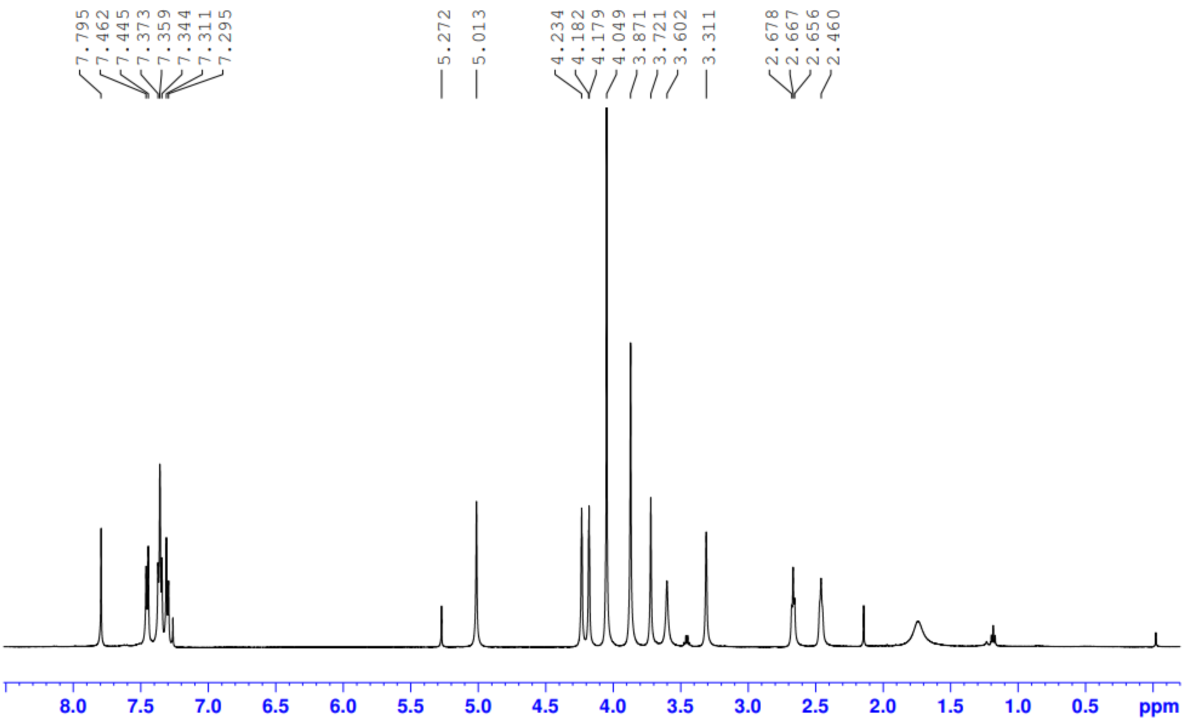

^{13}C-NMR of 17b

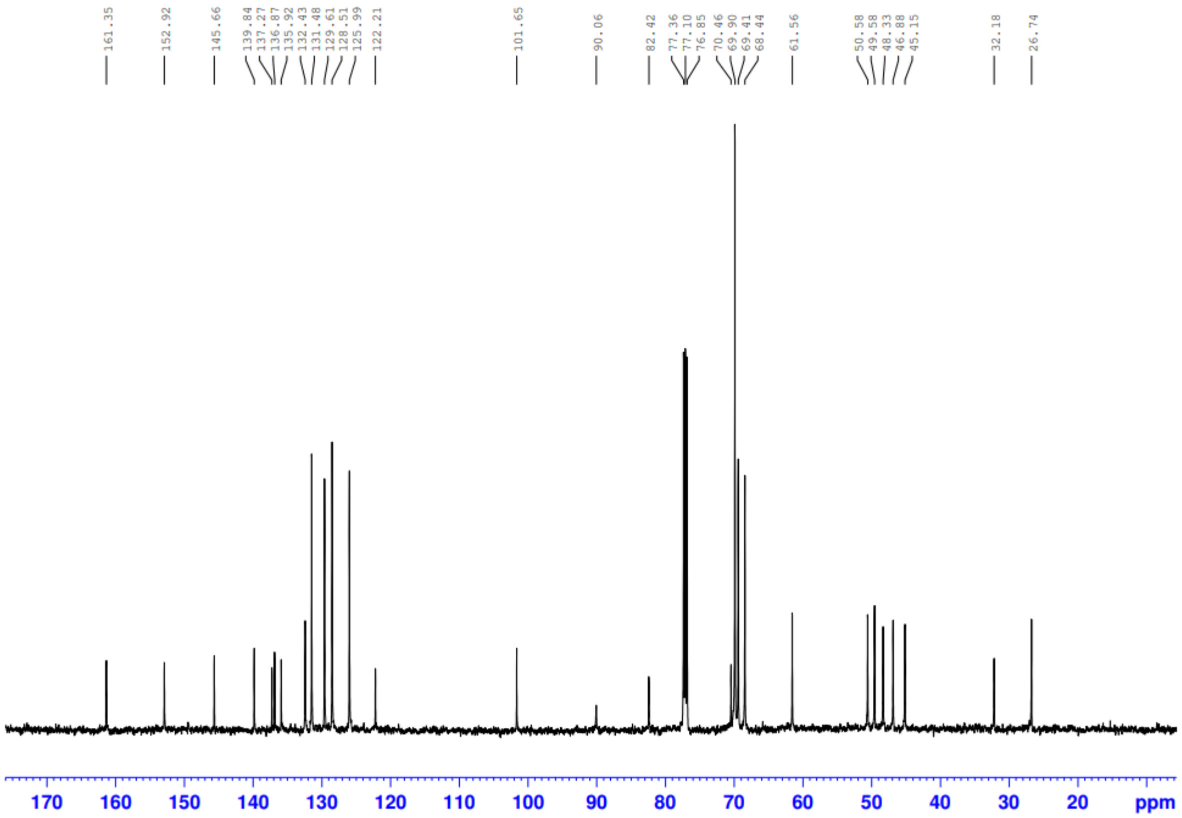

^{1}H-NMR of 17c

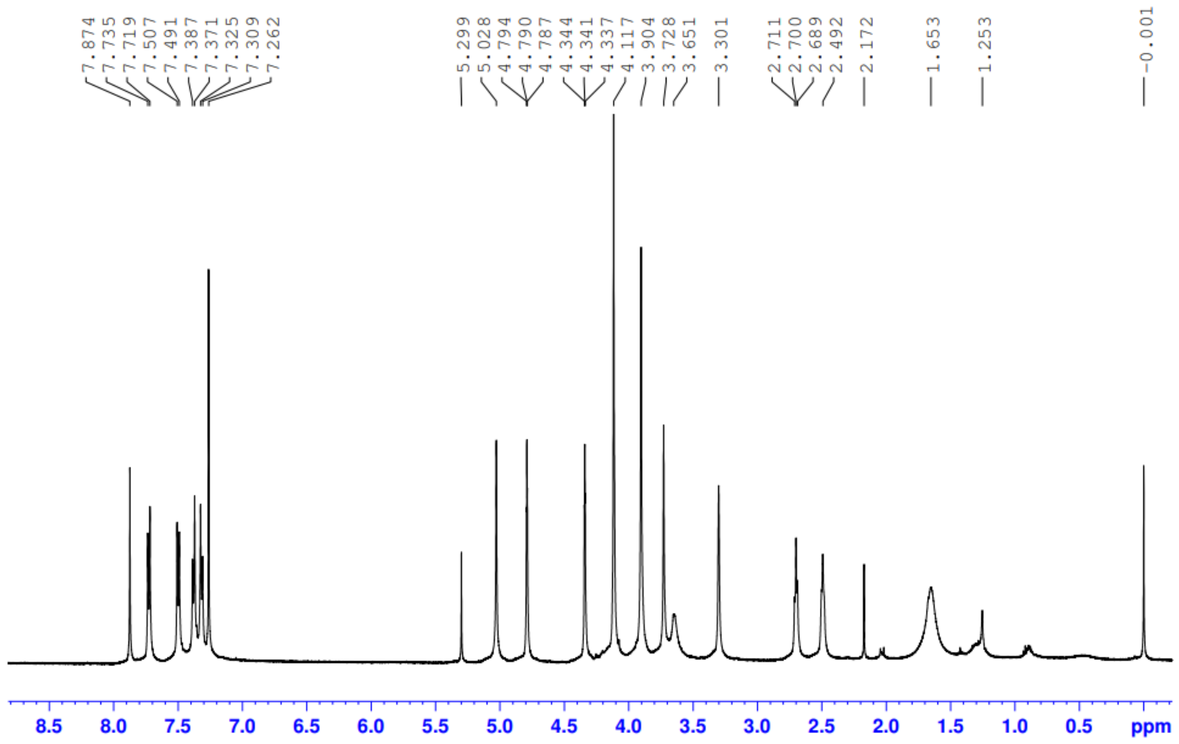

^{13}C-NMR of 17c

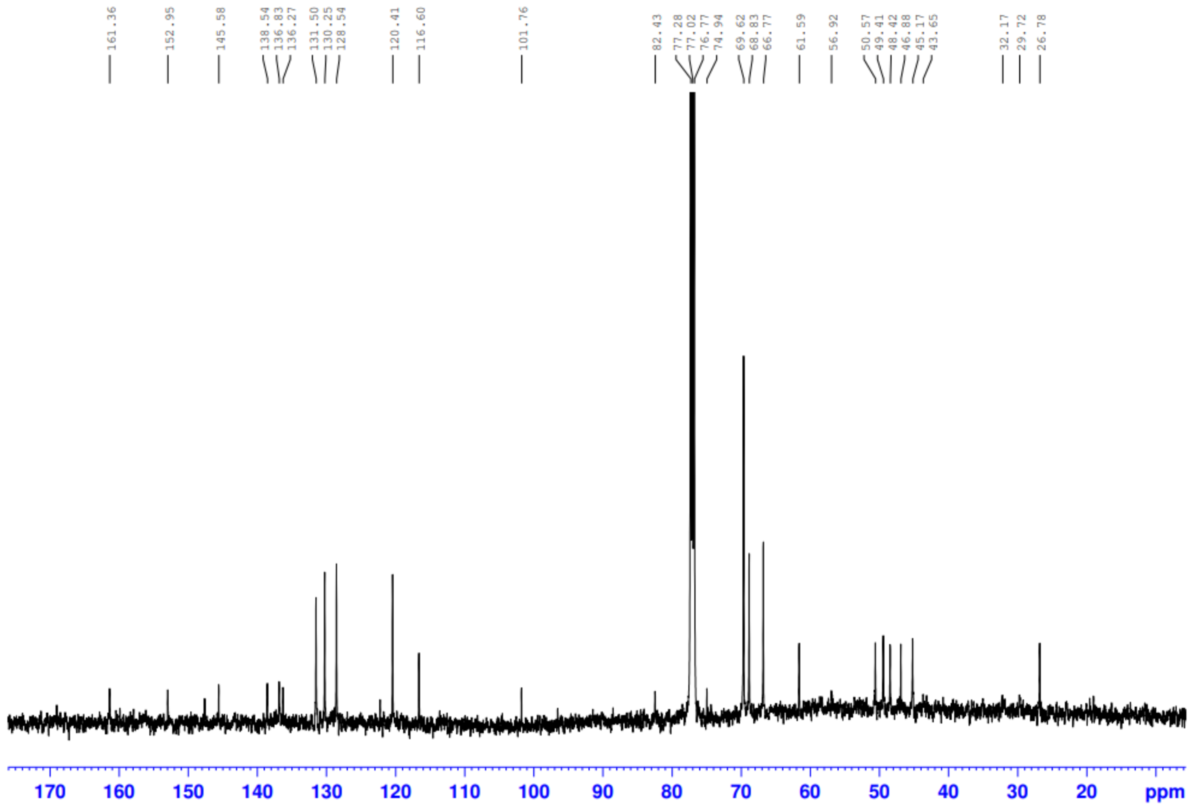

^{1}H-NMR of 17d

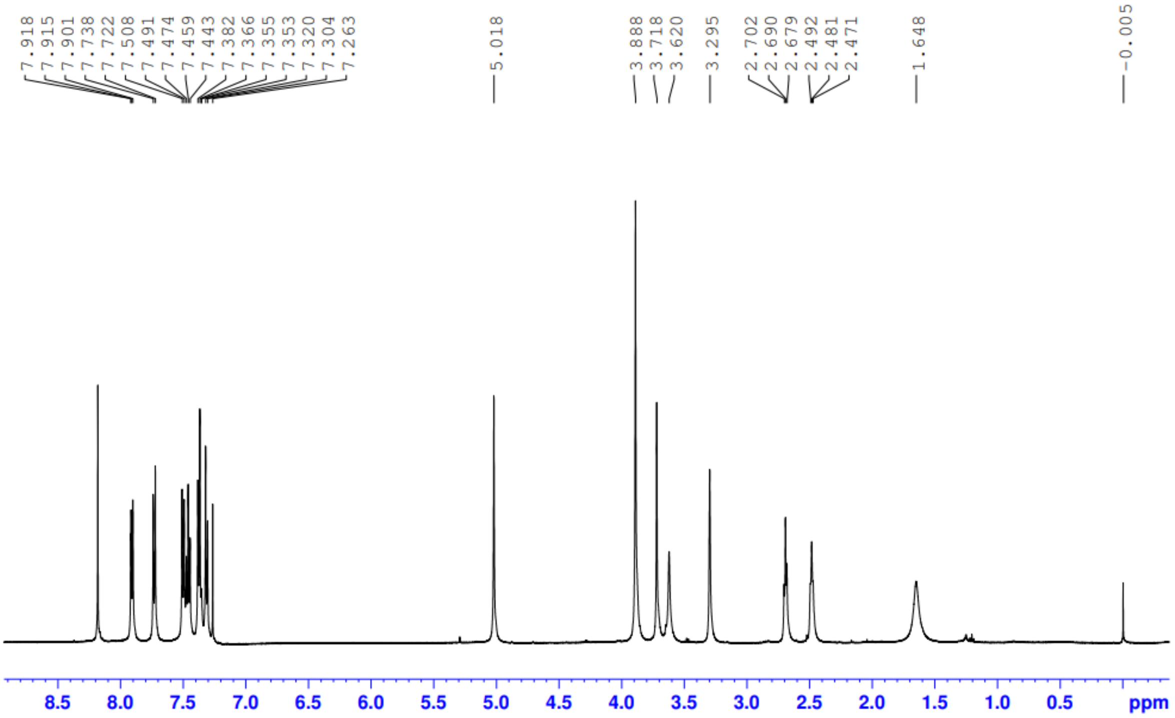

^{13}C-NMR of 17d

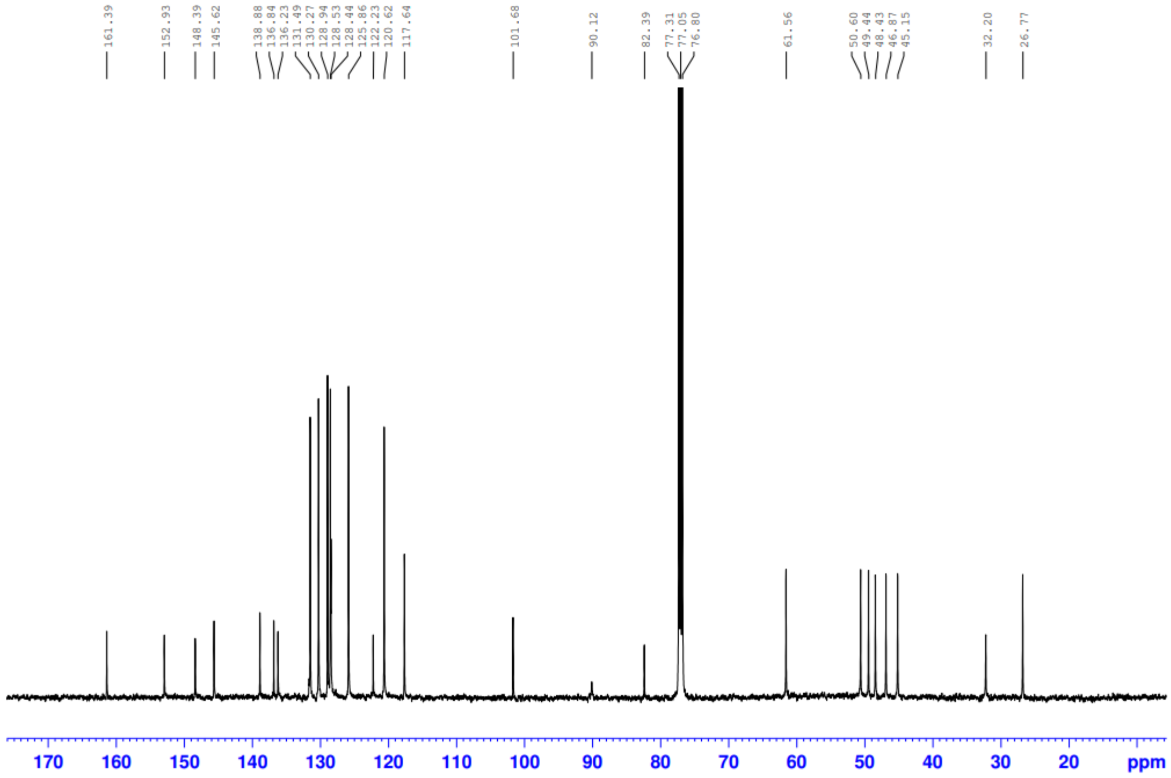

^{1}H-NMR of 18

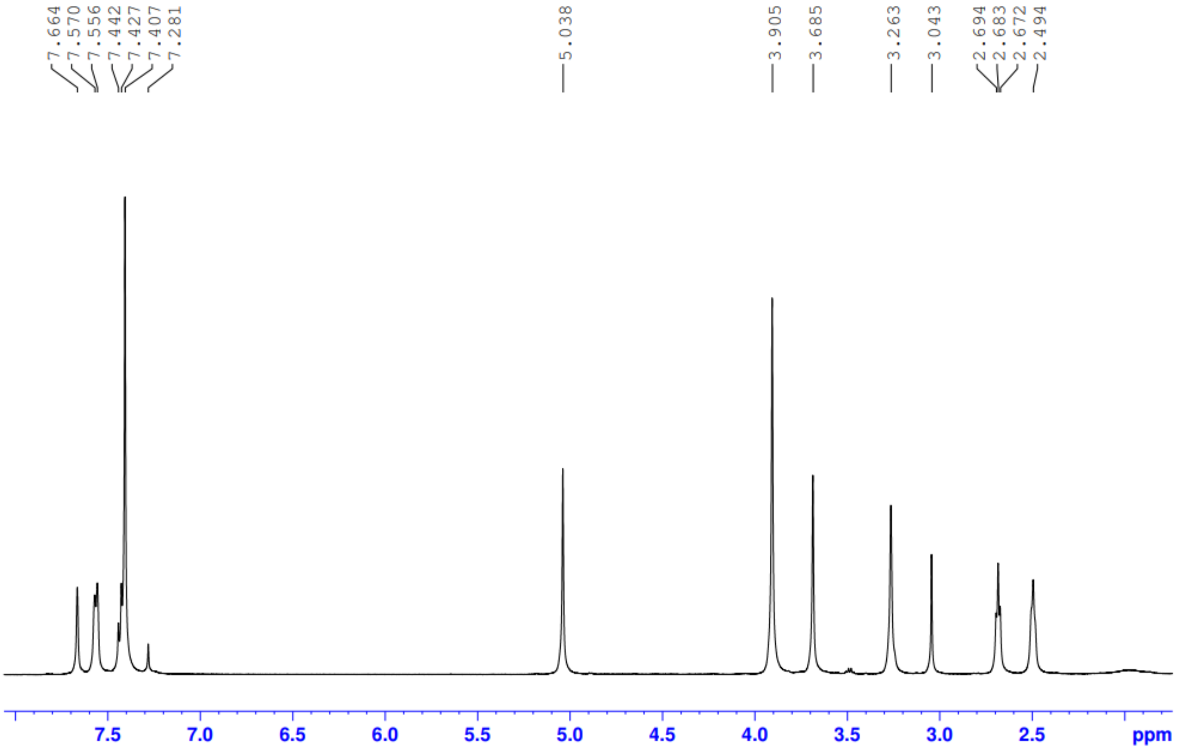

^{13}C-NMR of 18

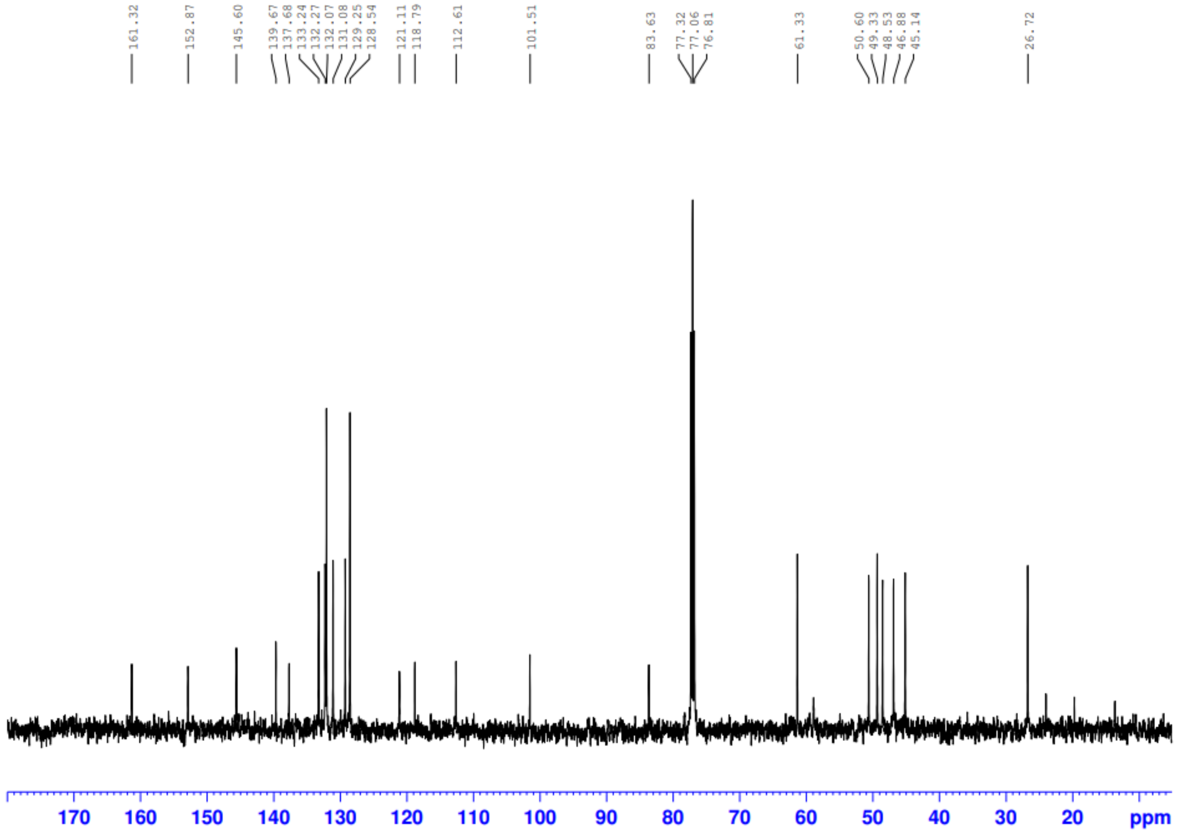

S.4. Copies of the HRMS spectra

**9a**	*m/z* calc for [$C_{35}H_{32}FFeN_7O$]+: 641.1996<br>[M – e⁻]+; found: 641.1984. mass error: 1.87 ppm

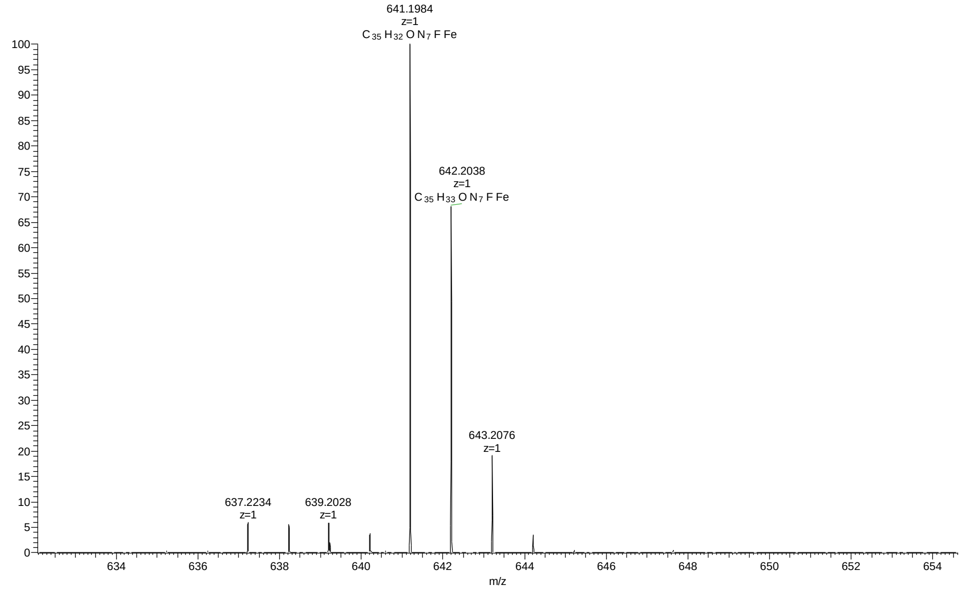

**9b**	*m/z* calc for [$C_{35}H_{31}F_2FeN_7OH$]$^+$: 660.1980<br>[M + H]$^+$; found: 660.1953. mass error: 4.095 ppm

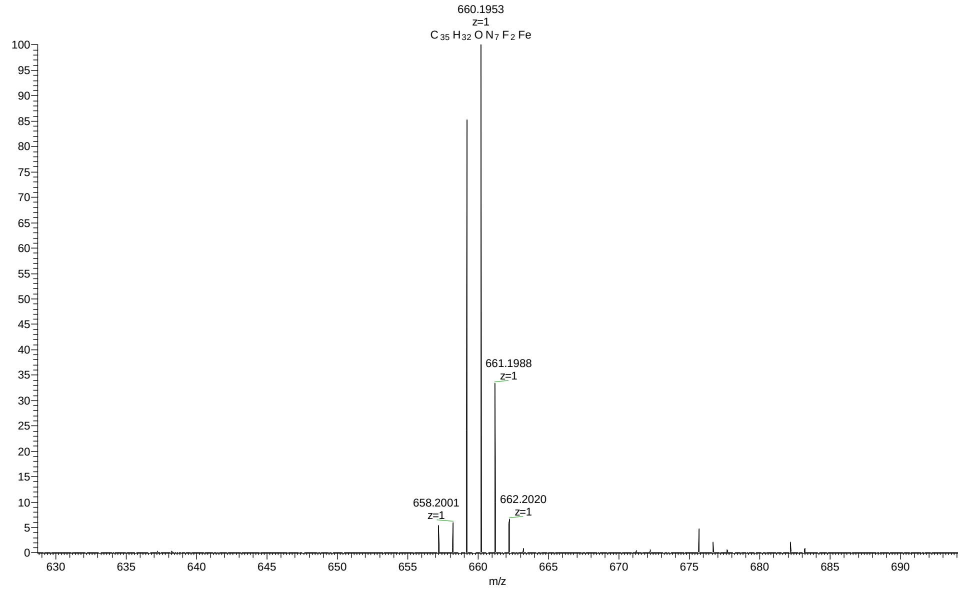

**10a**	*m*/*z* calc for $[C_{35}H_{33}FFeN_7O]^+$: 642.2075<br>[M +H] $^+$; found: 642.2058. mass error: 2.57 ppm

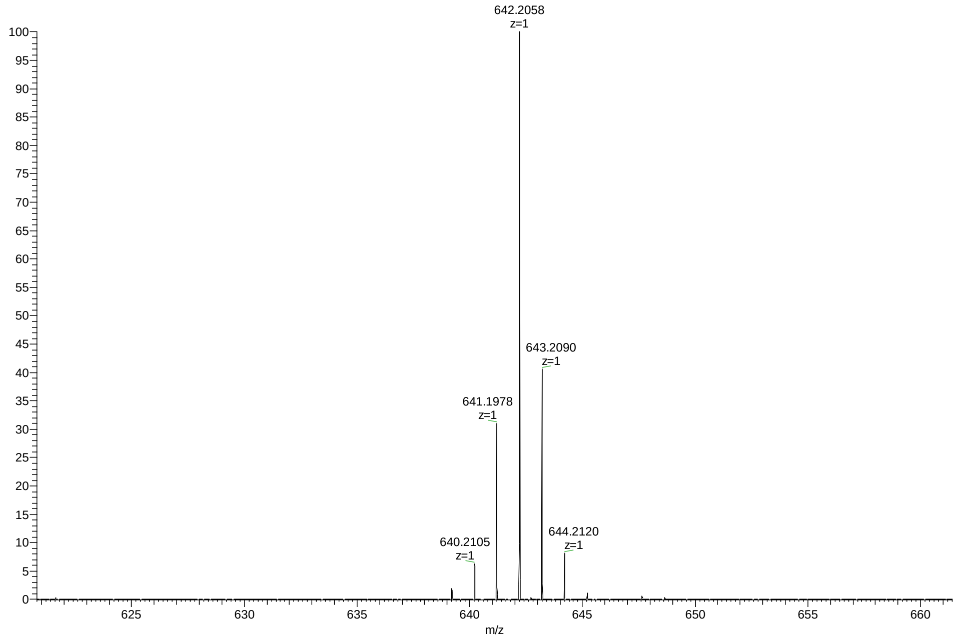

**10b**	*m*/*z* calc for $[C_{35}H_{32}F_2FeN7O]^{2+}$: 330.6027<br>$[M +2H]^{2+}$; found: 330.6019. mass error: 2.28 ppm

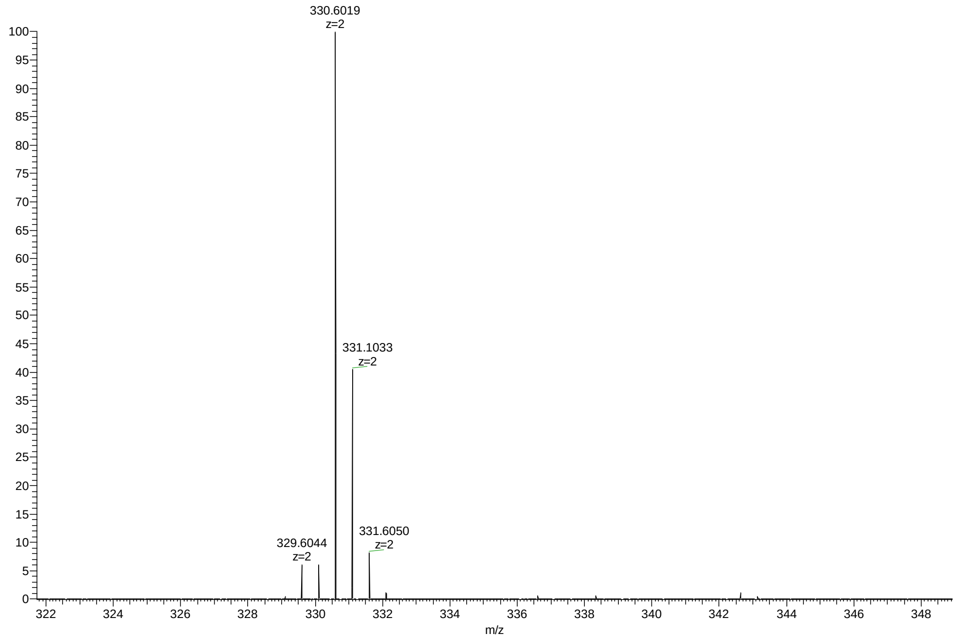

**11**	*m/z* calc for $[C_{35}H_{33}FeIN_7O]^+$: 750.1135<br>$[M+H]^+$; found: 750.1118. mass error: 2.29 ppm

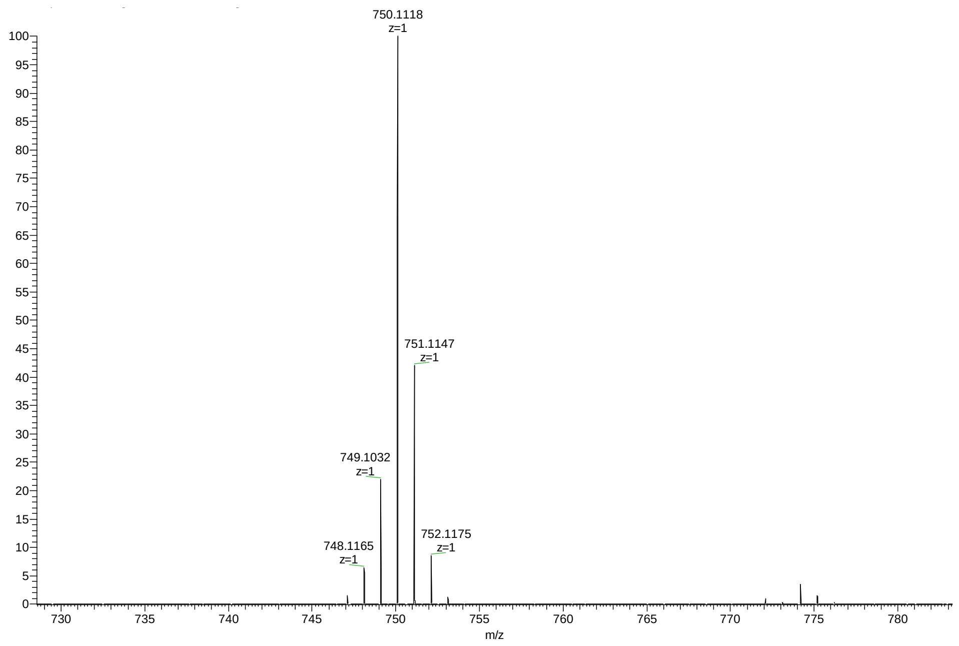

**12a**	*m/z* calc for $[C_{35}H_{33}FeIN_7O]^+$: 750.1135<br>$[M+H]^+$; found: 750.1112. mass error: 3.09 ppm

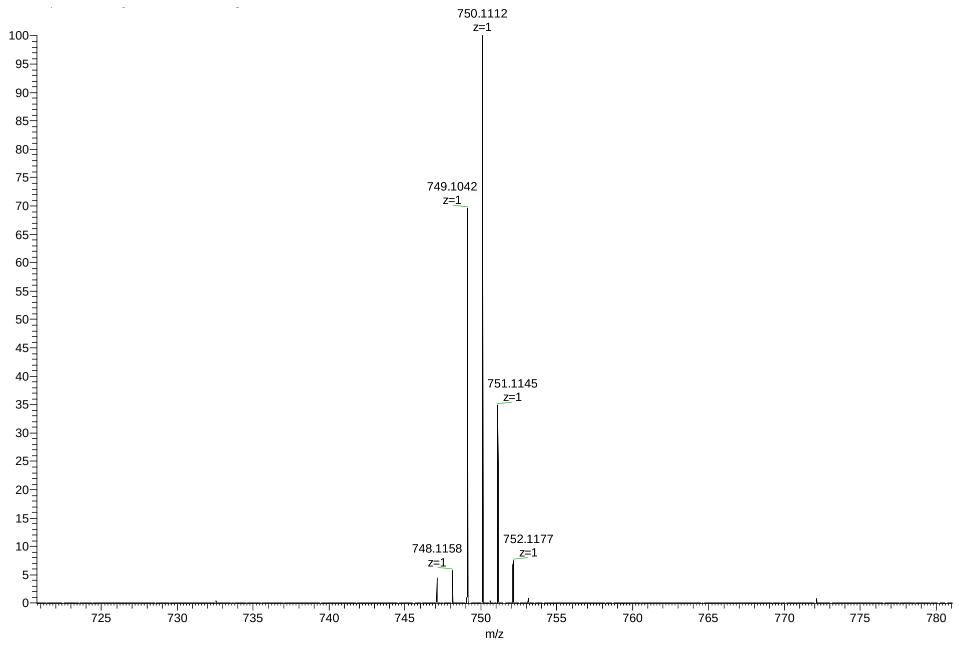

**12b**	*m/z* calc for $[C_{36}H_{36}FeN_7O]^+$: 638.2325<br>$[M+H]^+$; found: 638.2305; mass error: 3.17.

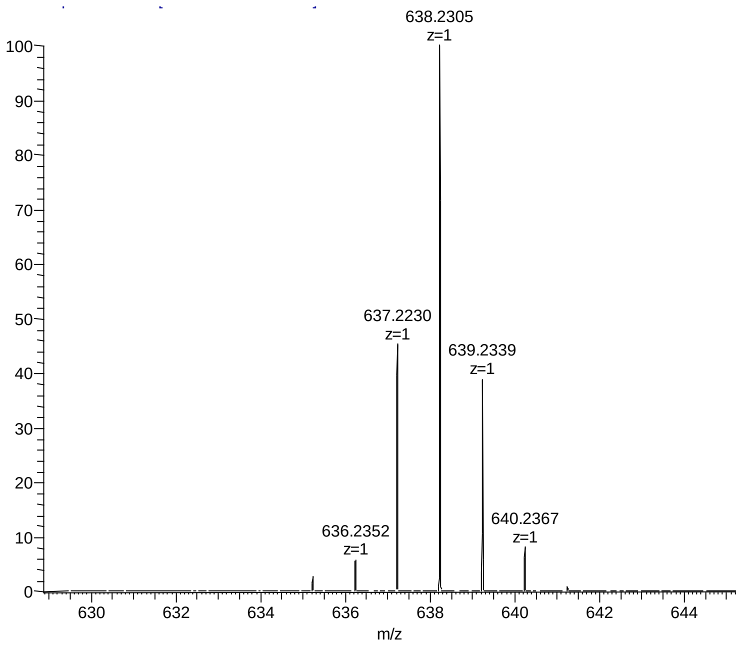

**13**	*m/z* calc for $[C_{31}H_{29}IN_7O]^+$: 642.1473<br>$[M+H]^+$; found: 642.1455. mass error: 2.78 ppm.

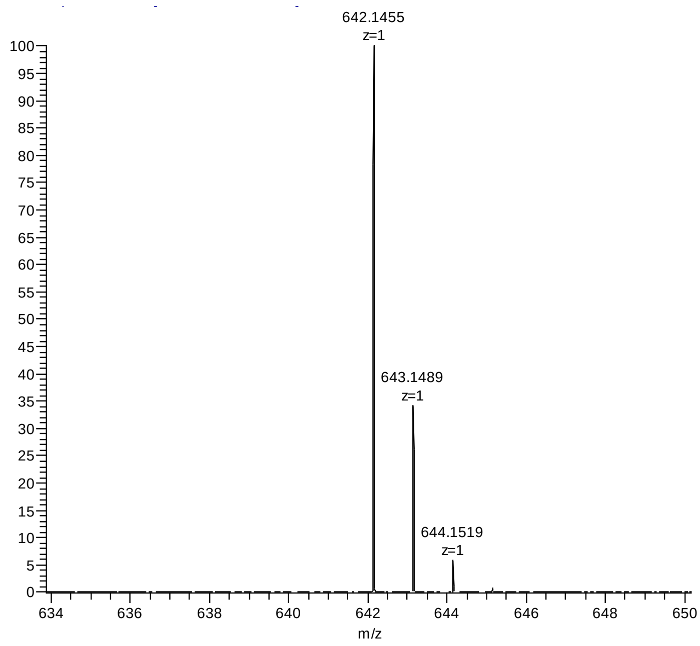

**16a**	*m/z* calc for $[C_{35}H_{32}FFeN_4O]^+$: 599.1904<br>$[M + H]^+$; found: 599.1888. mass error: 2.67 ppm

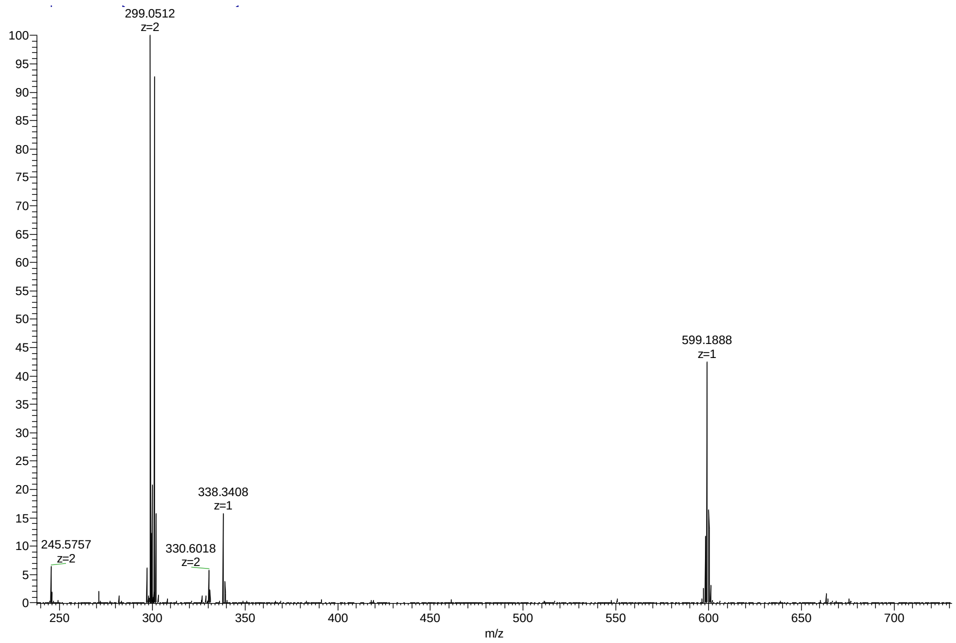

**16c**	*m/z* calc for $[C_{36}H_{32}FeN_5O]^+$: 606.1951<br>$[M + H]^+$; found: 606.1930. mass error: 3.42 ppm

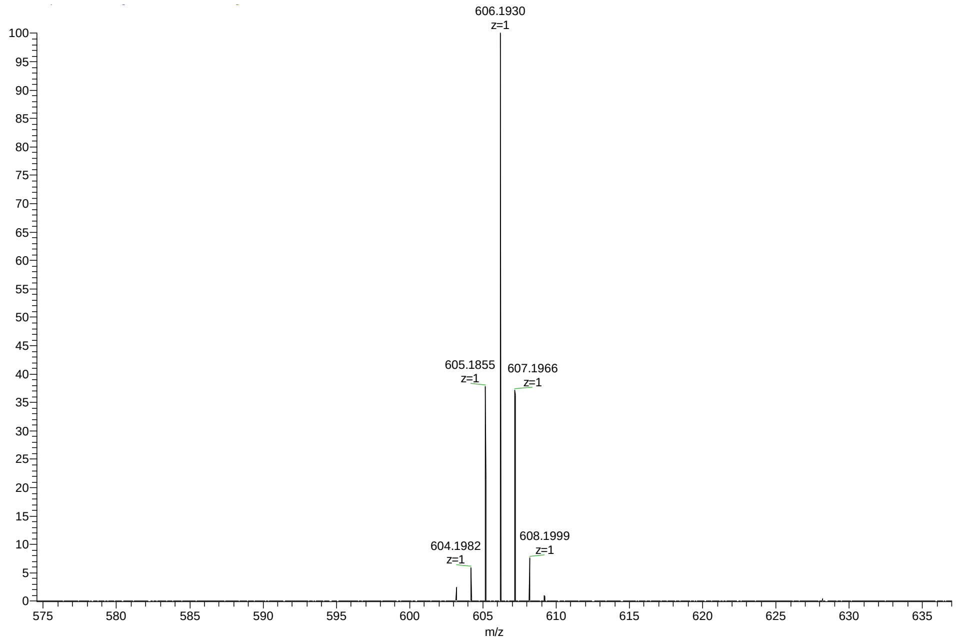

**16d**	*m/z* calc for $[C_{47}H_{42}Fe_2N_7O]^+$: 832.2144<br>$[M+H]^+$; found: 832.2120; mass error: 2.90 ppm.

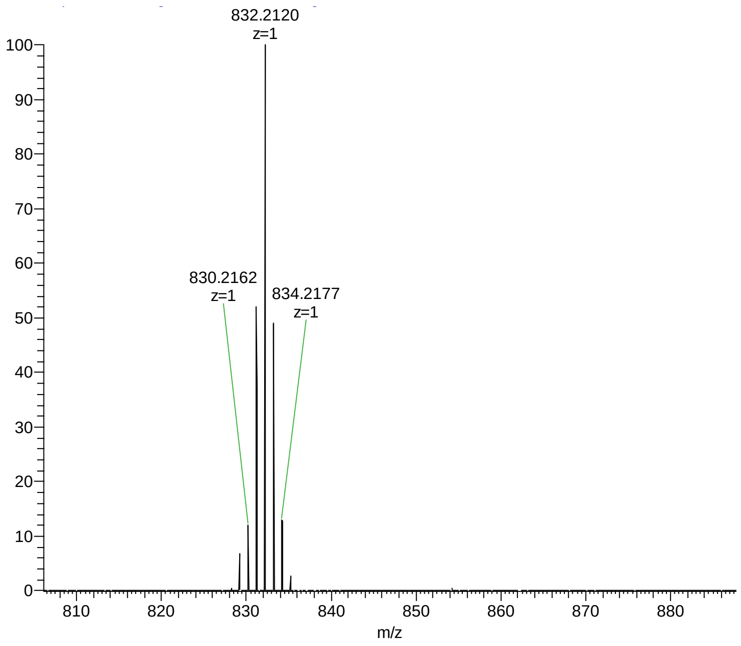

**17a**	*m/z* calc for $[C_{27}H_{27}N_6O]^+$: 451.2241<br>$[M+H]^+$; found: 451.2234. mass error: 1.52 ppm.

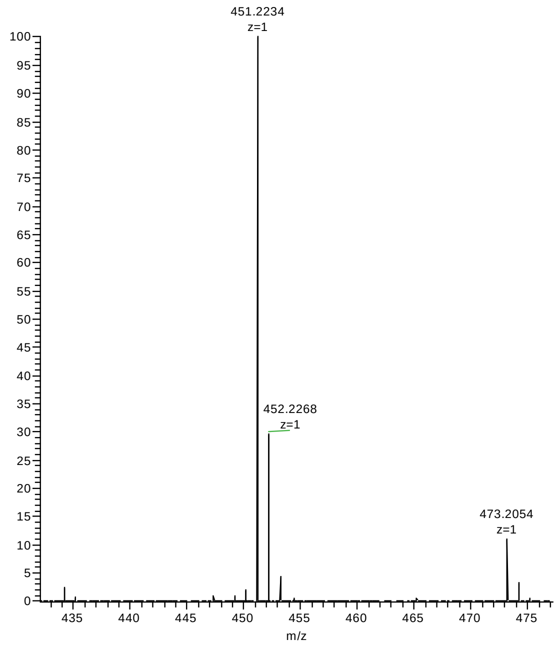

**17b**	*m/z* calc for $[C_{38}H_{37}FeN_8O]^+$: 677.2434<br>[M+H]$^+$ found: 677.2402. mass error: 4.76 ppm.

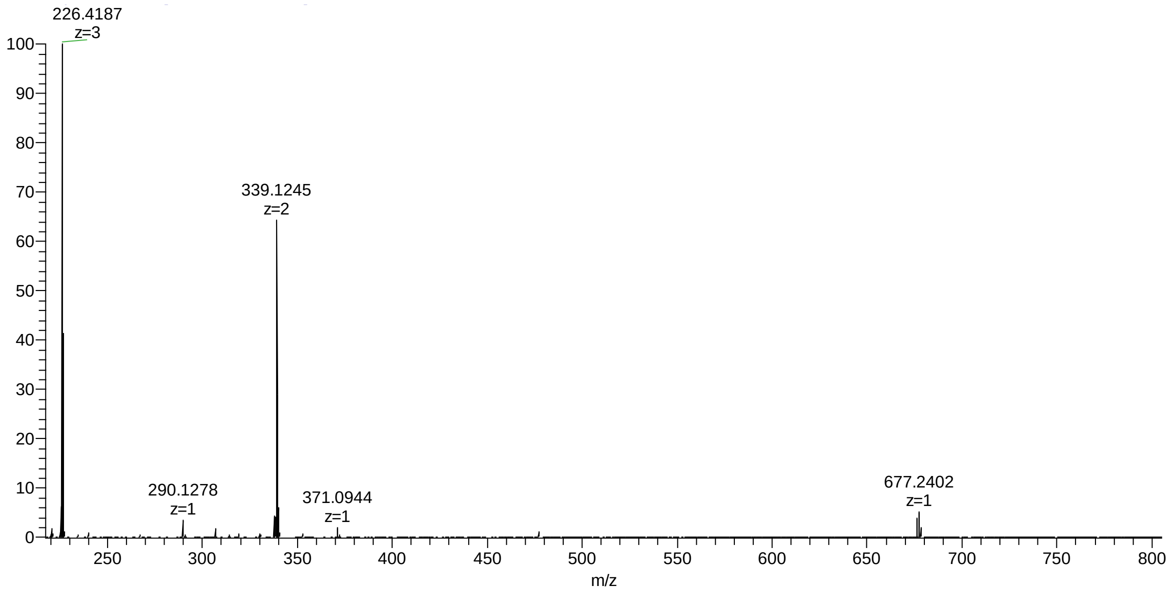

**17c**	*m/z* calc for $[C_{38}H_{37}FeN_8O]^+$: 677.2434<br>[M+H]$^+$; found: 677.2404. mass error: 4.46 ppm.

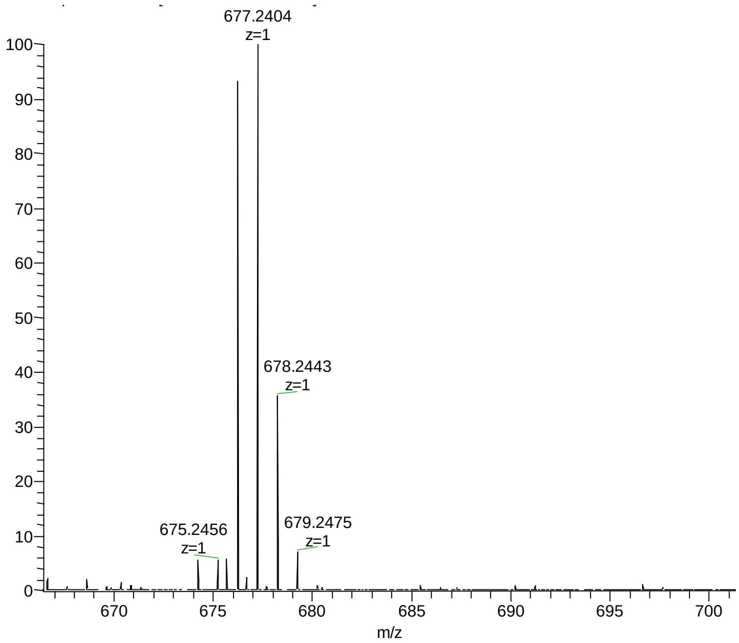

**17d**	m/z calc for $[C_{34}H_{33}N_8O]^+$: 569.2772<br>$[M+H]^+$; found: 569.2765; mass error: 1.20 ppm.

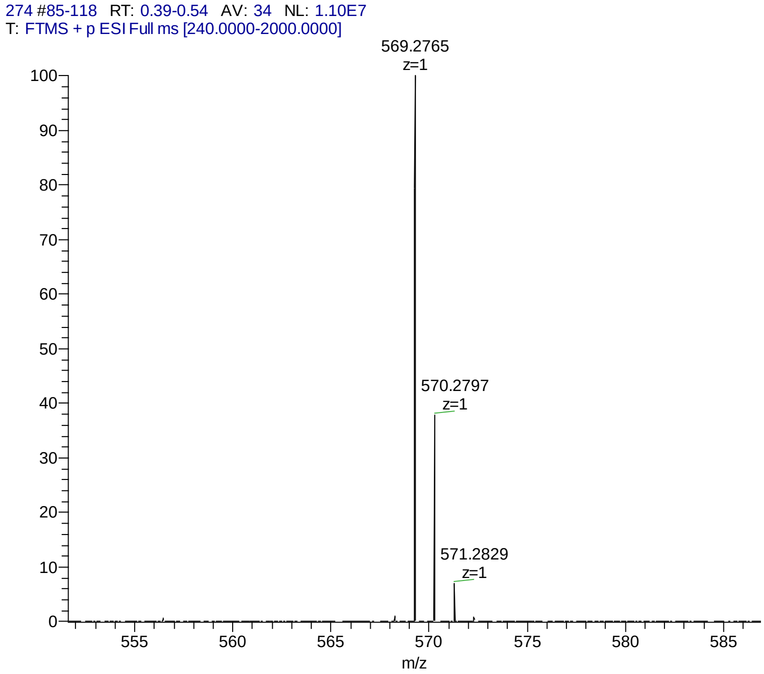

**17e**	m/z calc for $[C_{28}H_{29}N_8O]^+$: 493.2459<br>$[M+H]^+$; found: 493.2451; mass error: 1.59 ppm.

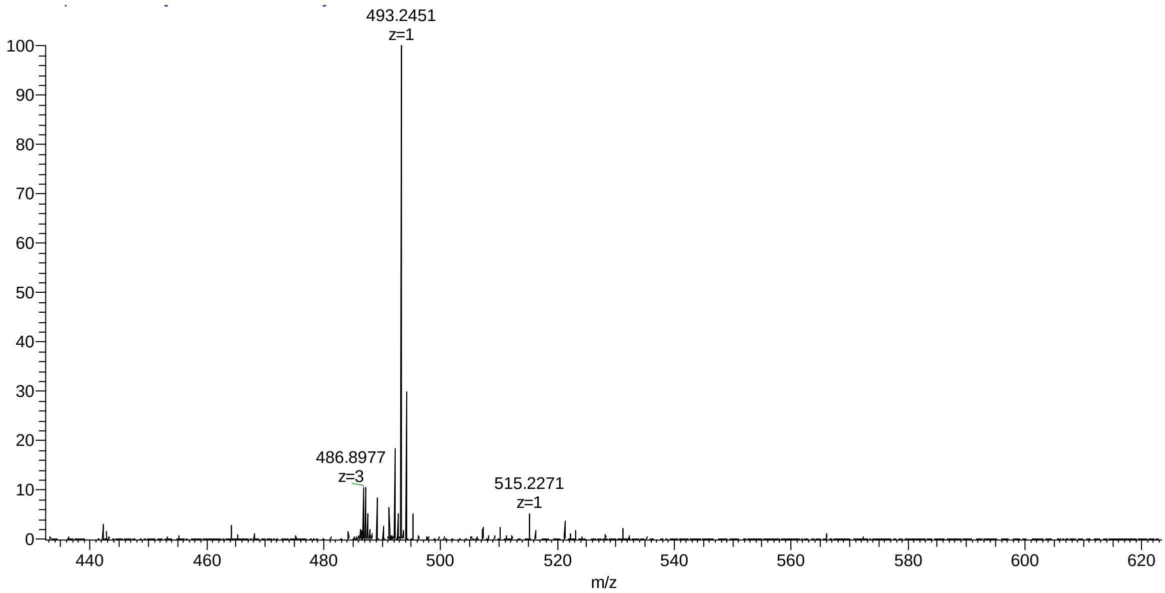

**7bf**	*m/z* calc for $[C_{23}H_{23}FIN_4O]^+$: 517.0895<br>[M+H]$^+$; found: 517.0885; mass error: 1.96 ppm.

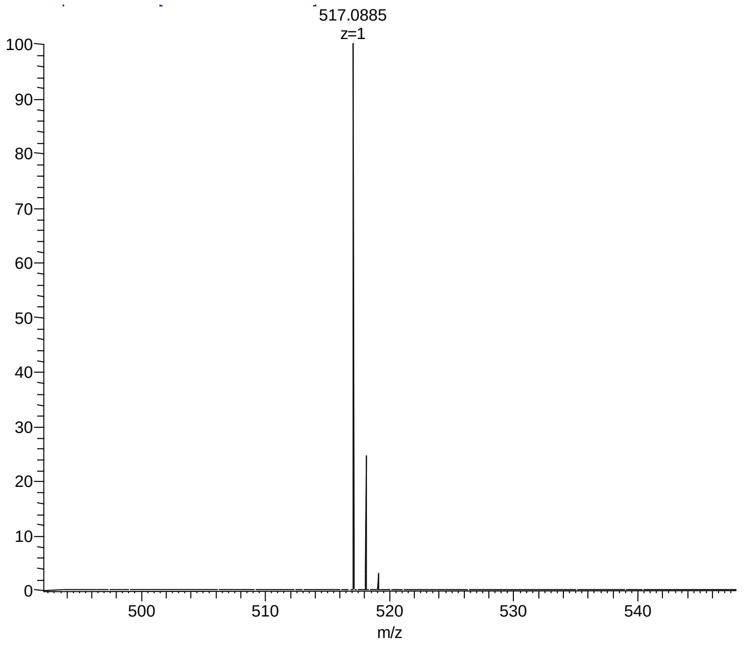

**7bh**	*m/z* calc for $[C_{23}H_{23}FN_7O]^+$: 432.1943<br>[M+H]$^+$; found: 432.1936; mass error: 1.53 ppm.

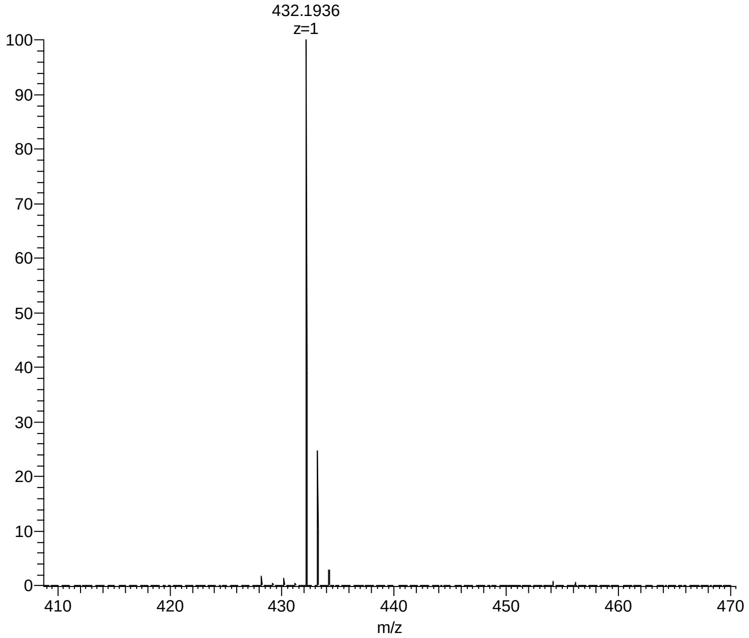

**7ch**	*m/z* calc. for $[C_{23}H_{22}F_2N_7O]^+$: 450.1848 $[M+H]^+$<br>found: 450.1843; mass error: 1.20 ppm.

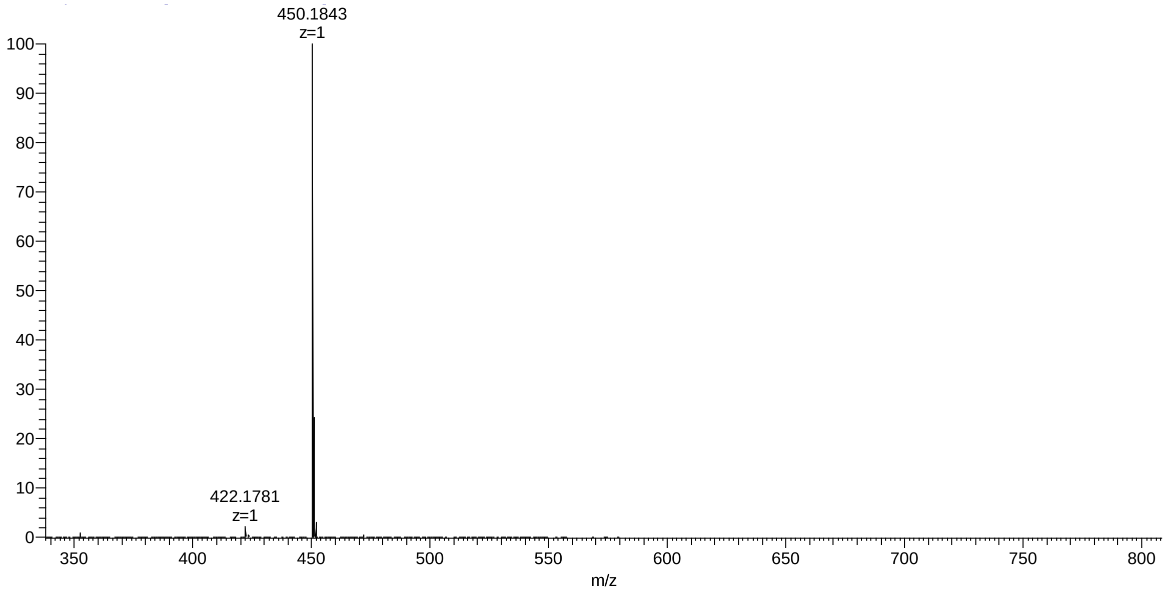

**7cf**	*m/z* calc for $[C_{23}H_{22}F_2IN_4O]^+$: 535.0801<br>$[M+H]^+$; found: 535.0790; mass error: 2.04 ppm.

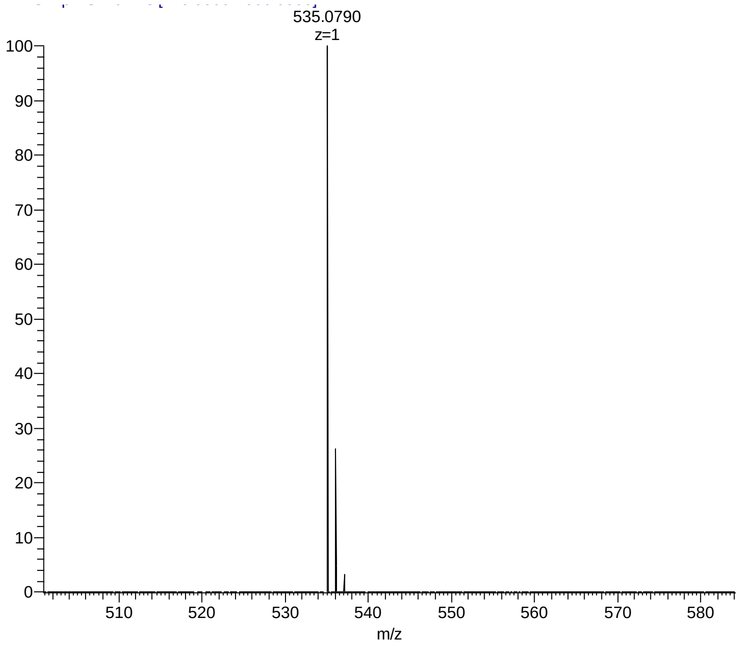

**7df**	*m/z* calc for $[C_{24}H_{23}IN_5O]^+$: 524.0942<br>$[M+H]^+$; found: 524.0934; mass error: 1.50 ppm.

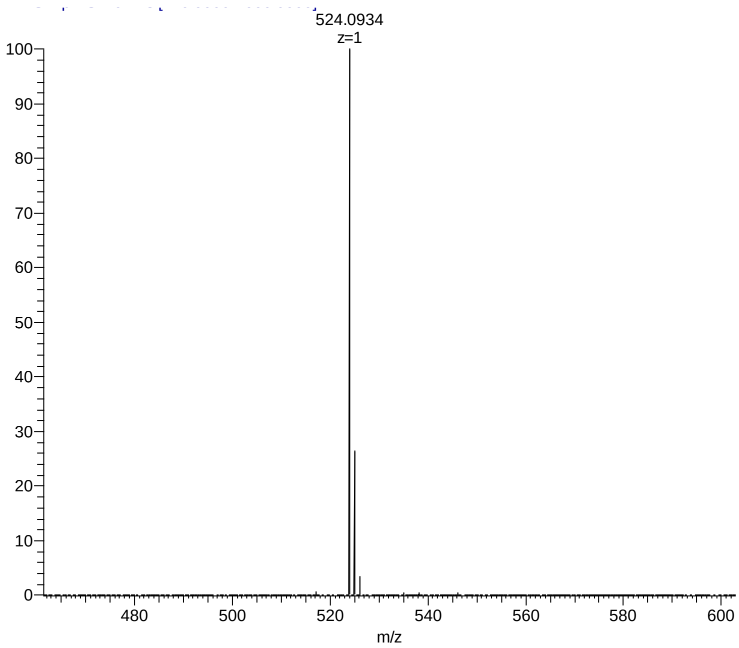

**7hf**	*m/z* calc for $[C_{23}H_{23}IN_7O]^+$: 540.1003<br>$[M+H]^+$; found: 540.0994; mass error: 1.73 ppm.

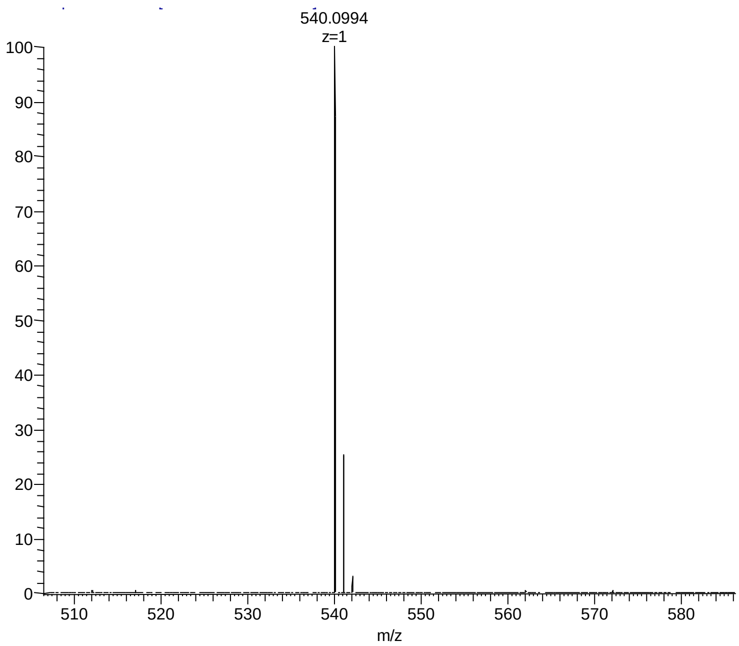

**7hg**	*m*/*z* calc for $[C_{24}H_{26}N_7O]^+$: 428.2193<br>$[M+H]^+$; found: 428.2185; mass error: 1.95 ppm.

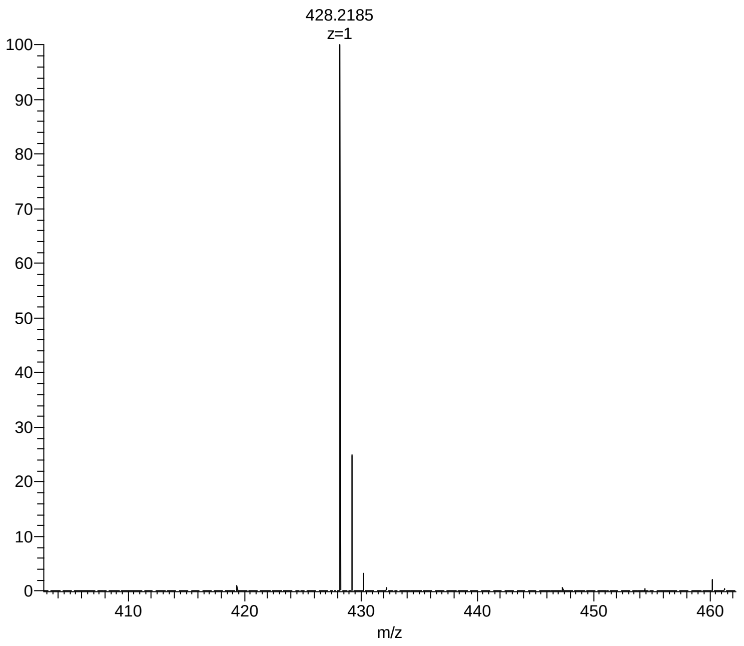

S.5. MTT Cell Viability Assay Data

Panc-1

	Cell viability (% of control)								
**Conc. / µM**	50,0	30,0	18,0	10,8	6,5	3,9	2,3	1,4	0,8
**10a**	3,9	43,2	49,5	85,5	87,0	114,2	109,6	91,5	97,6
	4,6	42,4	58,6	79,9	88,8	99,6	106,2	114,8	107,5
	1,2	12,6	21,7	35,1	57,4	120,3	124,5	108,7	108,7
	1,5	16,8	34,4	46,0	75,2	127,5	132,0	130,0	133,1
**17b**	0,8	0,9	96,5	137,9	97,7	119,6	112,1	104,7	108,5
	0,6	0,7	60,6	114,9	109,7	101,4	105,3	112,3	111,0
	0,6	0,6	15,2	147,5	153,8	151,3	129,6	116,6	111,2
	0,2	0,4	30,7	149,1	146,2	139,3	143,2	120,8	113,9
**17c**	0,9	0,8	1,0	16,2	88,9	112,6	114,3	108,5	124,9
	0,8	0,7	1,0	13,4	87,0	106,1	107,5	125,1	122,1
	0,6	0,6	0,4	2,4	96,4	130,8	145,4	117,9	118,7
	0,6	0,6	0,4	4,5	48,4	146,2	146,9	149,3	148,4
**17d**	0,6	1,0	1,1	96,8	118,5	113,1	113,5	110,7	120,7
	0,7	0,8	1,0	79,6	105,1	103,9	108,6	120,4	113,3
	0,6	0,4	0,3	1,2	153,5	147,7	128,3	120,3	128,4
	0,2	0,4	0,4	0,7	104,1	159,6	125,5	139,6	123,4
**ONC201**	42,2	66,4	65,9	64,9	51,3	64,1	68,2	102,1	95,4
	46,6	71,0	72,1	63,6	58,9	62,8	71,7	81,3	94,4
	34,1	26,3	29,1	22,6	24,7	32,9	49,2	56,2	93,0
	26,1	26,3	33,1	26,6	30,9	42,8	40,0	67,0	116,7
	26,6	28,0	31,0	27,5	29,8	37,2	46,9	59,3	87,5
	27,9	29,5	23,5	29,5	26,4	31,4	46,5	58,1	89,9

A2058

	Cell viability (% of control)								
**Conc. / µM**	50,0	30,0	18,0	10,8	6,5	3,9	2,3	1,4	0,8
**10a**	30,0	42,5	55,0	57,5	76,7	115,8	120,8	103,3	99,2
	25,6	37,0	49,3	53,7	76,7	116,3	116,3	110,1	96,0
	33,8	35,4	48,8	49,1	91,6	119,7	121,7	100,9	95,3
	23,6	37,2	60,9	64,1	83,8	144,3	160,0	133,9	109,9
**17b**	2,5	4,2	10,0	73,3	98,3	89,2	89,2	101,7	101,7
	3,5	4,4	10,6	83,7	90,7	91,6	78,4	90,7	105,7
	1,7	1,3	2,0	48,8	89,6	95,6	88,6	97,3	98,9
	1,4	1,4	2,1	48,7	99,5	90,9	102,0	106,0	100,6
**17c**	6,7	3,3	3,3	3,3	26,7	31,7	102,5	95,8	95,0
	7,0	4,4	3,5	2,6	21,1	39,6	85,5	80,2	85,5
	2,3	1,7	1,0	2,0	17,7	58,8	94,9	91,2	95,9
	3,6	2,1	1,8	2,5	17,5	68,4	87,4	93,8	104,2
**17d**	2,5	4,2	2,5	4,2	40,0	82,5	90,8	101,7	100,0
	3,5	4,4	2,6	5,3	52,0	75,8	72,2	92,5	111,9
	0,7	1,3	1,0	2,3	54,1	78,5	111,0	105,9	101,6
	1,8	1,1	1,4	3,6	51,2	80,9	95,2	100,6	99,5
**ONC201**	40,8	41,7	49,2	49,2	44,2	40,0	50,8	63,3	75,0
	42,3	47,6	58,1	45,8	45,8	50,2	51,1	66,1	74,0
	39,1	48,1	49,1	54,5	54,1	57,5	54,5	84,6	97,3
	37,2	45,5	48,7	53,3	50,5	51,6	61,2	75,2	88,4

EBC-1

	Cell viability (% of control)								
**Conc. / µM**	50,0	30,0	18,0	10,8	6,5	3,9	2,3	1,4	0,8
**10a**	1,4	2,5	18,0	27,4	48,7	84,4	92,0	96,7	95,6
	2,7	2,7	18,4	30,6	52,5	98,8	106,1	90,0	96,5
	1,6	1,9	21,0	34,0	61,8	79,8	100,8	127,2	109,2
	1,0	1,8	17,7	32,5	53,6	83,0	103,0	93,0	95,1
**17b**	1,4	1,8	22,7	71,5	93,1	107,9	97,4	98,2	97,4
	1,9	2,7	23,7	72,4	84,3	99,2	96,9	96,5	96,1
	1,4	1,4	11,4	54,5	106,5	106,2	102,7	108,9	101,3
	1,0	1,0	11,5	45,1	100,5	103,5	106,4	97,9	106,4
**17c**	1,1	2,2	1,8	6,1	37,9	80,8	76,5	98,9	93,5
	1,9	2,3	2,3	6,1	39,1	86,6	81,2	85,4	91,5
	1,4	1,4	1,1	9,3	51,2	86,3	97,8	99,7	99,9
	1,3	1,3	1,5	10,0	50,2	100,2	87,4	108,7	121,2
**17d**	2,5	1,4	14,1	75,1	101,8	96,0	96,4	94,2	91,7
	3,1	3,1	15,3	83,9	103,0	97,7	94,2	87,7	90,0
	1,1	1,6	7,1	66,4	115,7	117,6	112,2	107,3	98,3
	0,8	1,3	12,8	68,7	98,4	101,5	101,5	114,3	112,8
**ONC201**	18,8	25,6	25,3	26,7	28,9	28,5	35,4	60,3	71,5
	13,4	23,4	23,4	26,0	23,0	26,0	35,2	52,9	60,9
	15,5	23,1	29,7	30,2	40,8	37,3	50,7	70,0	87,1
	21,0	27,2	34,3	27,4	30,2	42,5	56,1	72,0	99,9

Fadu

Cell viability (% of control)									
**Conc. / uM**	50,0	30,0	18,0	10,8	6,5	3,9	2,3	1,4	0,8
**10a**	5,6	6,5	11,5	16,5	35,5	67,9	81,3	96,1	81,3
	4,0	5,0	12,9	20,3	42,1	86,0	103,3	113,0	89,2
	8,7	8,0	13,0	20,0	33,4	94,2	107,3	105,2	108,6
	6,0	8,3	10,7	22,6	34,8	81,4	107,9	91,3	97,7
**17b**	1,3	1,3	28,2	69,0	82,7	70,8	91,7	73,3	100,1
	1,0	1,0	25,2	91,3	98,5	108,6	97,1	115,8	92,5
	0,4	0,7	16,6	79,3	84,2	100,3	107,8	99,1	101,3
	1,1	1,5	23,4	84,5	93,4	90,1	91,8	105,0	103,3
**17c**	1,3	1,3	0,8	1,5	37,0	88,0	72,3	90,1	108,4
	1,2	1,0	1,4	1,2	44,3	94,5	108,2	110,8	91,7
	1,0	0,7	0,7	1,3	46,5	105,7	96,3	105,7	105,9
	1,6	1,2	1,2	2,4	62,1	92,0	98,9	103,7	98,5
**17d**	0,8	1,5	1,3	34,3	79,0	77,5	98,4	85,7	122,2
	1,2	1,2	1,2	58,0	102,5	100,5	104,4	112,4	102,1
	0,6	0,9	0,6	37,2	90,5	108,7	97,7	94,9	94,2
	1,1	1,5	1,3	44,2	97,1	97,6	103,6	100,1	105,2
**ONC201**	9,2	9,6	9,2	10,4	13,6	18,2	23,4	43,7	68,1
	9,7	12,7	9,7	13,5	14,7	16,9	25,8	60,4	81,4
	15,1	17,2	19,1	20,0	22,3	22,9	33,4	70,3	86,6
	12,9	18,5	19,7	18,9	25,0	21,2	36,3	67,7	97,1

S.6. CellTiter-Glo Cell Viability Assay Data

17c

	Cell viability (% of control)							
**Conc. / µM**	30,0	18,0	10,8	6,5	3,9	2,3	1,4	0,8
**PANC-1**	0,0	0,1	0,1	7,6	67,4	95,5	93,0	89,1
	0,0	0,0	0,0	9,1	69,3	97,9	94,2	95,0
	0,0	0,0	0,0	7,8	71,5	97,6	91,5	94,4
**A2058**	0,1	0,1	0,6	2,8	38,8	85,4	117,9	97,9
	0,0	0,0	0,6	2,1	45,9	84,8	122,9	99,4
	0,0	0,1	0,5	2,3	47,9	90,9	95,9	97,5
**EBC-1**	0,1	0,1	0,6	2,9	39,7	87,2	116,8	96,8
	0,0	0,0	0,6	2,2	46,1	85,2	120,4	99,0
	0,0	0,1	0,5	2,3	49,3	92,7	98,6	98,5
**Fadu**	0,1	0,1	0,1	65,6	100,0	103,1	105,0	103,2
	0,0	0,0	0,0	67,2	99,7	102,6	96,4	100,9
	0,0	0,0	0,1	62,5	97,5	95,9	102,7	98,6
**P.fibroblast**	0,1	0,1	100,9	125,2	104,5	105,5	108,4	97,3
	0,0	0,1	101,5	127,3	104,6	104,0	103,0	104,6
	0,0	0,1	103,3	128,1	106,3	102,7	103,8	101,4

17d

	Cell viability (% of control)							
**Conc. / µM**	30,0	18,0	10,8	6,5	3,9	2,3	1,4	0,8
**PANC-1**	0,0	0,0	0,0	4,9	94,3	97,6	101,9	99,8
	0,0	0,0	0,0	4,0	96,1	98,7	100,1	103,5
	0,1	0,1	0,1	2,7	93,9	102,7	101,5	99,1
**A2058**	0,0	0,0	0,4	18,6	93,3	103,4	103,0	98,7
	0,0	0,0	0,4	23,8	90,8	102,3	99,8	98,3
	0,1	0,1	0,5	26,9	92,3	110,0	102,7	101,3
**EBC-1**	0,0	0,0	0,4	19,1	92,5	102,3	101,7	99,8
	0,0	0,0	0,4	23,6	90,9	103,0	99,8	98,8
	0,1	0,1	0,6	27,1	92,8	108,8	102,5	99,7
**Fadu**	0,0	0,0	0,7	82,1	93,8	95,9	102,3	99,2
	0,0	0,0	1,2	82,0	98,4	97,1	101,8	102,8
	0,1	0,1	3,1	81,5	94,9	91,4	102,8	96,2
**P.fibroblast**	0,0	0,1	102,0	107,2	102,2	105,0	100,9	102,8
	0,0	0,1	74,5	111,2	101,8	102,4	102,9	105,1
	0,1	0,1	66,0	110,4	104,3	104,2	101,8	103,6
